# A Revisit of Co(PMe_3_)_4_‑Catalyzed Hydrophosphination of Phenylacetylene with HPPh_2_

**DOI:** 10.1021/acs.organomet.6c00034

**Published:** 2026-03-23

**Authors:** J. P. I. Dulmini Jayawardhena, Jeanette A. Krause, Hairong Guan

**Affiliations:** Department of Chemistry, 2514 University of Cincinnati, Cincinnati, Ohio 45221-0172, United States

## Abstract

The low-valent cobalt complex Co­(PMe_3_)_4_ was previously reported to catalyze the hydrophosphination of phenylacetylene with HPPh_2_ to yield (*E*)-PhCH=CH­(PPh_2_) exclusively. This work provides a more in-depth analysis of the catalytic system showing that Co­(PMe_3_)_4_ is contaminated with HCo­(PMe_3_)_4_. The impurity undergoes a reversible ligand exchange reaction with HPPh_2_ to form a series of new cobalt hydrides with the formula HCo­(PMe_3_)_4–x_(HPPh_2_)_*x*_ (*x* = 1–4). One of these hydride complexes, namely, HCo­(PMe_3_)_3_(HPPh_2_), features a doublet of quartets for the hydride resonance, which was previously misassigned to the paramagnetic Co­(II) complex, HCo­(PMe_3_)_3_(PPh_2_). The contaminated Co­(PMe_3_)_4_ reacts with HPPh_2_, under heating, to generate a dinuclear cobalt complex, (Me_3_P)_2_Co­(μ-PPh_2_)_2_Co­(PMe_3_)_2_. In addition to “Co­(PMe_3_)_4_”, the independently synthesized HCo­(PMe_3_)_4_, HCo­(HPPh_2_)_4_, and (Me_3_P)_2_Co­(μ-PPh_2_)_2_Co­(PMe_3_)_2_ all prove to be catalytically competent. Regardless of the cobalt catalysts examined herein, both (Ph_2_P)­CPh=CH_2_ and (*Z*)-PhCH=CH­(PPh_2_) are present as minor hydrophosphination products.

## Graphical abstract

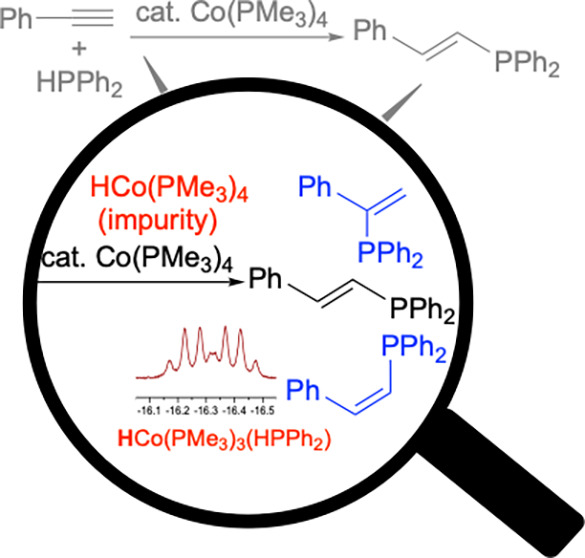

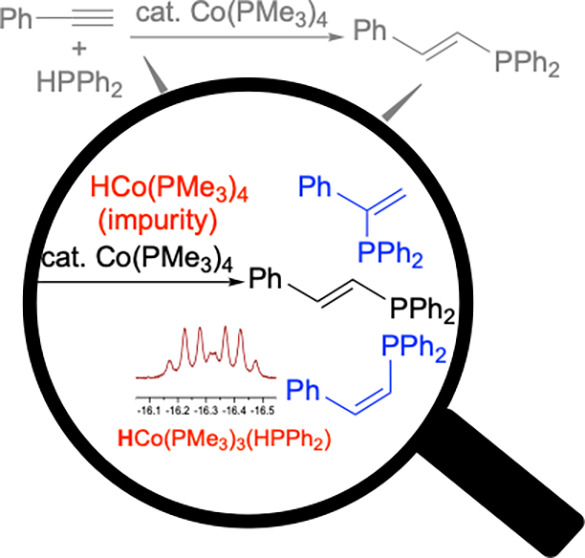

## Introduction

Hydrophosphination of alkenes and alkynes represents a streamlined approach to access phosphorus-containing molecules that in turn serve as ligands in transition metal catalysis.^1^ Although under certain conditions the P–H addition process can proceed without a catalyst,^2^ metal-catalyzed hydrophosphination reactions typically operate under milder conditions and, in many cases, form products with complementary regioselectivity and chemoselectivity. With metal-based catalysts, hydrophosphination reactions can also be rendered enantioselective, providing great opportunities to expand the toolbox of chiral phosphine ligands.

From the mechanistic point of view, metal-catalyzed hydrophosphination of alkenes or alkynes can be very complicated processes, because both the reactants (e.g., HPR_2_) and the products may compete with the supporting ligands for the binding to the metal. Due to our ongoing work focused on cobalt catalysis,^3^ we have been particularly interested in two cobalt-based catalytic systems for the hydrophosphination of alkynes with HPPh_2_: one involving Co­(acac)_2_-^*n*^BuLi (acac = acetylacetonate)^4^ and the other employing Co­(PMe_3_)_4_.^5^ While the catalytically active species for the former remains ill-defined, the mechanistic details for the latter have already been probed by Shanmugam, Shanmugam, and co-workers using NMR, EPR, and ultraviolet–visible (UV–vis) spectroscopy.^5^ The most striking spectroscopic evidence used to support P–H oxidative addition with Co­(PMe_3_)_4_ and the subsequent Ph_2_P-PPh_2_ (a byproduct) formation was the observation of a doublet of quartets at −16.35 ppm and a quintet at −17.4 ppm from the ^1^H NMR spectrum, which were assigned to HCo­(PMe_3_)_3_(PPh_2_) and H_2_Co­(PMe_3_)_4_, respectively (Scheme 1). This analysis, however, contradicts the fact that both Co­(II) hydrides are paramagnetic, which unlikely display sharp, well-resolved hydride resonances.^6^ The perfect regioselectivity that favors the β-(*E*) isomer (in almost all cases) is also intriguing, which is rarely observed with other catalytic systems, including Co­(acac)_2_-^*n*^BuLi.^4^

**Scheme 1:**
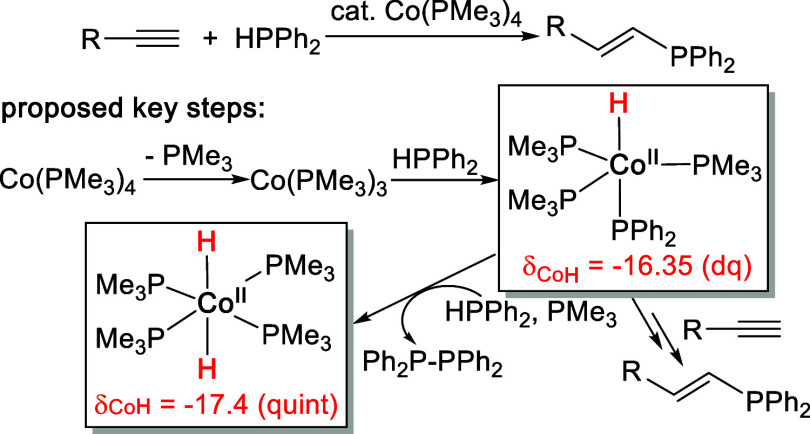
Mechanistic Pathway and Intermediates Proposed by the Shanmugam Groups^5^ for Co­(PMe_3_)_4_-Catalyzed Hydrophosphination of Alkynes with HPPh_2_

To gain a better understanding of the reaction mechanism, we conducted our own investigation of the catalytic hydrophosphination process, specifically the reaction of PhC≡CH with HPPh_2_ catalyzed by Co­(PMe_3_)_4_ as well as the related cobalt complexes. Our results, as summarized below, raise awareness of the purity issue with Co­(PMe_3_)_4_, provide a reinterpretation of the puzzling NMR data described earlier, and call attention to a number of other cobalt species that can also be responsible for the catalytic hydrophosphination reaction. In addition, our study suggests that the regioselectivity is not as simple as previously thought, requiring a reanalysis of hydrophosphination products generated from other terminal alkynes.

## Results and Discussion

### Purity Issue with Co­(PMe_3_)_4_

Co­(PMe_3_)_4_ can be made through the reduction of CoCl_2_ with Mg(0) in the presence of PMe_3_. The synthetic procedure was originally developed by Klein and Karsch^7^ and subsequently adopted by many other research groups.^8^ This homoleptic Co(0) complex should be paramagnetic, as confirmed by Klein,^9^ although no NMR data were provided in the original report. The NMR spectra of “Co­(PMe_3_)_4_” supplied by the Shanmugam groups, however, showed a sharp proton resonance at 1.26 ppm (in C_6_D_6_) and, more surprisingly, a broad phosphorus resonance around −1 ppm.^5^ Similar chemical shift values were described by Zhang and co-workers for their “Co­(PMe_3_)_4_” sample.^8f^ We recognized these spectroscopic features as those of HCo­(PMe_3_)_4_,^10^ a *diamagnetic* reagent that we had used extensively to prepare various cobalt pincer complexes.^3a–3c^ It is possible that these research groups did not increase the spectral width to allow the more characteristic hydride resonance to be observed. This prompted us to repeat the synthesis of Co­(PMe_3_)_4_ using the Mg(0) reduction method mentioned above.^7^ The NMR spectra/data for our sample in fact match those reported by Shanmugam, Shanmugam, and Zhang, provided that we only focus on a relatively narrow spectral window (e.g., +10 ppm to −1 ppm for the ^1^H NMR spectrum, see Figure S1 in the Supporting Information). Expanding the ^1^H NMR spectral window (+100 ppm to −60 ppm, see Figure S2) uncovered the hydride resonance of HCo­(PMe_3_)_4_ at −17.33 ppm as a quintet (*J*_H–P_ = 31.2 Hz) and a broad resonance at 33.1 ppm (*w*_1/2_ = 154 Hz). The latter is the real ^1^H NMR signal of Co­(PMe_3_)_4_.^8c,8g^ As expected, the ^31^P­{^1^H} NMR spectrum with an expanded spectral window showed only a weak and broad resonance around −1 ppm, consistent with a paramagnetic compound containing HCo­(PMe_3_)_4_ as a diamagnetic impurity. The formation of HCo­(PMe_3_)_4_ can be rationalized by overreduction of CoCl_2_/PMe_3_ to [Co­(PMe_3_)_4_]^−^ followed by protonation with adventitious water (Scheme 2).^11,12^ Alternatively, Co­(PMe_3_)_4_ could abstract a hydrogen atom from water to yield HCo­(PMe_3_)_4_ and, presumably, (HO)­Co­(PMe_3_)_3_, along with PMe_3_. To test these hypotheses, the reaction vessel and solvent used in the synthesis were thoroughly dried, which resulted in less contamination. A control experiment with our best “Co­(PMe_3_)_4_” sample (in C_6_D_6_) indicated that the amount of HCo­(PMe_3_)_4_ increased upon addition of H_2_O (5 equiv). Unfortunately, despite repeated trials, HCo­(PMe_3_)_4_ remained present as an impurity^13^ and our attempts to purify Co­(PMe_3_)_4_ via recrystallization or sublimation were unsuccessful.

**Scheme 2:**
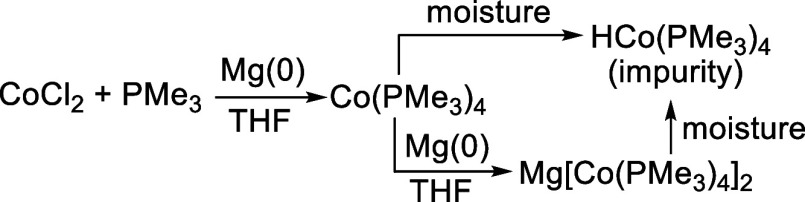
Proposed Formation of HCo­(PMe_3_)_4_ as an Impurity in Co­(PMe_3_)_4_

### Reactions of “Co­(PMe_3_)_4_” (or Contaminated Co­(PMe_3_)_4_) with HPPh_2_

To revisit the P–H oxidative addition step described by the Shanmugam groups, we treated the contaminated Co­(PMe_3_)_4_ sample (dissolved in C_6_D_6_) with 1 equiv of HPPh_2_, which resulted in an immediate color change from orange to dark brown. Monitoring the reaction by ^1^H NMR spectroscopy indicated that Co­(PMe_3_)_4_ was consumed within 2 h to form a new species with paramagnetically shifted resonances at 9.10, 8.74, 4.09, and ∼1 ppm (overlapped with the resonances of several diamagnetic, PMe_3_-containing compounds). The hydride region of the NMR spectra featured a doublet of quartets at −16.32 ppm (*J* = 57.6 and 21.6 Hz) and a triplet of triplets at −15.52 ppm (*J* = 49.6 and 6.0 Hz), in addition to the quintet at −17.33 ppm (*J* = 31.2 Hz) for unreacted HCo­(PMe_3_)_4_. It is worth pointing out here that the doublet of quartets matches the one described by the Shanmugam groups.^5^ The ^31^P­{^1^H} NMR spectra of the sample showed the unreacted HCo­(PMe_3_)_4_ and HPPh_2_ (−40.5 ppm), free PMe_3_ (−62.4 ppm), several broad resonances in the 0–40 ppm range, and a small amount of Ph_2_P-PPh_2_ (−14.8 ppm, ∼1%).

Given that HCo­(PMe_3_)_4_ is an impurity present in Co­(PMe_3_)_4_, the doublet of quartets at −16.32 ppm observed by us and the Shanmugam groups could originate from a ligand substitution reaction of HCo­(PMe_3_)_4_ with HPPh_2_. This was confirmed by an NMR experiment starting from pure HCo­(PMe_3_)_4_ (prepared from Co­(acac)_2_, PMe_3_, and NaBH_4_ ^10^) mixed with 10 equiv of HPPh_2_ in C_6_D_6_. As illustrated in Figure 1, the hydride resonance of HCo­(PMe_3_)_4_ gradually disappeared over time, replaced with the aforementioned doublet of quartets at −16.32 ppm, the triplet of triplets at −15.52 ppm, and a further downfield shifted hydride resonance at −14.79 ppm as a quartet of doublets (*J* = 44.6 and 31.4 Hz). Heating the sample to 80 °C revealed that yet another cobalt hydride emerged further downfield, at −14.18 ppm. The splitting patterns and chemical shift values for these hydride resonances are consistent with a series of HCo­(PMe_3_)_4–*x*_(HPPh_2_)_*x*_ (*x* = 1–4) resulting from consecutive substitution of PMe_3_ by HPPh_2_ (Scheme 3). The fully substituted complex, HCo­(HPPh_2_)_4_, is a known compound, which was previously obtained from reduction of Co­(acac)_3_-HPPh_2_ with Al­(^*i*^Bu)_3_.^14^ To avoid using the organoaluminum reagent, which is discontinued from many vendors, we developed a new synthetic method that involved reduction of Co­(acac)_2_-HPPh_2_ with NaBEt_3_H. This allowed us to probe the equilibria in Scheme 3 from the reverse direction, although substitution of the phosphine ligands in HCo­(HPPh_2_)_4_ by PMe_3_ was found to be substantially slower (Figures S10 and S11).

**Figure 1:**
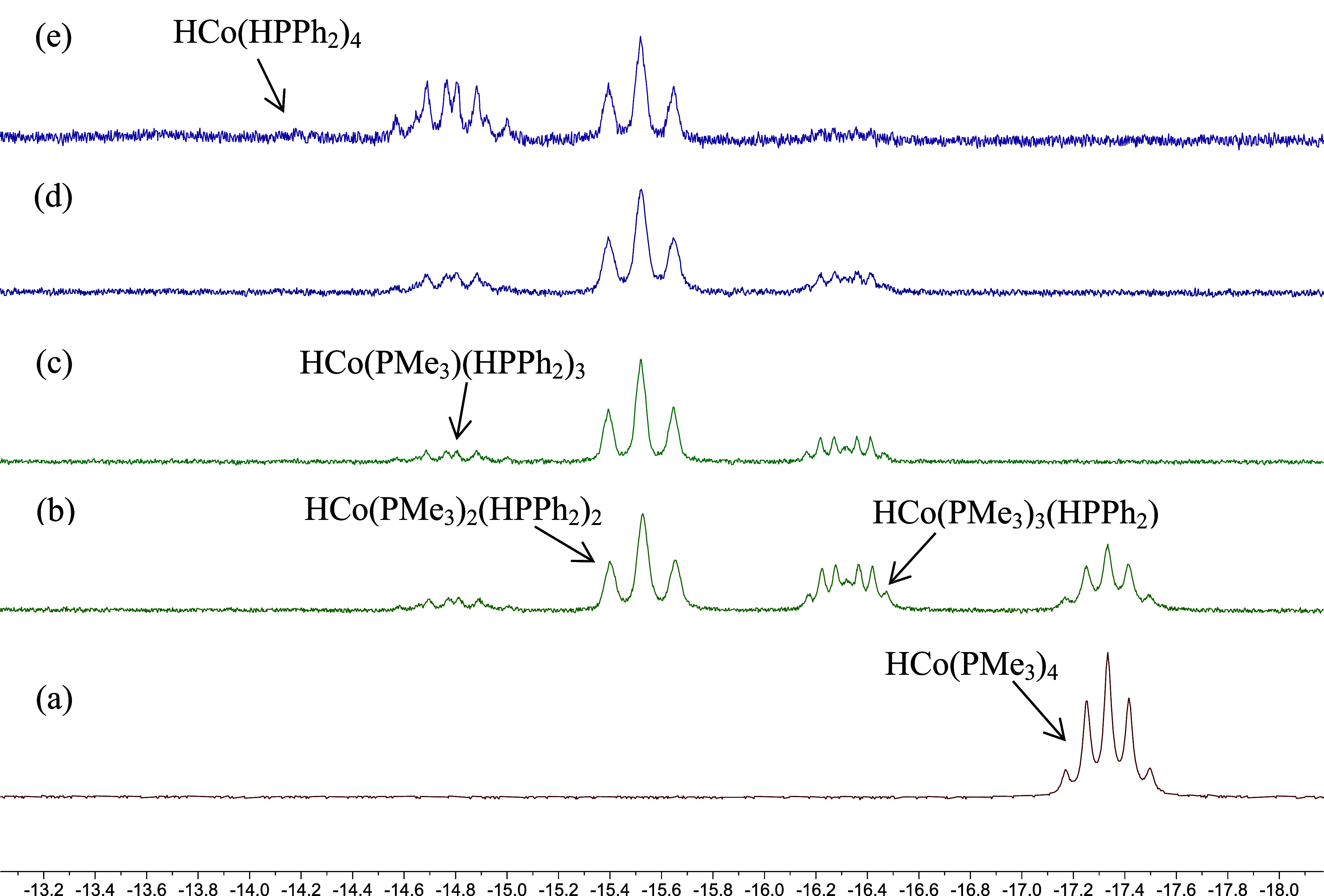
Hydride region of the ^1^H NMR (400 MHz, in C_6_D_6_) spectra of (a) HCo­(PMe_3_)_4_; (b) HCo­(PMe_3_)_4_-HPPh_2_ (1:10) kept at 23 °C for 1 h; (c) HCo­(PMe_3_)_4_-HPPh_2_ (1:10) kept at 23 °C for 24 h; (d) HCo­(PMe_3_)_4_-HPPh_2_ (1:10) kept at 23 °C for 24 h and then heated at 80 °C for 1 h; (e) HCo­(PMe_3_)_4_-HPPh_2_ (1:10) kept at 23 °C for 24 h and then heated at 80 °C for 48 h.

**Scheme 3:**
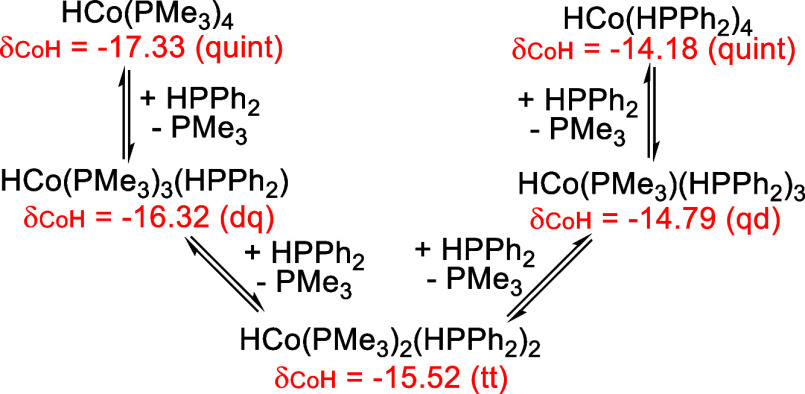
Ligand Substitution Reactions Involving HCo­(PMe_3_)_4–*x*_(HPPh_2_)_*x*_ (*x* = 0–4)

The reaction of “Co­(PMe_3_)_4_” with 1 equiv of HPPh_2_, when performed at 80 °C, gave a significantly darker solution. Its NMR spectra showed that the initially formed paramagnetic species slowly disappeared with concomitant formation of H_2_ (4.47 ppm) and a diamagnetic compound displaying phosphorus resonances at 138.8 and −4.3 ppm (Figure S4).^15^ The reaction mixture eventually produced some black crystals which, according to our X-ray diffraction study (Figure 2), are for the dinuclear phosphido complex, (Me_3_P)_2_Co­(μ-PPh_2_)_2_Co­(PMe_3_)_2_.^16^ Our NMR analysis of the crystals redissolved in C_6_D_6_ confirmed that the resonances at 138.8 and −4.3 ppm correspond to the bridging PPh_2_ and PMe_3_, respectively. A previous study of this compound reported phosphorus resonances (12.3 and 4.7 ppm, in THF-*d*_8_)^16a^ drastically different from ours, although our data (Figures S14 and S16), especially the chemical shift for the bridging PPh_2_ group, is closer to the one reported for the analogous complex, (Ph_2_PNHPPh_2_)­Co­(μ-PPh_2_)_2_Co­(Ph_2_PNHPPh_2_) (δ_**P**Ph2_ = 112.2 ppm).^17^

**Figure 2:**
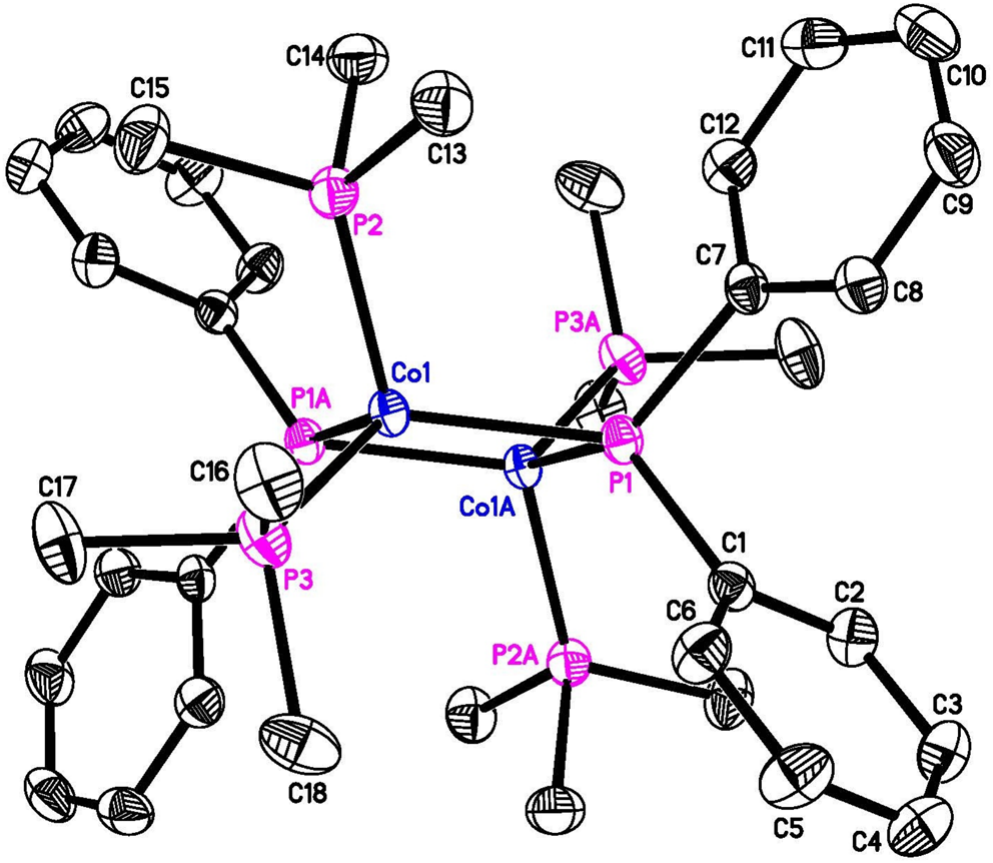
ORTEP of (Me_3_P)_2_Co­(μ-PPh_2_)_2_Co­(PMe_3_)_2_ at the 50% probability level (for clarity, hydrogen atoms are omitted, only one of two independent molecules is shown, and only symmetry-unique carbon atoms are labeled).

Considering the ability of Co­(PMe_3_)_4_ to activate various chemical bonds,^18^ including P–H bonds,^19^ we propose that the initially formed paramagnetic product is HCo^II^(PMe_3_)_3_(PPh_2_) resulting from P–H oxidative addition on Co(0) (Scheme 4).^20^ This is the same intermediate proposed by the Shanmugam groups (Scheme 1)^5^ but mischaracterized using the diamagnetic NMR signals that belong to HCo^I^(PMe_3_)_3_(HPPh_2_). Upon heating, the paramagnetic HCo^II^(PMe_3_)_3_(PPh_2_) loses its hydride ligand in the form of H_2_ and the resulting Co­(PMe_3_)_3_(PPh_2_) rapidly dimerizes to yield (Me_3_P)_2_Co­(μ-PPh_2_)_2_Co­(PMe_3_)_2_. A similar mechanism has been proposed for the formation of (Me_3_P)_2_Co­(μ-SiHPh_2_)_2_Co­(PMe_3_)_2_ from the reaction between Co­(PMe_3_)_4_ and Ph_2_SiH_2_.^21^ First-row transition metals are known to promote dehydrocoupling of HPPh_2_ via phosphido intermediates, either independently or as a catalyst activation step in hydrophosphination reactions.^22^ Such a process is not a major pathway in the system studied here, as the formation of Ph_2_P-PPh_2_ is negligible (<2%). We noticed that the PMe_3_ resonance of (Me_3_P)_2_Co­(μ-PPh_2_)_2_Co­(PMe_3_)_2_ also appeared in the NMR spectra provided by the Shanmugam groups for their reaction of “Co­(PMe_3_)_4_” with HPPh_2_ (Figure S32). The ligand substitution reactions between HCo­(PMe_3_)_4_ and HPPh_2_ (10 equiv) or between HCo­(HPPh_2_)_4_ and PMe_3_ (10 equiv) also produced (Me_3_P)_2_Co­(μ-PPh_2_)_2_Co­(PMe_3_)_2_ at 80 °C, which contributed to the darkening of the reaction mixtures and reduced the NMR signal-to-noise ratios (due to precipitation of the dinuclear species).

**Scheme 4:**
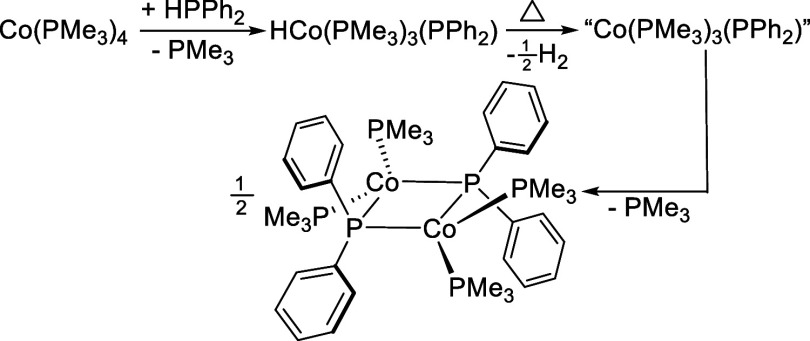
Proposed Reaction Pathway Leading to (Me_3_P)_2_Co­(μ-PPh_2_)_2_Co­(PMe_3_)_2_

### Catalytic Reactions

Next, we re-examined “Co­(PMe_3_)_4_”-catalyzed hydrophosphination of PhC≡CH with HPPh_2_ using the same procedures developed by the Shanmugam groups.^5^ The protocol requires a premix of “Co­(PMe_3_)_4_” and HPPh_2_ (20 equiv) at room temperature for 10 min, followed by the addition of PhC≡CH (20 equiv) and then heating at 80 °C for 2 h. To facilitate purification and characterization, the crude products are typically converted to the corresponding phosphine sulfides prior to NMR analysis (Scheme 5). The original study reported that (*E*)-PhCH=CH­(P=SPh_2_) formed exclusively as the *P*-protected hydrophosphination product.^5^ However, in our hands, both the β-(*E*) and α isomers were obtained with a ratio of 82:18 (Figure S17). The third isomer, (*Z*)-PhCH=CH­(P=SPh_2_),^2b^ was negligible but detectable by NMR. The two major products, namely (*E*)-PhCH=CH­(P=SPh_2_) and (Ph_2_P=S)­CPh=CH_2_, were separated and purified by column chromatography. The isolation of the α isomer is noteworthy, as very few catalytic systems produce appreciable amounts of the Markovnikov product.^1f^

**Scheme 5:**
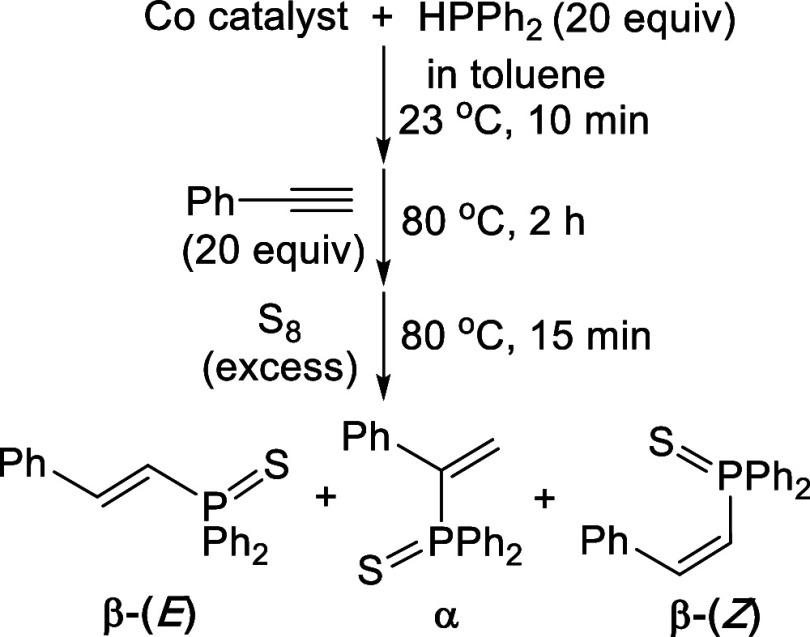
Standard Procedures for Cobalt-Catalyzed Hydrophosphination of PhC≡CH with HPPh_2_

The purity issue with Co­(PMe_3_)_4_ and the outcome for the reaction of “Co­(PMe_3_)_4_” with HPPh_2_ raised the obvious question: could the impurity HCo­(PMe_3_)_4_ and/or the cobalt complexes derived from HPPh_2_ be responsible for the hydrophosphination reaction? Addressing the roles played by the paramagnetic intermediates as well as HCo­(PMe_3_)_4–*x*_(HPPh_2_)_*x*_ (*x* = 1–3) is challenging due to the difficulty in isolating these transient species. However, we were able to examine the catalytic activity of synthetically accessible compounds, including HCo­(PMe_3_)_4_, HCo­(HPPh_2_)_4_, and (Me_3_P)_2_Co­(μ-PPh_2_)_2_Co­(PMe_3_)_2_. Applying the standard procedures (Scheme 5) to these cobalt complexes indicated that they are all catalytically competent. As summarized in Table 1, HCo­(PMe_3_)_4_ gave virtually the same regioselectivity as “Co­(PMe_3_)_4_” (entry 1 vs entry 2). In contrast, using HCo­(HPPh_2_)_4_ as a catalyst resulted in more α isomer, although the β-(*E*) isomer remained to be the dominant product (entry 3). Interestingly, when the reaction was catalyzed by (Me_3_P)_2_Co­(μ-PPh_2_)_2_Co­(PMe_3_)_2_, (*Z*)-PhCH=CH­(P=SPh_2_) represented 6% of the total *P*-protected hydrophosphination products (entry 4). Amatore, Aubert, Petit, and co-workers have shown that both Co­(PMe_3_)_4_ and HCo­(PMe_3_)_4_ can catalyze the dimerization of PhC≡CH to yield (*E*)-PhC≡CCH=CHPh and PhC≡CCPh=CH_2_.^23^ These enynes also appeared as byproducts in our catalytic reactions, although the conversion of PhC≡CH to these molecules was too low (<10%) to be considered competitive.

**Table 1:** Summary of Regioselectivity Observed with Different Cobalt Catalysts^a^

entry	cobalt catalyst	catalyst loading	β-(*E*): α: β-(*Z*)
1	“Co(PMe_3_)_4_”	5 mol % Co	82:18: trace
2	HCo(PMe_3_)_4_	5 mol %	83:17: trace
3	HCo(HPPh_2_)_4_	5 mol %	70:30: trace
4	(Me_3_P)_2_Co(μ-PPh_2_)_2_Co(PMe_3_)_2_	2.5 mol %	78:16:6

a Reaction conditions: PhC≡CH (0.90 mmol), HPPh_2_ (0.90 mmol), and a cobalt catalyst (0.045 mmol Co) in toluene (3 mL), 80 °C, 2 h. The products were treated with S_8_ (1.88 mmol S) and analyzed by ^1^H and ^31^P­{^1^H} NMR spectroscopy.

The phosphorus-containing compounds generated from posthydrophosphination treatment with S_8_ can be accounted for using ^31^P­{^1^H} NMR data reported in the literature. This includes not only the *P*-protected hydrophosphination products, i.e., (*E*)-PhCH=CH­(P=SPh_2_),^5^ (*Z*)-PhCH=CH­(P=SPh_2_),^2b^ and (Ph_2_P=S)­CPh=CH_2_ ^4^ but also (Ph_2_P=S)–S-S-(P=SPh_2_),^24^ (Ph_2_P=S)_2_,^25^ (Ph_2_P=S)-S-(P=SPh_2_),^26^ and Ph_3_P=S,^27^ which result from interactions of S_8_ with the unreacted HPPh_2_ and the minor byproducts (i.e., Ph_2_P-PPh_2_ and Ph_3_P). The diphosphine sulfide, (Ph_2_P=S)­CH_2_CHPh­(P=SPh_2_),^28^ which could potentially form via two consecutive hydrophosphination steps, was not observed. For the reaction catalyzed by HCo­(HPPh_2_)_4_ (Table 1, entry 3), we also obtained some dark-green crystals, which were analyzed as (Ph_2_PS_2_)_4_Co_2_ (Figure 3). The color matches the independently synthesized (Ph_2_PS_2_)_2_Co,^29^ and the dimeric structure resembles what was reported for the ethyl analog, (Et_2_PS_2_)_4_Co_2_.^30^

**Figure 3:**
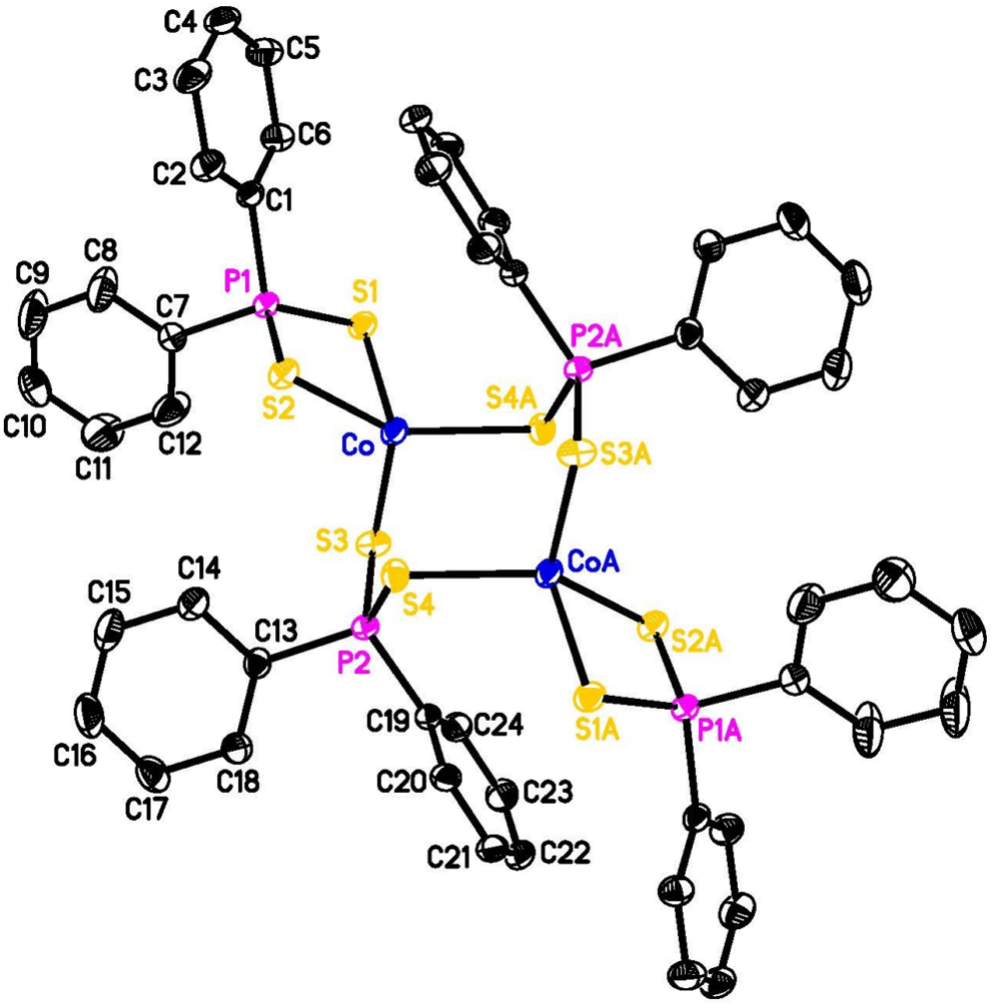
ORTEP of (Ph_2_PS_2_)_4_Co_2_ at the 50% probability level (for clarity, hydrogen atoms are omitted and only symmetry-unique carbon atoms are labeled).

The addition of S_8_ should have no effect on the regioselectivity. To support this assertion, “Co­(PMe_3_)_4_”-catalyzed hydrophosphination of PhC≡CH with HPPh_2_ was carried out in toluene-*d*_8_ following the standard procedures but without the post-treatment with S_8_. Monitoring the progress of the reaction by NMR suggested that the hydrophosphination process was complete in 2 h, producing a 82:17:1 mixture of (*E*)-PhCH=CH­(PPh_2_),^31^ (Ph_2_P)­CPh=CH_2_,^31a^ and (*Z*)-PhCH=CH­(PPh_2_)^31^ (Figure S27). This regioselectivity is almost identical to the one analyzed after the initial products were converted to phosphine sulfides (Table 1, entry 1). When the catalytic reaction was performed at room temperature (23 °C), selectivity for (*E*)-PhCH=CH­(PPh_2_) slightly improved (β-(*E*): α: β-(*Z*) = 88:11:1, see Figure S28), although the reaction was significantly slower (64% conversion in 24 h). In another experiment, “Co­(PMe_3_)_4_” was pretreated with HPPh_2_ at 80 °C for 24 h before PhC≡CH was introduced into the reaction mixture (Scheme 6). This modified protocol allowed more “Co­(PMe_3_)_4_” to be converted to (Me_3_P)_2_Co­(μ-PPh_2_)_2_Co­(PMe_3_)_2_ while delaying the onset of the catalytic hydrophosphination reaction. The products consisted of (*E*)-PhCH=CH­(PPh_2_), (Ph_2_P)­CPh=CH_2_, and (*Z*)-PhCH=CH­(PPh_2_) with a ratio of 81:16:3 (Figure S29), which is similar to the regioselectivity achieved by (Me_3_P)_2_Co­(μ-PPh_2_)_2_Co­(PMe_3_)_2_ only (Table 1, entry 4). Of the catalytic reactions examined here, almost all produced detectable amounts of Ph_2_P-PPh_2_ and PPh_3_ as the minor byproducts, which were also observed from the reaction of “Co­(PMe_3_)_4_” with HPPh_2_, especially under heating. Dehydrocoupling of primary or secondary phosphines to form P–P bonds is well precedented with cobalt complexes,^32^ and P–C bond cleavage by Co­(PMe_3_)_4_ is also known in the literature.^33^

**Scheme 6:**
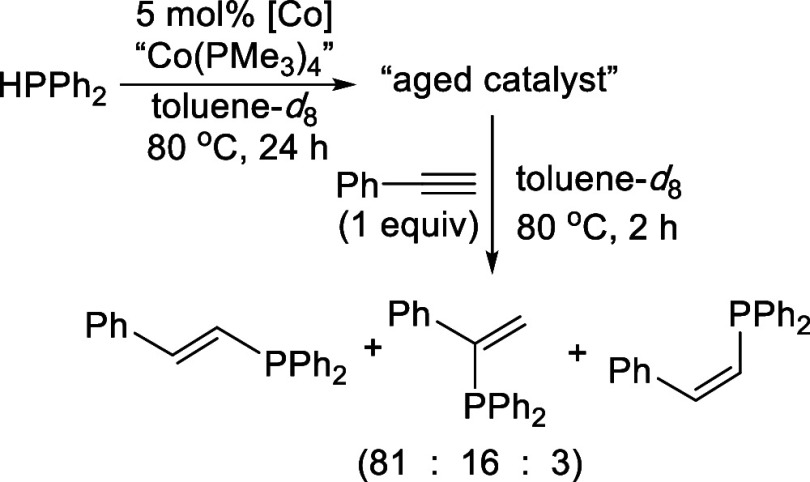
Hydrophosphination of PhC≡CH with HPPh_2_ Promoted by an Aged Catalyst

### Final Thoughts

As shown in Table 1, HCo­(HPPh_2_)_4_ and (Me_3_P)_2_Co­(μ-PPh_2_)_2_Co­(PMe_3_)_2_ catalyze the hydrophosphination reaction with regioselectivity markedly different from that achieved by HCo­(PMe_3_)_4_ and “Co­(PMe_3_)_4_”. This suggests that HCo­(HPPh_2_)_4_ and (Me_3_P)_2_Co­(μ-PPh_2_)_2_Co­(PMe_3_)_2_ operate by a different catalytic mechanism. In fact, the ligand substitution reaction of HCo­(PMe_3_)_4_ leading to HCo­(HPPh_2_)_4_ and the P–H bond activation with “Co­(PMe_3_)_4_” to yield (Me_3_P)_2_Co­(μ-PPh_2_)_2_Co­(PMe_3_)_2_ are considerably slower than the catalytic reaction (>24 h vs 2 h at 80 °C). The same regioselectivity displayed by HCo­(PMe_3_)_4_ and “Co­(PMe_3_)_4_” is somewhat surprising. A control experiment focusing on the thermal stability of HCo­(PMe_3_)_4_ (in C_6_D_6_) revealed that, at 80 °C, HCo­(PMe_3_)_4_ gradually lost its hydride resonance, although the ^1^H and ^31^P resonances associated with PMe_3_ were almost intact (Figures S30 and S31).^34^ Under the same conditions, “Co­(PMe_3_)_4_” showed no sign of degradation except the NMR signals attributed to the impurity HCo­(PMe_3_)_4_. For the hydrophosphination reaction catalyzed by HCo­(PMe_3_)_4_, thermal decomposition of cobalt hydride may initiate the catalytic process. It is also possible that the hydrophosphination reaction proceeds via an initial insertion of PhC≡CH into HCo­(PMe_3_)_4_, with regioselectivity coincidentally identical to the alkyne insertion into the putative intermediate, HCo^II^(PMe_3_)_3_(PPh_2_) (produced from Co­(PMe_3_)_4_). At the moment, the complexity of the catalytic system does not allow us to differentiate these mechanistic pathways.

In any case, the formation of the α- and β-(*Z*) hydrophosphination products is evident and perhaps overlooked in the previous studies by the Shanmugam groups. The NMR spectra provided in the original work involving “Co­(PMe_3_)_4_”^5^ and a related study (about a dinuclear complex derived from “Co­(PMe_3_)_4_”)^35^ were mostly for the products purified by column chromatography. Nevertheless, we note that at least three examples show the α isomer as the impurity in (*E*)-RCH=CH­(P=SPh_2_); the α isomer is readily detectable due to its two characteristic vinylic hydrogens appearing in the 5.5–6.5 ppm region (two doublets with different ^3^ *J*_H–P_ values). In other words, the regioselectivity for each substrate needs to be re-examined.

## Conclusions

Through this work, we have demonstrated that HCo­(PMe_3_)_4_ is an almost unavoidable impurity in Co­(PMe_3_)_4_. The contamination may go unnoticed or is not an issue under the circumstances where HCo­(PMe_3_)_4_ and “Co­(PMe_3_)_4_” exhibit similar reactivity;^8b,23,36^ however, it could cause complications for synthesis when the Co­(I) and Co(0) species behave differently. For the hydrophosphination of PhC≡CH with HPPh_2_ catalyzed by “Co­(PMe_3_)_4_”, the impurity issue has led to the misinterpretation of the doublet of quartets at −16.35 ppm (in C_6_D_6_) to be the hydride resonance for HCo^II^(PMe_3_)_3_(PPh_2_) (Scheme 1).^5^ Our ligand substitution experiments have confirmed that this hydride resonance belongs to HCo^I^(PMe_3_)_3_(HPPh_2_), which originates from the reaction of the impurity HCo­(PMe_3_)_4_ with HPPh_2_. Similarly, the quintet at −17.4 ppm was misassigned to H_2_Co^II^(PMe_3_)_4_. It became more conspicuous when the alkyne substrate was added to the mixture of “Co­(PMe_3_)_4_” and HPPh_2_. We believe that this quintet is for the hydride resonance of HCo^I^(PMe_3_)_4_, which re-emerged in the reaction mixture due to the depletion of HPPh_2_ (i.e., HCo^I^(PMe_3_)_3_(HPPh_2_) was reverted to HCo^I^(PMe_3_)_4_). Furthermore, our mechanistic investigation has suggested that the hydrophosphination process is not as selective as previously described. In addition to the β-(*E*) products, the α isomers form in appreciable amounts as the second major products. The independently synthesized HCo­(PMe_3_)_4_, HCo­(HPPh_2_)_4_, and (Me_3_P)_2_Co­(μ-PPh_2_)_2_Co­(PMe_3_)_2_ all prove to be competent catalysts for the hydrophosphination reaction. These species are present in the reaction of “Co­(PMe_3_)_4_” with HPPh_2_, though at different stages, thus complicating the analysis of the catalytic roles played by Co­(PMe_3_)_4_. This work, however, does not refute the previous claim that Co­(PMe_3_)_4_ cleaves the P–H bond of HPPh_2_ and the resulting HCo^II^(PMe_3_)_3_(PPh_2_) initiates the catalytic hydrophosphination reaction.

## Experimental Section

### General Methods

Unless otherwise noted, all organometallic compounds were prepared and handled under an argon atmosphere using standard glovebox and Schlenk techniques. Dry and oxygen-free toluene and pentane were collected from an Innovative Technology solvent purification system and used throughout the experiments. Anhydrous and inhibitor-free tetrahydrofuran (THF) was purchased from Sigma-Aldrich (packed in a Sure/Seal bottle) and used as received without further purification. HCo­(PMe_3_)_4_ ^10^ and “Co­(PMe_3_)_4_” (quotation marks used to indicate that Co­(PMe_3_)_4_ is contaminated with HCo­(PMe_3_)_4_)^7^ were prepared as described in the literature. Benzene-*d*_6_ (99.5% D) was dried over sodium benzophenone and distilled under argon. Methylene chloride-*d*_2_ (99.8% D), toluene-*d*_8_ (99.5% D), THF-*d*_8_ (99.5% D), and chloroform-*d*_1_ (99.8% D, with 0.03% v/v TMS) were purchased from Cambridge Isotope Laboratories, Inc. and used as received. NMR spectra were recorded at 23 °C on a Bruker AV400 or NEO400 NMR spectrometer. Chemical shift values for ^1^H and ^13^C­{^1^H} NMR spectra were referenced internally to the residual solvent resonances. ^31^P­{^1^H} NMR spectra were referenced externally to 85% H_3_PO_4_ (0 ppm). IR spectra were recorded on a PerkinElmer Spectrum Two Fourier Transform infrared spectrometer equipped with a Smart Orbit diamond attenuated-total-reflectance (ATR) accessory.

### Synthesis of HCo­(HPPh_2_)_4_

To an oven-dried Schlenk flask equipped with a stir bar were added Co­(acac)_2_ (375 mg, 1.46 mmol), THF (6 mL), and HPPh_2_ (1.00 mL, 5.75 mmol). The mixture was stirred at room temperature for 15 min, after which a 1.0 M THF solution of NaBEt_3_H (2.91 mL, 2.91 mmol) was added, resulting in an immediate color change from pinkish purple to dark brown. The reaction mixture was stirred overnight and then pumped to dryness to afford a brown residue, which was washed with pentane (10 mL × 5) and dried under vacuum. The crude product was extracted with toluene (10 mL × 4) and passed through a pad of Celite. The collected toluene solution was concentrated under vacuum to yield a brown residue, which was once again washed with pentane (10 mL × 4) followed by drying under vacuum. The desired product was isolated as an orange solid (757 mg, 65% yield). ^1^H NMR (400 MHz, C_6_D_6_, δ): 7.50–7.30 (br, Ar*H*, 16H), 7.04–6.92 (br, Ar*H*, 24H), 6.55 (dm, ^1^ *J*_H–P_ = 314 Hz, 4H), –14.18 (quint, ^2^ *J*_H–P_ = 25.2 Hz, Co*H*, 1H). ^13^C­{^1^H} NMR (101 MHz, C_6_D_6_, δ): 140.0–139.3 (m, *C*_ipso_), 133.0–132.7 (m, *C*_ortho_ or *C*_meta_); the other two carbon resonances are obscured by the solvent resonance. ^13^C­{^1^H} NMR (101 MHz, CD_2_Cl_2_, δ): 140.0–139.3 (m, *C*_ipso_), 132.7–132.3 (m, *C*_ortho_ or *C*_meta_), 128.4–128.2 (m, *C*_ortho_ or *C*_meta_), 128.1 (s, *C*_para_). Note: this cobalt hydride decomposes in CD_2_Cl_2_ to form some paramagnetic species along with free HPPh_2_. Nevertheless, the decomposition process was slow enough to allow us to identify all four resonances from the ^13^C­{^1^H} NMR spectrum. ^31^P­{^1^H} NMR (162 MHz, C_6_D_6_, δ): 38.8 (br). Selected ATR-IR data (solid, cm^–1^): 3049, 2263 (ν_P–H_), 2217 (ν_P–H_), 1910 (ν_Co–H_), 1582, 1478, 1432, 1305, 1179, 1092, 868, 734, 691, 501, 471. Anal. Calcd for C_48_H_45_CoP_4_: C, 71.64; H, 5.64. Found: C, 71.90; H, 5.54.

### Synthesis of (Me_3_P)_2_Co­(μ-PPh_2_)_2_Co­(PMe_3_)_2_

To an oven-dried Schlenk tube equipped with a stir bar were added “Co­(PMe_3_)_4_” (120 mg, 0.33 mmol of Co) and toluene (5 mL), followed by HPPh_2_ (98% purity, 59.6 μL, 0.33 mmol), which resulted in an immediate color change from orange to dark brown. The resulting mixture was heated at 80 °C for 24 h and then concentrated to ∼1 mL under a reduced pressure. The residue was layered with pentane (5 mL) and stored in a −30 °C freezer until a dark brown precipitate formed. The precipitate was collected by filtration, washed with pentane (3 mL × 3), and dried under vacuum to yield the desired dinuclear complex (44 mg, 34% yield). ^1^H NMR (400 MHz, C_6_D_6_, δ): 7.83–7.79 (br, Ar*H*, 8H), 7.15–7.12 (m, Ar*H*, 12H), 0.89 (br, P­(C*H*_3_)_3_, 16H). ^1^H NMR (400 MHz, THF-*d*_8_, δ): 7.57–7.53 (br, Ar*H*, 8H), 7.10–7.06 (m, Ar*H*, 12H), 0.89 (br, P­(C*H*_3_)_3_, 16H). ^31^P­{^1^H} NMR (162 MHz, C_6_D_6_, δ): 139.1 (br, *P*Ph_2_, 2P), –4.4 (br, *P*Me_3_, 4P). ^31^P­{^1^H} NMR (162 MHz, THF-*d*_8_, δ): 138.4 (br, *P*Ph_2_, 2P), –4.3 (br, *P*Me_3_, 4P).

### General Procedure for Cobalt-Catalyzed Hydrophosphination of Phenylacetylene

To an oven-dried Schlenk tube equipped with a stir bar were added “Co­(PMe_3_)_4_” (16.5 mg, 0.045 mmol Co, 5 mol % catalyst loading) and toluene (3 mL), followed by HPPh_2_ (98% purity, 0.16 mL, 0.90 mmol), which resulted in an immediate color change from orange to dark brown. The resulting mixture was stirred at room temperature for 10 min prior to the addition of phenylacetylene (98% purity, 101 μL, 0.90 mmol) and then heated to 80 °C for 2 h. After cooling to room temperature, S_8_ (60 mg, 1.88 mmol S) was added, and the reaction mixture was heated again at 80 °C for 15 min and then poured into ∼10 mL of deionized water. The crude product, a dark brown oily residue, was obtained via extraction with ethyl acetate (10 mL × 3) followed by removal of the solvent from the combined organic layers. The two major hydrophosphination products (α and β-(*E*) isomers with a ratio of 18:82) were separated by column chromatography using hexanes/ethyl acetate (99:1) as the eluent. The regioselectivity for the hydrophosphination process was determined by NMR analysis of the crude products obtained prior to the ethyl acetate extraction step. The same procedure was applied to the reactions catalyzed by HCo­(PMe_3_)_4_, HCo­(HPPh_2_)_4_, and (Me_3_P)_2_Co­(μ-PPh_2_)_2_Co­(PMe_3_)_2_ (5 mol % Co in each case). The results are provided in the Supporting Information (Figures S18–S20).

#### Characterization Data of Diphenyl­(1-phenylethenyl)­phosphine Sulfide (Eluted Out First)

A yellow oil (30 mg, 10% yield). ^1^H NMR (400 MHz, CDCl_3_, δ): 7.90–7.79 (m, Ar*H*, 4H), 7.54–7.35 (m, Ar*H*, 8H), 7.25–7.16 (m, Ar*H*, 3H), 6.19 (d, ^3^ *J*_H–P_ = 42.4 Hz, vinylic C*H*, 1H), 5.79 (d, ^3^ *J*_H–P_ = 20.8 Hz, vinylic C*H*, 1H). ^13^C­{^1^H} NMR (101 MHz, CDCl_3_, δ): 144.5 (d, ^1^ *J*_C–P_ = 73.3 Hz, (Ph_2_PS)*C*(Ph)=CH_2_), 137.4 (d, *J*_C–P_ = 10.6 Hz), 132.4 (d, *J*_C–P_ = 10.5 Hz), 131.7 (d, *J*_C–P_ = 10.5 Hz), 131.6 (d, *J*_C–P_ = 3.3 Hz), 131.5 (d, ^1^ *J*_C–P_ = 84.6 Hz, *C*_ipso_), 128.6 (d, *J*_C–P_ = 4.7 Hz), 128.4 (d, *J*_C–P_ = 12.8 Hz), 128.2 (d, ^4^ *J*_C–P_ = 0.9 Hz), 128.1. ^31^P­{^1^H} NMR (162 MHz, CDCl_3_, δ): 44.1. Selected ATR-IR data (neat, cm^–1^): 3056, 1436, 1097, 1028, 774, 748, 723, 711, 690, and 667. The ^1^H NMR data match those reported in the literature.^4^

#### Characterization Data of (*E*)-Diphenyl­(2-phenylethenyl)­phosphine Sulfide (Eluted Out Next)

A white solid was obtained (100 mg, 35% yield). ^1^H NMR (400 MHz, CDCl_3_, δ): 7.86–7.75 (m, ArH, 4H), 7.59 (dd, *J*_H–P_ = 22.8 Hz, ^3^ *J*_H–H_ = 16.8 Hz, vinylic C*H*, 1H), 7.56–7.43 (m, Ar*H*, 8H), 7.42–7.34 (m, Ar*H*, 3H), 6.96 (dd, *J*_H–P_ = 21.2 Hz, ^3^ *J*_H–H_ = 16.8 Hz, vinylic C*H*, 1H). ^13^C­{^1^H} NMR (101 MHz, CDCl_3_, δ): 147.9 (d, ^2^ *J*_C–P_ = 6.2 Hz, PCH=*C*H), 135.0 (d, ^3^ *J*_C–P_ = 19.7 Hz, PCH = CH*C*), 133.4 (d, ^1^ *J*_C–P_ = 87.2 Hz, *C*_ipso_), 131.54 (d, *J*_C–P_ = 3.3 Hz), 131.50 (d, *J*_C–P_ = 10.9 Hz), 130.1, 128.9, 128.7 (d, *J*_C–P_ = 12.4 Hz), 128.0, 119.7 (d, ^1^ *J*_C–P_ = 86.8 Hz, *C*_ipso_). ^31^P­{^1^H} NMR (162 MHz, CDCl_3_, δ): 37.2. Selected ATR-IR data (solid, cm^–1^): 3075, 3057, 3029, 3003, 1605, 1575, 1485, 1448, 1437, 1434, 1330, 1306, 1226, 1191, 1109, 1101, 998, 987, 850, 845, 806, 755, 739, 717, 708, 687. The NMR and IR data match those reported in the literature.^4^ X-ray quality crystals were obtained from slow evaporation of a hexanes/ethyl acetate (99:1) solution. The crystal structure has been published as a CSD Communication,^37^ and a similar structure has been reported by Rajaraman, Shanmugam, and co-workers.^35^

### X-ray Structure Determinations

Crystals of (Me_3_P)_2_Co­(μ-PPh_2_)_2_Co­(PMe_3_)_2_ (black tablet-shaped) were obtained from an NMR sample of “Co­(PMe_3_)_4_” mixed with HPPh_2_ in C_6_D_6_. Crystals of (Ph_2_PS_2_)_4_Co_2_ (dark green block-shaped) were obtained from an NMR sample in CDCl_3_ following the HCo­(HPPh_2_)_4_-catalyzed hydrophosphination reaction and the treatment of the reaction mixture with elemental sulfur. Intensity data were collected at 150 K on a Bruker D8 Venture Mo–IμS Photon-III diffractometer, λ = 0.71073 Å. Data collection frames were measured in shutterless mode. The data frames were processed using the program SAINT. The data were corrected for decay, Lorentz, and polarization effects as well as absorption and beam corrections. The structure was solved by a combination of direct methods and the difference Fourier technique as implemented in the SHELX suite of programs and refined by full-matrix least-squares on F^2^ for reflections out to 0.75Å resolution. Non-hydrogen atoms were refined with anisotropic displacement parameters. The isotropic displacement parameters were defined as a*U_eq_ (*a* = 1.5 for methyl, 1.2 for all others) of the adjacent atom. Hydrogen atoms were calculated and treated with a riding model, and (Me_3_P)_2_Co­(μ-PPh_2_)_2_Co­(PMe_3_)_2_ crystallized as two independent molecules. Neither compound cocrystallized with the solvent molecules. The crystal structures were deposited at the Cambridge Crystallographic Data Centre (CCDC) and allocated the deposition numbers CCDC 2527080 [for (Me_3_P)_2_Co­(μ-PPh_2_)_2_Co­(PMe_3_)_2_] and CCDC 2527081 [for (Ph_2_PS_2_)_4_Co_2_]. Crystal data collection and refinement parameters are listed in the Supporting Information.

## Supplementary Material



## Notes

Complete experimental details and characterization data (PDF)

## References

[1] Rosenberg L. (2013). Mechanisms of Metal-Catalyzed Hydrophosphination of
Alkenes and Alkynes. ACS Catal..

[2] Alonso F., Moglie Y., Radivoy G., Yus M. (2012). Solvent- and
Catalyst-Free Regioselective Hydrophosphanation of Alkenes. Green Chem..

[3] Li Y., Krause J. A., Guan H. (2020). Silane Activation with
Cobalt on the POCOP Pincer Ligand Platform. Organometallics.

[4] Ohmiya H., Yorimitsu H., Oshima K. (2005). Cobalt-Catalyzed *syn* Hydrophosphination of Alkynes. Angew. Chem.,
Int. Ed..

[5] Rajpurohit J., Kumar P., Shukla P., Shanmugam M., Shanmugam M. (2018). Mechanistic Investigation of Well-Defined Cobalt Catalyzed
Formal *E*-Selective Hydrophosphination of Alkynes. Organometallics.

[6] a Poli, R. Paramagnetic Mono- and Polyhydrides of the Transition Metals. In Recent Advances in Hydride Chemistry; Peruzzini, M. ; Poli, R. , Eds.; Elsevier: Amsterdam, 2001; pp 139–188.

[7] Klein H.-F., Karsch H. H. (1975). Methylkobaltverbindungen mit nicht
chelatisierenden
Liganden, I. Methyltetrakis­(trimethylphosphin)­kobalt und seine Derivate. Chem. Ber..

[8] Wu S., Li X., Xiong Z., Xu W., Lu Y., Sun H. (2013). Synthesis and Reactivity of Silyl
Iron, Cobalt, and
Nickel Complexes Bearing a [PSiP]-Pincer Ligand via Si–H Bond
Activation. Organometallics.

[9] Klein H.-F. (1971). Tetrakis­(trimethylphosphane)­cobalt­(0):
Preparation and Reactions. Angew. Chem., Int.
Ed..

[10] Ventre S., Simon C., Rekhroukh F., Malacria M., Amatore M., Aubert C., Petit M. (2013). Catalytic
Version of Enediyne Cobalt-Mediated
Cycloaddition and Selective Access to Unusual Bicyclic Trienes. Chem. – Eur. J..

[11] Hammer R., Klein H.-F. (1977). The Tetrakis­(trimethylphosphine)cobalt Anion. Z. Naturforsch..

[12] Suslick B. A., Tilley T. D. (2020). Mechanistic Interrogation of Alkyne
Hydroarylations Catalyzed by Highly Reduced, Single-Component Cobalt
Complexes. J. Am. Chem. Soc..

[13] Based on ^1^H NMR integration, we estimated that our best “Co(PMe_3_)_4_” sample had ∼15% HCo(PMe_3_)_4_, although the relative integration value for a diamagnetic impurity vs a paramagnetic species with resonances separated by >30 ppm is not particularly accurate.

[14] Misono A., Uchida Y., Saito T., Hidai M., Araki M. (1969). The Ligand
Effects on the N≡N Bond in the Nitrogen Complexes of Cobalt. Inorg. Chem..

[15] Both PPh_3_ and Ph_2_P-PPh_2_ were detected from the reaction mixture; however, their conversions from HPPh_2_ were too low (each < 2%) to be considered the main reaction pathways.

[16] Beck R., Klein H.-F. (2013). Bis­(*μ*-diphenylphosphanyl)­bis­[(trimethylphosphane)­cobalt­(I)]­(Co–Co). Acta Crystallogr., Sect. E:Struct. Rep. Online.

[17] Kornev A. N., Sushev V. V., Panova Y. S., Belina N. V., Lukoyanova O. V., Fukin G. K., Ketkov S. Y., Abakumov G. A., Lönnecke P., Hey-Hawkins E. (2012). The Intramolecular Rearrangement of Phosphinohydrazides
[R′_2_P-NR-NR-M] → [RN=PR′_2_-NR-M]: General Rules and Exceptions. Transformations of Bulky
Phosphinohydrazines (R-NH-N­(PPh_2_)_2_, R = *t*Bu, Ph_2_P). Inorg. Chem..

[18] Dai C., Stringer G., Corrigan J. F., Taylor N. J., Marder T. B., Norman N. C. (1996). Synthesis and Molecular
Structure of the Paramagnetic Co­(II) Bis­(boryl) Complex [Co­(PMe_3_)_3_(Bcat)_2_] (cat = 1,2-O_2_C_6_H_4_). J. Organomet. Chem..

[19] Qi X., Zhao H., Sun H., Li X., Fuhr O., Fenske D. (2018). Synthesis and Catalytic Application of [PPP]-pincer
Iron, Nickel and Cobalt Complexes for the Hydrosilylation of Aldehydes
and Ketones. New J. Chem..

[20] There was another paramagnetic species detected at the beginning of the reaction (resonances observed at 11.76, 9.04, and 4.14 ppm), although it was short-lived. One possibility is Co^0^(PMe_3_)_3_(HPPh_2_), which could potentially form via a ligand substitution reaction.

[21] Xie S., Dong Y., Du X., Fan Q., Yang H., Li X., Sun H., Fuhr O., Fenske D. (2021). Solvent-Free Hydrosilylation
of Alkenes Catalyzed by Well-Defined Low-Valent Cobalt Catalysts. Organometallics.

[22] Yuan J., Zhu L., Zhang J., Li J., Cui C. (2017). Sequential Addition of Phosphine to Alkynes for the
Selective Synthesis of 1,2-Diphosphinoethanes under Catalysis. Well-Defined
NHC-Copper Phosphides vs in Situ CuCl_2_/NHC Catalyst. Organometallics.

[23] Ventre S., Derat E., Amatore M., Aubert C., Petit M. (2013). Hydrido-Cobalt
Catalyst as a Selective Tool for the Dimerisation of Arylacetylenes:
Scope and Theoretical Studies. Adv. Synth. Catal..

[24] Ma X., Yan X., Li S., Chen X., Chen S., Yu J. (2025). Catalyst-
and Solvent-Free, Atom- and Step-Economical Synthesis of Dithiophosphinates
by One-Pot Domino Introduction of Sulfur Atoms. Green Chem..

[25] Huang Y., Li Y., Leung P.-H., Hayashi T. (2014). Asymmetric Synthesis of *P*-Stereogenic
Diarylphosphinites by Palladium-Catalyzed Enantioselective
Addition of Diarylphosphines to Benzoquinones. J. Am. Chem. Soc..

[26] Sato Y., Nishimura M., Kawaguchi S., Nomoto A., Ogawa A. (2019). Reductive
Rearrangement of Tetraphenyldiphosphine Disulfide to Trigger the Bisthiophosphinylation
of Alkenes and Alkynes. Chem. – Eur.
J..

[27] Hayashi M., Matsuura T., Tanake I., Ohta H., Watanabe Y. (2013). Pd-Catalyzed
P–C Cross-Coupling Reactions for Versatile Triarylphosphine
Synthesis. Org. Lett..

[28] Liu J., Le Y., Wu Y., Wang G., Yao C., Yu J., Li Q. (2024). Dithiophosphinylation of Allenyl Acetate: Access to 1,2-Bis­(diphenylphosphino)­ethane-Type
Bidentate Ligands. Org. Lett..

[29] Annan T. A., Kumar R., Tuck D. G. (1991). Direct
Electrochemical Synthesis and Crystallographic Characterization of
Metal Diphenylphosphido and Diphenylthiophosphinato Compounds, and
Some Derivatives. J. Chem. Soc., Dalton Trans..

[30] Cavell R. G., Day E. D., Byers W., Watkins P. M. (1972). Metal Complexes
of Substituted Dithiophosphinic Acids. V. Complexes of Manganese,
Iron, and Cobalt. Inorg. Chem..

[31] Di Giuseppe A., De Luca R., Castarlenas R., Pérez-Torrente J. J., Crucianelli M., Oro L. A. (2016). Double Hydrophosphination of Alkynes Promoted by Rhodium:
the Key Role of an *N*-Heterocyclic Carbene Ligand. Chem. Commun..

[32] Wang D., Chen Q., Leng X., Deng L. (2018). Reactions of Low-Coordinate Cobalt(0)–N-Heterocyclic Carbene
Complexes with Primary Aryl Phosphines. Inorg.
Chem..

[33] Karsch H. H., Milewski-Mahrla B. (1981). A Triply Bridged
Dicobalt Complex with Odd Number of Electrons (Co_2_
^+^). Angew. Chem., Int. Ed..

[34] It is possible that HCo(PMe_3_)_4_ decomposes to form a paramagnetic species, which in turn dynamically interacts with the hydride.

[35] Kumar P., Sen A., Rajaraman G., Shanmugam M. (2022). An Unusual Mixed-Valence Cobalt Dimer
as a Catalyst for the *anti*-Markovnikov Hydrophosphination
of Alkynes. Inorg. Chem. Front..

[36] Fallon B. J., Garsi J.-B., Derat E., Amatore M., Aubert C., Petit M. (2015). Synthesis of 1,2-Dihydropyridines
Catalyzed by Well-Defined Low-Valent Cobalt Complexes: C–H
Activation Made Simple. ACS Catal..

[37] Jayawardhena, J. P. I. D. ; Krause, J. A. ; Guan, H. CCDC 2514980: Experimental Crystal Structure Determination. 2025 10.5517/ccdc.csd.cc2qf1dm.

