# Supplementary material for: A Revisit of Co(PMe3)4‑Catalyzed Hydrophosphination of Phenylacetylene with HPPh2
Source: Organometallics. 2026 Mar 23;45(7):876–84. doi: 10.1021/acs.organomet.6c00034 (PMC13081116; doi:10.1021/acs.organomet.6c00034)
Supplement: Supplementary file 1 [file om6c00034_si_001.pdf]

Supporting Information

A Revisit of $Co(PMe_3)_4$-Catalyzed Hydrophosphination of Phenylacetylene with $HPPh_2$

*J. P. I. Dulmini Jayawardhena, Jeanette A. Krause, and Hairong Guan**

Department of Chemistry, University of Cincinnati, Cincinnati, Ohio 45221-0172, United States

Table of Contents

**Figures S1-S2**	NMR spectra of "$Co(PMe_3)_4$"	S2-S3
**Figures S3-S4**	NMR analysis of the reaction of "$Co(PMe_3)_4$" with $HPPh_2$	S4-S5
**Figures S5-S6**	Ligand substitution reaction between $HCo(PMe_3)_4$ and $HPPh_2$	S6-S7
**Figures S7-S9**	Synthesis and characterization of $HCo(HPPh_2)_4$	S8-S10
**Figures S10-S11**	Ligand substitution reaction between $HCo(HPPh_2)_4$ and PMe_3	S11-S12
**Figures S12-S16**	Characterization data of $(Me_3P)_2Co(\mu\text{-}PPh_2)_2Co(PMe_3)_2$	S13-S17
**Figures S17-S29**	Catalytic hydrophosphination of phenylacetylene (characterization of the hydrophosphination products)	S18-S28
**Figures S30-S31**	Thermal stability of $HCo(PMe_3)_4$	S29-S30
**Figure S32**	Annotation and reanalysis of the NMR spectra provided by Shanmugam, Shanmugam, and co-workers	S31
**Tables S3-S8**	Summary of crystallographic data	S32-S38
	References	S39

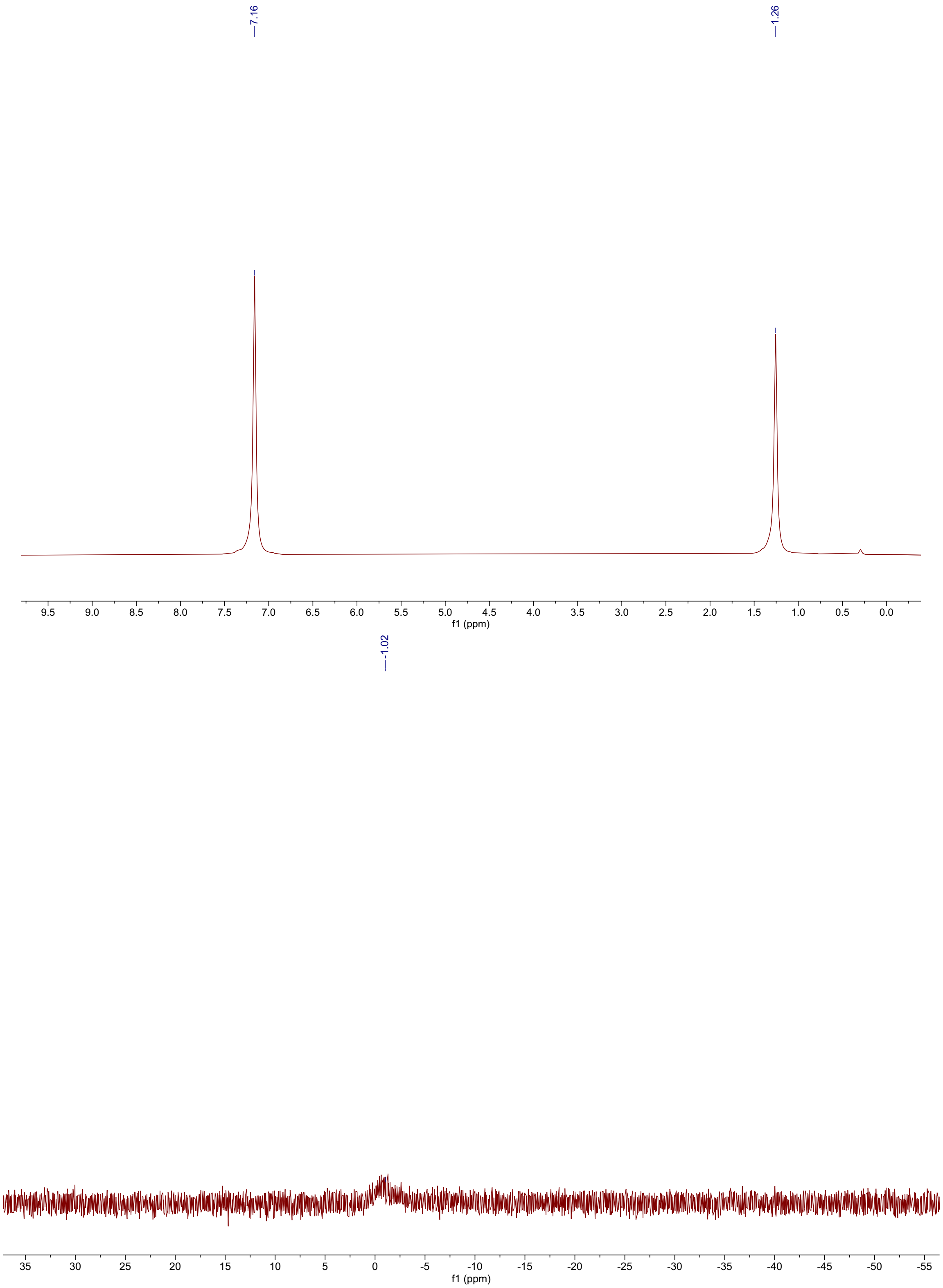

Figure S1. ^{1}H NMR (400 MHz, 23 °C, top) and $^{31}P\{^{1}H\}$ NMR (162 MHz, 23 °C, bottom) spectra of "$Co(PMe_3)_4$" (in C_6D_6) with a relatively narrow spectral window.

The NMR spectra resemble those shown by Shanmugam et al.,[1] cf. Figure S3 in the Supporting Information of that paper. The NMR data also match those reported by Zhang and co-workers.[2]

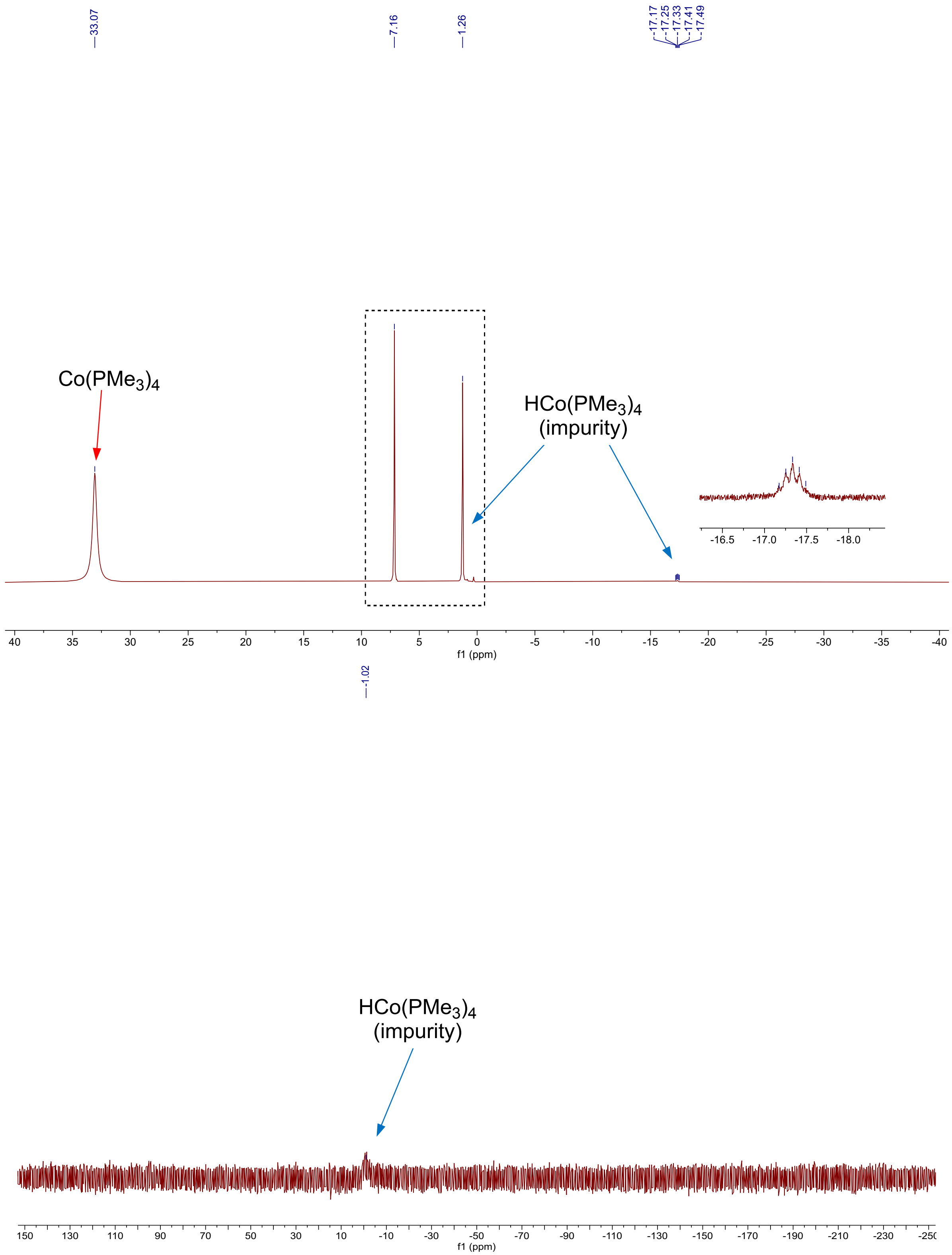

Figure S2. ^{1}H NMR (400 MHz, 23 °C, top) and ^{31}P{^{1}H} NMR (162 MHz, 23 °C, bottom) spectra of "$Co(PMe_3)_4$" (in C_6D_6) with an expanded spectral window. The boxed section is essentially what is shown in Figure S1. The NMR spectra shown here match those provided by Chirik et. al (see Figure S40 and Figure S41 of that paper).[3]

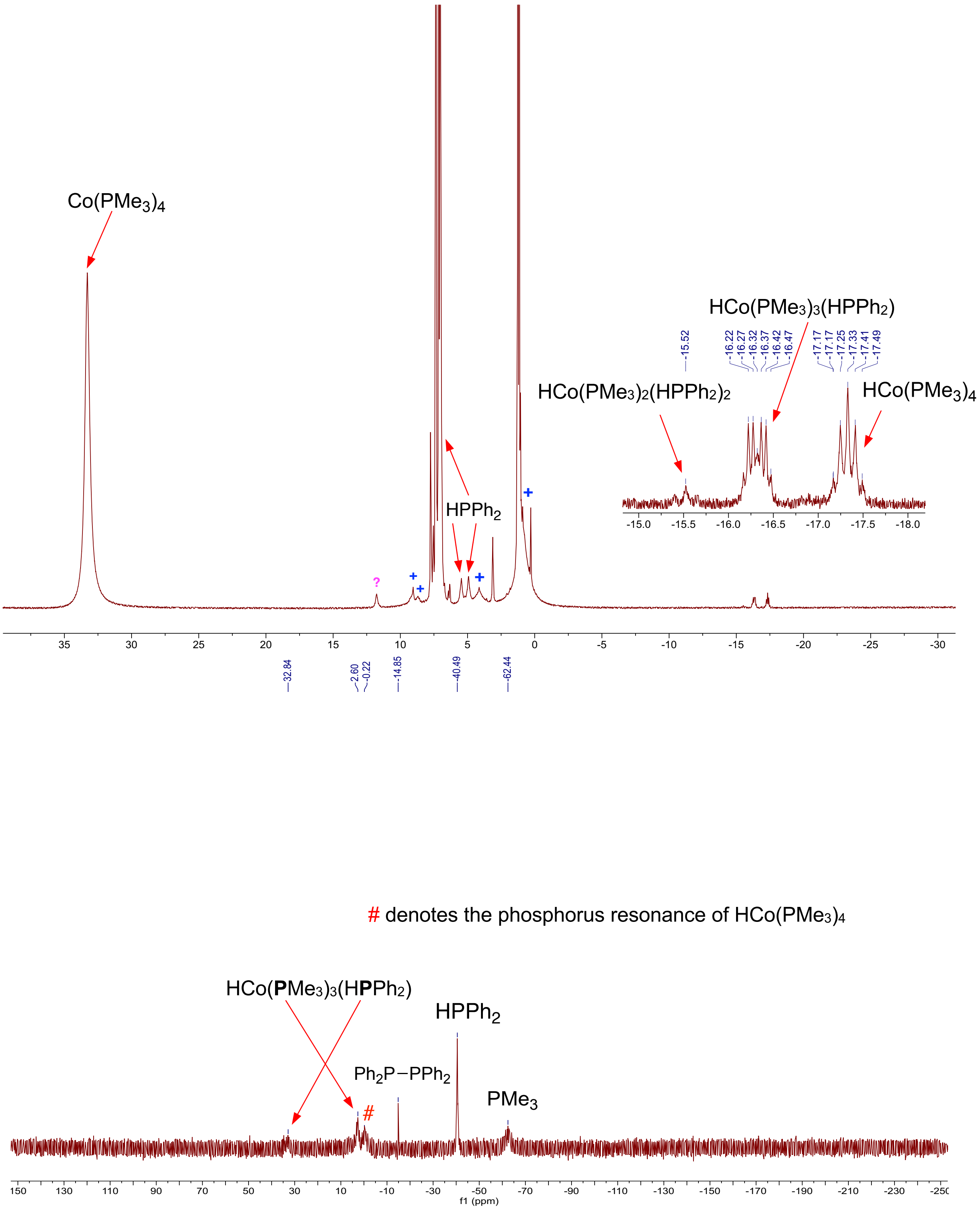

Figure S3. ^{1}H NMR (400 MHz, 23 °C, top) and $^{31}P\{^{1}H\}$ NMR (162 MHz, 23 °C, bottom) spectra of "$Co(PMe_3)_4$" (in C_6D_6, the same sample as the one shown in Figure S2) mixed with 1 equiv of $HPPh_2$. The NMR spectra were recorded within 10 min of mixing. The paramagnetic product is labelled with +. The resonance labelled with ? is a transient intermediate that disappeared quickly.

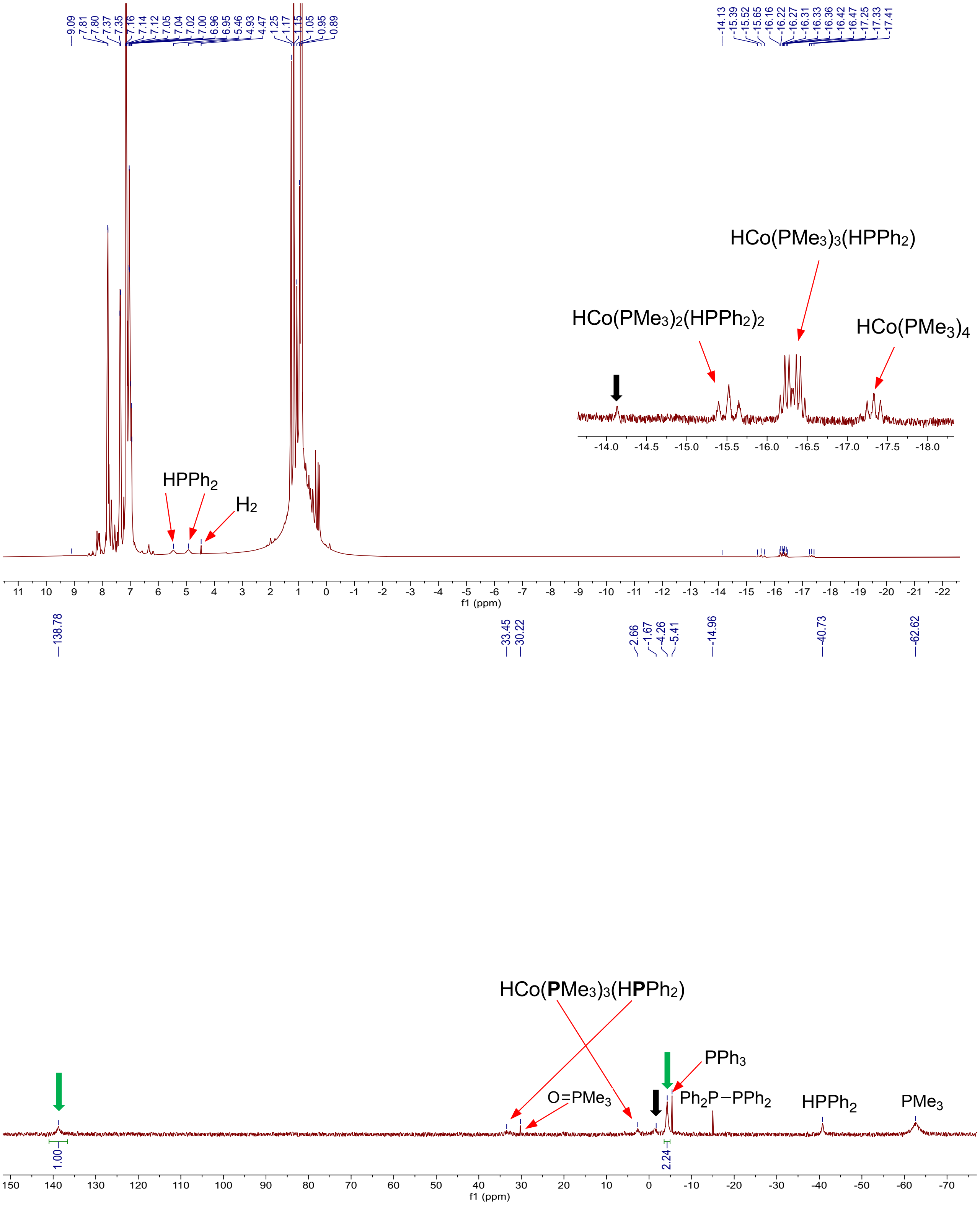

Figure S4. ^{1}H NMR (400 MHz, 23 °C, top) and ^{31}P{^{1}H} NMR (162 MHz, 23 °C, bottom) spectra of "$Co(PMe_3)_4$" (in C_6D_6, the same sample as the one shown in Figure S2) mixed with 1 equiv of $HPPh_2$ and then heated at 80 °C for 24 h. The green arrows are for resonances of $(Me_3P)_2Co(\mu\text{-}PPh_2)_2Co(PMe_3)_2$; the black arrow is for an unknown product.

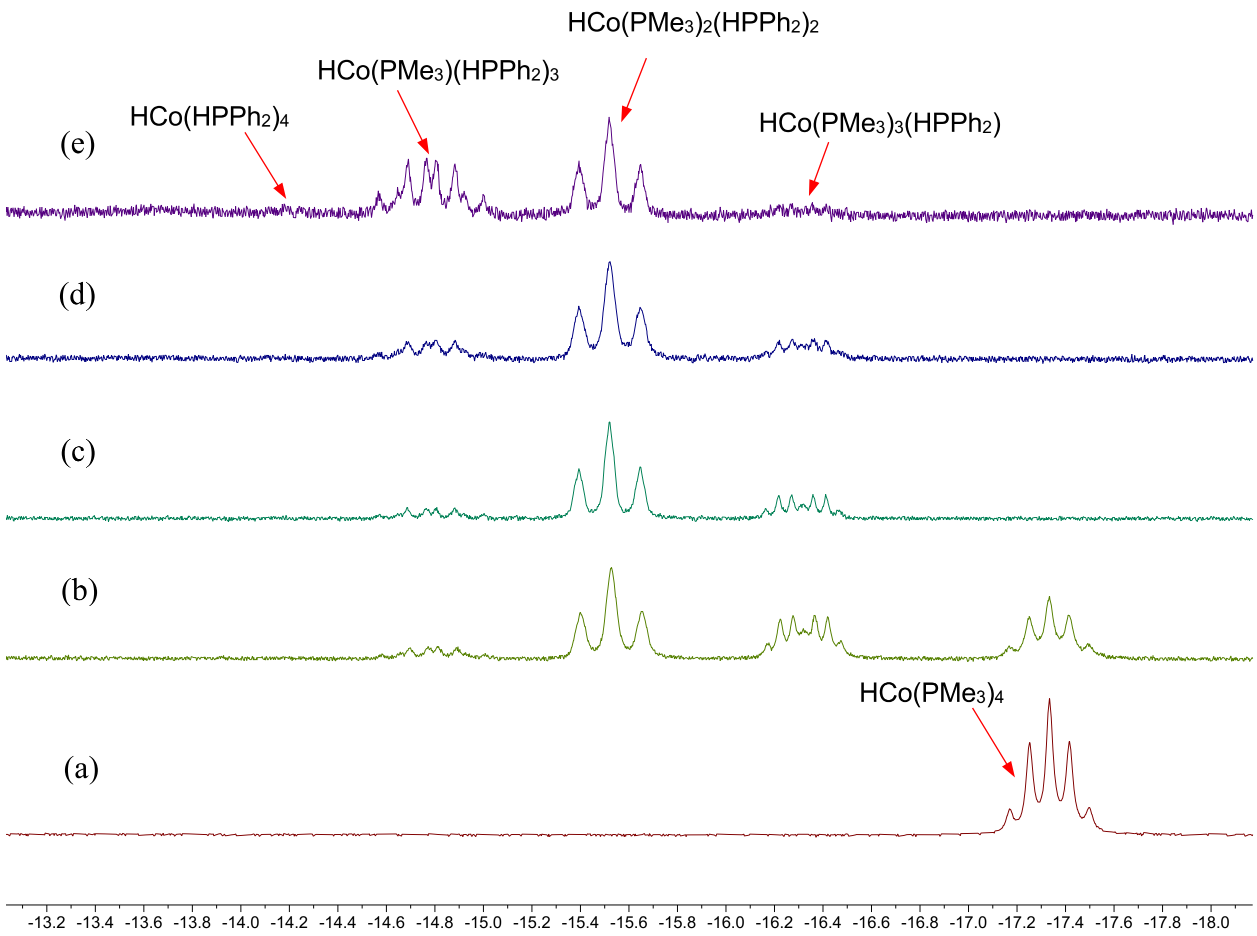

Figure S5. The hydride region of the ^{1}H NMR (400 MHz, in C_6D_6) spectra of
(a) $HCo(PMe_3)_4$
(b) $HCo(PMe_3)_4$ mixed with 10 equiv of $HPPh_2$ at 23 °C for 1 h
(c) $HCo(PMe_3)_4$ mixed with 10 equiv of $HPPh_2$ at 23 °C for 24 h
(d) $HCo(PMe_3)_4$ mixed with 10 equiv of $HPPh_2$ at 23 °C for 24 h followed by 80 °C for 1 h
(e) $HCo(PMe_3)_4$ mixed with 10 equiv of $HPPh_2$ at 23 °C for 24 h followed by 80 °C for 48 h

Table S1. Summary of the hydride resonances for $HCo(PMe_3)_{4-x}(HPPh_2)_x$ (x = 0-4)

cobalt hydride complex	δ_{CoH}
$HCo(PMe_3)_4$	–17.33 (quint, $J_{H\text{-}P}$ = 31.2 Hz)
$HCo(PMe_3)_3(HPPh_2)$	–16.32 (dq, $J_{H\text{-}P}$ = 57.6 and 21.6 Hz)
$HCo(PMe_3)_2(HPPh_2)_2$	–15.52 (tt, $J_{H\text{-}P}$ = 49.6 and 6.0 Hz)
$HCo(PMe_3)(HPPh_2)_3$	–14.79 (qd, $J_{H\text{-}P}$ = 44.6 and 31.4 Hz)
$HCo(HPPh_2)_4$	–14.18 (quint, $J_{H\text{-}P}$ = 25.2 Hz)

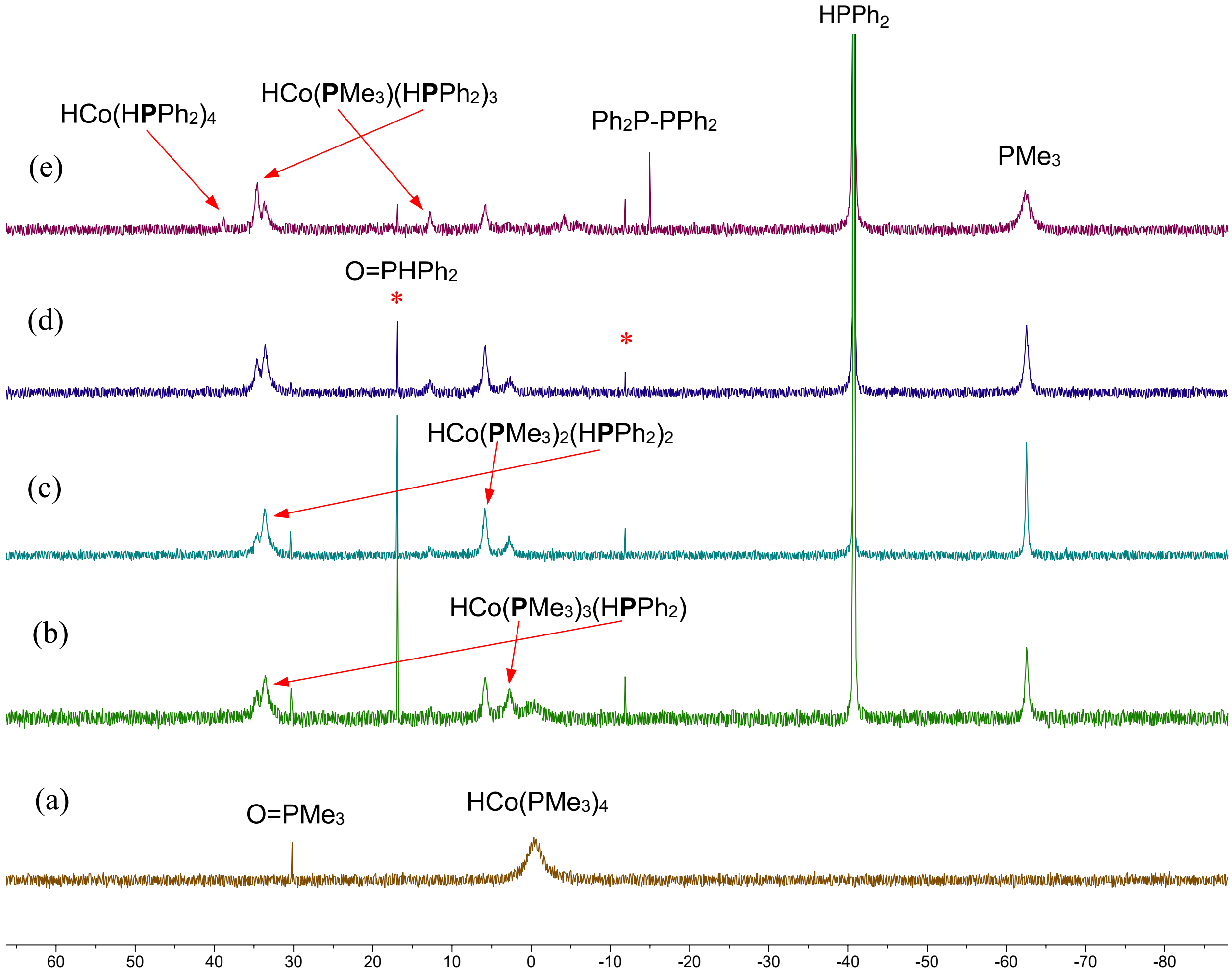

Figure S6. $^{31}P\{^{1}H\}$ NMR (162 MHz, in C_6D_6) spectra of
(a) $HCo(PMe_3)_4$ ($O{=}PMe_3$ was present as an impurity, <1%)
(b) $HCo(PMe_3)_4$ mixed with 10 equiv of $HPPh_2$ at 23 °C for 1 h
(c) $HCo(PMe_3)_4$ mixed with 10 equiv of $HPPh_2$ at 23 °C for 24 h
(d) $HCo(PMe_3)_4$ mixed with 10 equiv of $HPPh_2$ at 23 °C for 24 h followed by 80 °C for 1 h
(e) $HCo(PMe_3)_4$ mixed with 10 equiv of $HPPh_2$ at 23 °C for 24 h followed by 80 °C for 48 h
*denotes impurities present in the commercial sample of $HPPh_2$ (~1%)

Table S2. Summary of the phosphorus resonances for $HCo(PMe_3)_{4-x}(HPPh_2)_x$ (x = 0-4)

**cobalt hydride complex**	δ_P
$HCo(PMe_3)_4$	–0.5 (PMe_3)
$HCo(PMe_3)_3(HPPh_2)$	2.7 (PMe_3) and 32.8 ($HPPh_2$)
$HCo(PMe_3)_2(HPPh_2)_2$	5.8 (PMe_3) and 33.7 ($HPPh_2$)
$HCo(PMe_3)(HPPh_2)_3$	12.6 (PMe_3) and 34.6 ($HPPh_2$)
$HCo(HPPh_2)_4$	38.8 ($HPPh_2$)

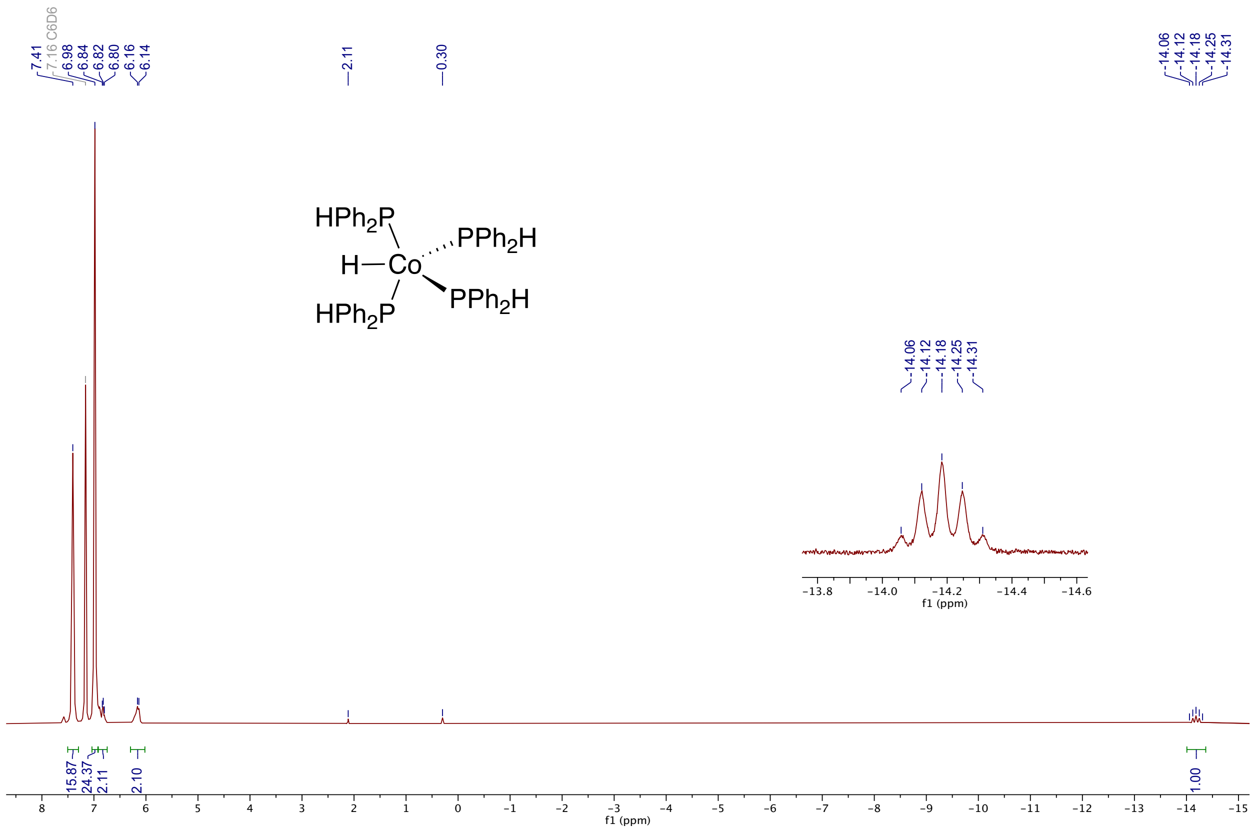

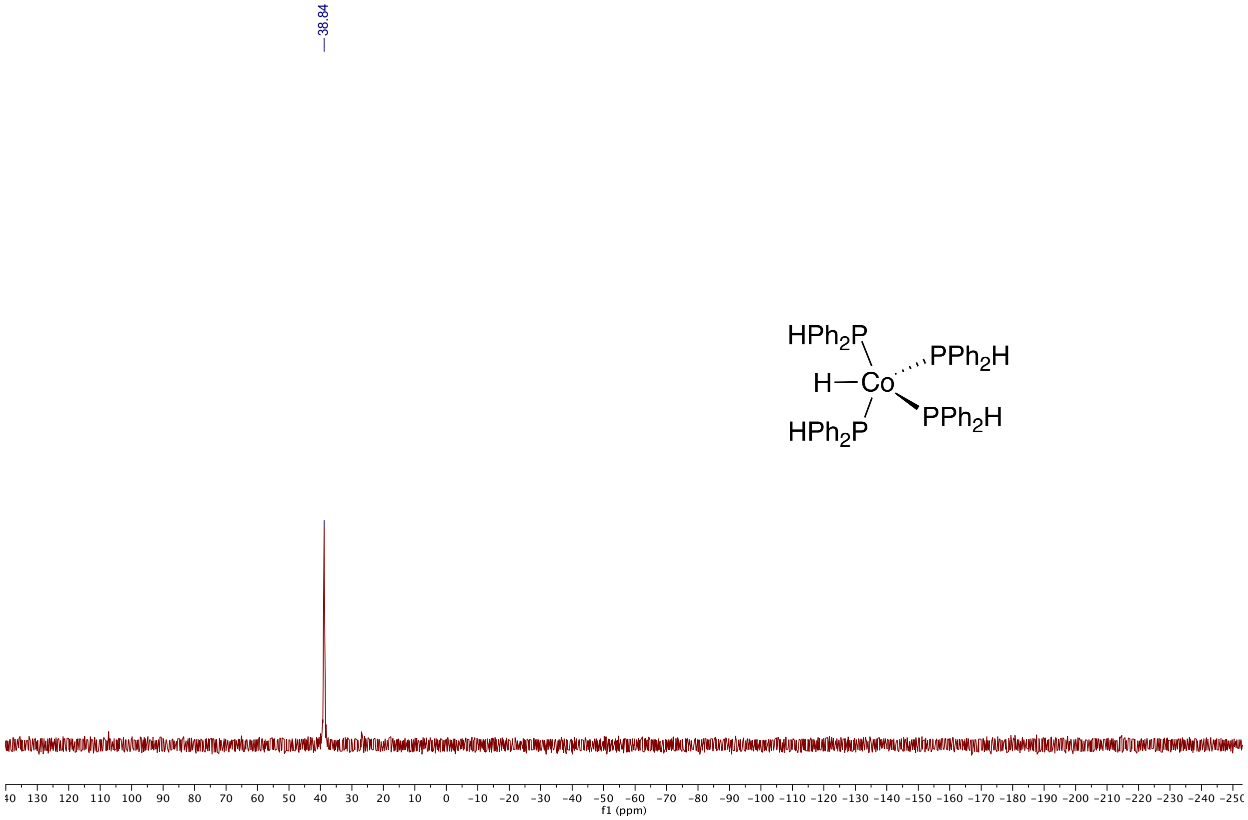

Figure S7. ^{1}H NMR (400 MHz, 23 °C, top) and ^{31}P{^{1}H} NMR (162 MHz, 23 °C, bottom) spectra of $HCo(HPPh_2)_4$ dissolved in C_6D_6

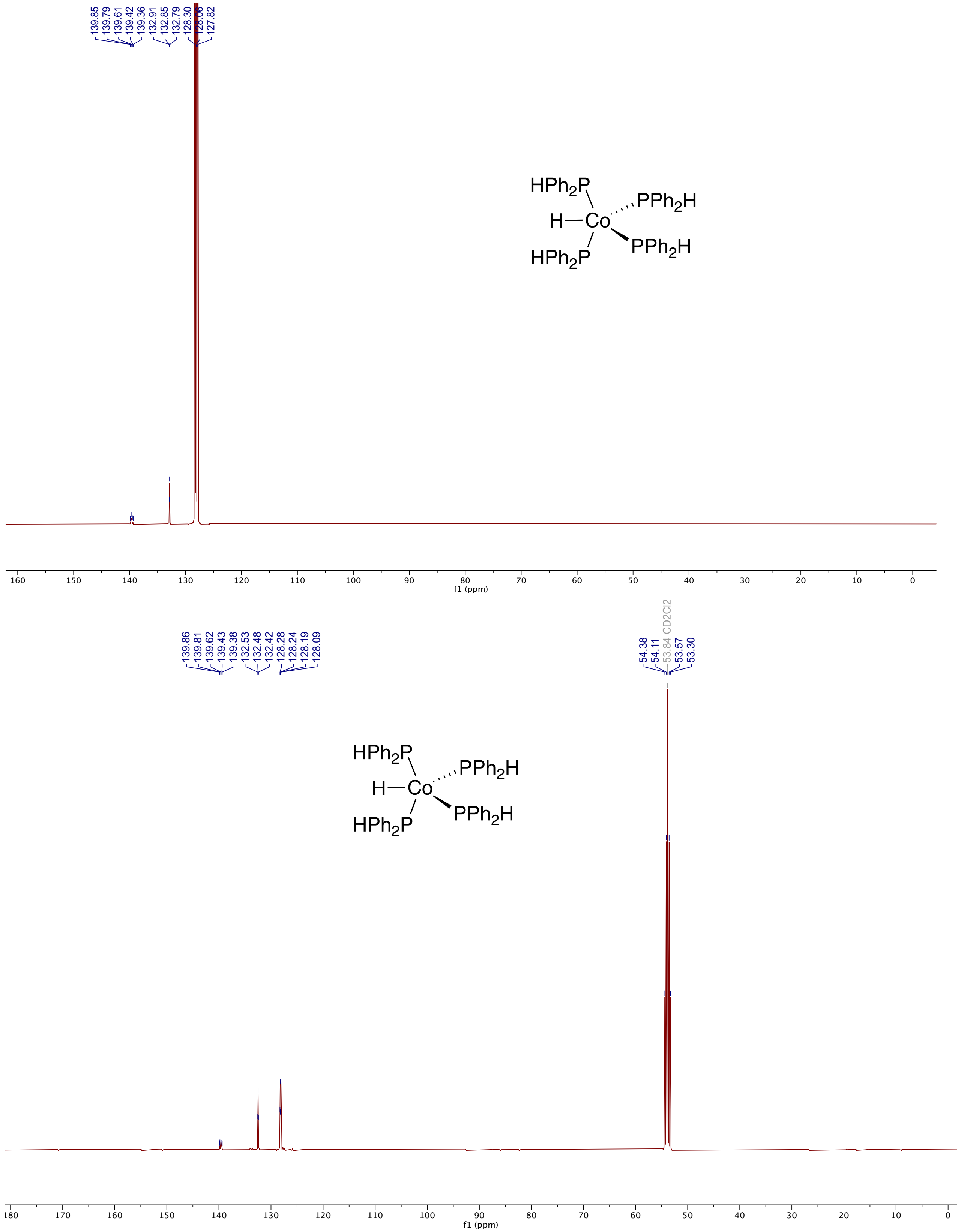

Figure S8. $^{13}C\{^{1}H\}$ NMR (101 MHz, 23 °C) spectra of $HCo(HPPh_2)_4$ dissolved in C_6D_6 (top) and CD_2Cl_2 (bottom)

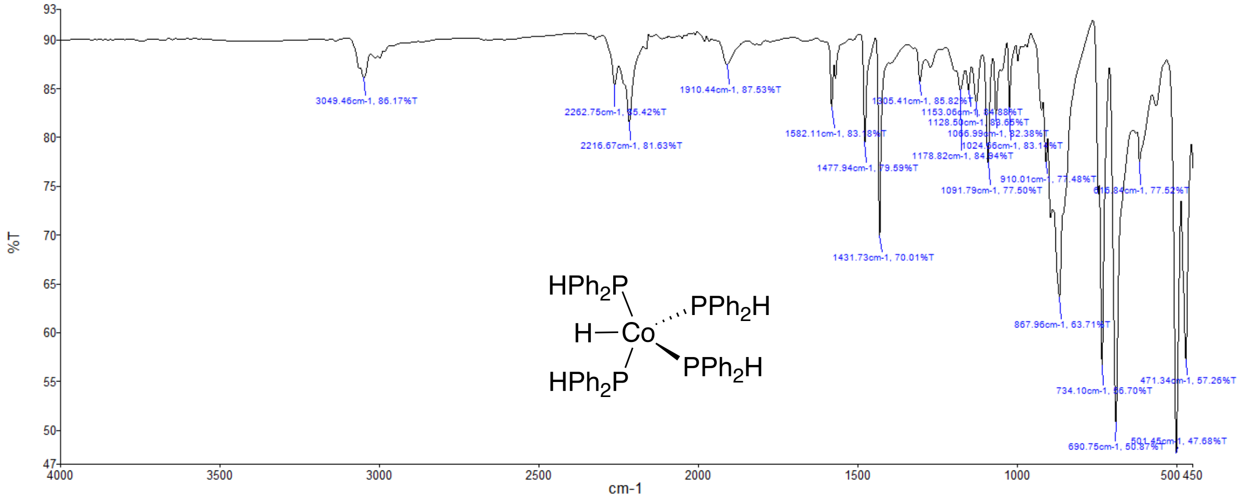

Figure S9. ATR-IR spectrum of $HCo(HPPh_2)_4$ (solid sample)

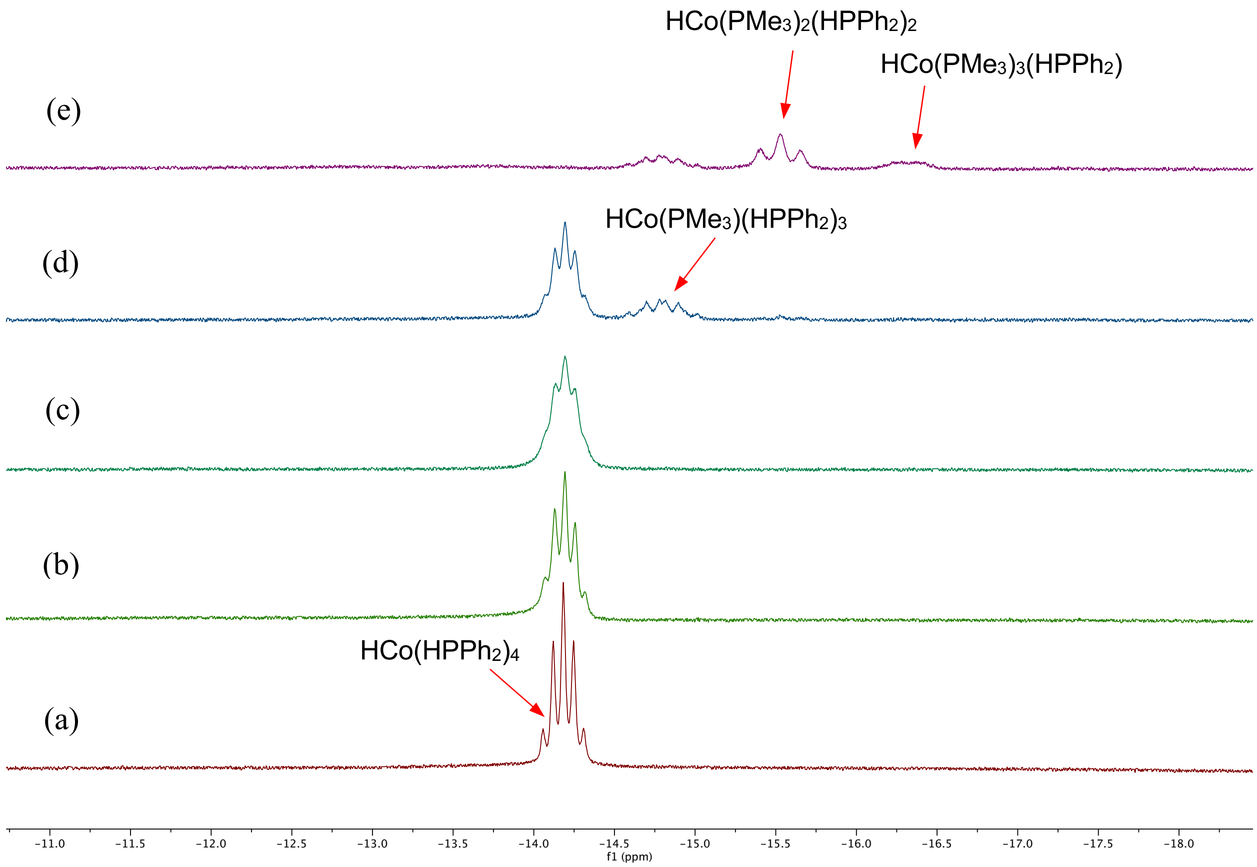

Figure S10. ^{1}H NMR (400 MHz, in C_6D_6) spectra of
(a) $HCo(HPPh_2)_4$
(b) $HCo(HPPh_2)_4$ mixed with 10 equiv of PMe_3 at 23 °C for 1 h
(c) $HCo(HPPh_2)_4$ mixed with 10 equiv of PMe_3 at 23 °C for 24 h
(d) $HCo(HPPh_2)_4$ mixed with 10 equiv of PMe_3 at 23 °C for 24 h followed by 80 ºC for 1 h
(e) $HCo(HPPh_2)_4$ mixed with 10 equiv of PMe_3 at 23 °C for 24 h followed by 80 ºC for 48 h

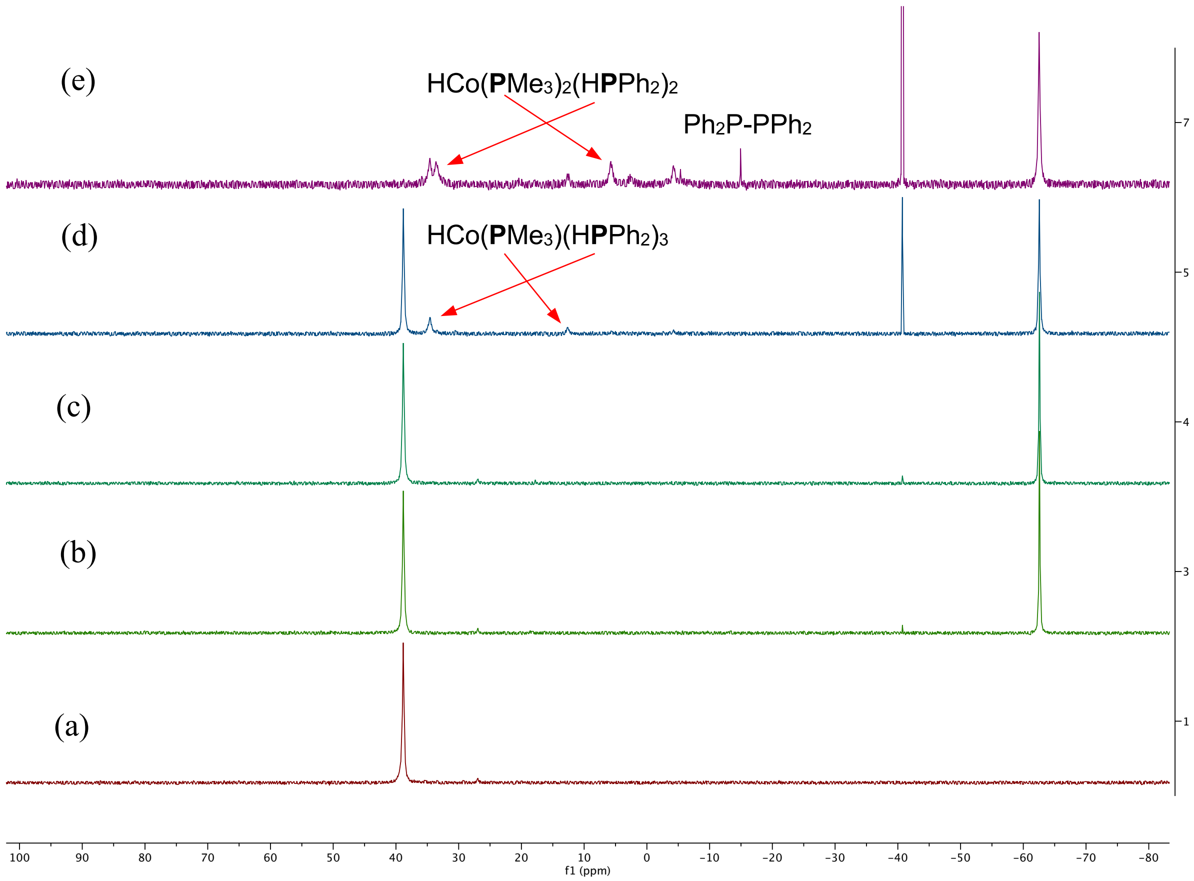

Figure S11. $^{31}P\{^{1}H\}$ NMR (162 MHz, in C_6D_6) spectra of
(a) $HCo(HPPh_2)_4$
(b) $HCo(HPPh_2)_4$ mixed with 10 equiv of PMe_3 at 23 °C for 1 h
(c) $HCo(HPPh_2)_4$ mixed with 10 equiv of PMe_3 at 23 °C for 24 h
(d) $HCo(HPPh_2)_4$ mixed with 10 equiv of PMe_3 at 23 °C for 24 h followed by 80 ºC for 1 h
(e) $HCo(HPPh_2)_4$ mixed with 10 equiv of PMe_3 at 23 °C for 24 h followed by 80 ºC for 48 h

Crystallographic and Spectroscopic Characterization of $(Me_3P)_2Co(\mu\text{-}PPh_2)_2Co(PMe_3)_2$

Dark tablet-shaped crystals formed from the NMR sample shown in Figure S4 (i.e., a 1 : 1 mixture of "$Co(PMe_3)_4$" and $HPPh_2$ in C_6D_6 heated at 80 °C for 24 h). This compound crystallized as two independent molecules in a triclinic crystal system (space group P-1). The same compound was prepared by Beck and Klein from the reaction of $MeCo(PMe_3)_4$ with 1 equiv of $HPPh_2$ in *n*-pentane and crystallized from *n*-pentane/diethyl ether in a monoclinic crystal system (space group $P2_1/c$).[4] Crystal data collection and refinement parameters for our sample are provide in Table S3-S5. ORTEP of $(Me_3P)_2Co(\mu\text{-}PPh_2)_2Co(PMe_3)_2$ is shown below.

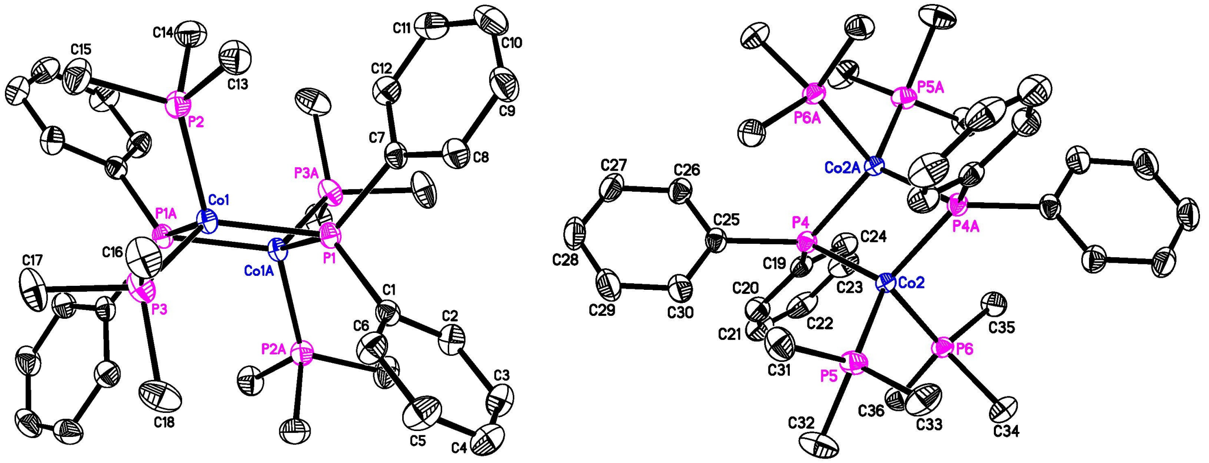

Figure S12. ORTEP of both independent molecules of $(Me_3P)_2Co(\mu\text{-}PPh_2)_2Co(PMe_3)_2$ at the 50% probability level (for clarity hydrogen atoms are omitted and only symmetry-unique carbon atoms are labeled).

The single crystals of $(Me_3P)_2Co(\mu\text{-}PPh_2)_2Co(PMe_3)_2$ were dissolved in C_6D_6 as well as in THF-d_8 for NMR analysis. The NMR spectra are provided in Figure S13 and S14. The signal-to-noise ratio in Figure S13 is low due to the fact that this dinuclear complex has a low solubility in C_6D_6.

^{1}H NMR (400 MHz, C_6D_6, δ): 7.83-7.79 (br, Ar*H*, 8H), 7.15-7.12 (m, Ar*H*, 12H), 0.89 (br, P(C*H*$_3$)$_3$, 36H). ^{31}P{^{1}H} NMR (162 MHz, C_6D_6, δ): 139.1 (br, *P*Ph$_2$, 2P), –4.4 (br, *P*Me$_3$, 4P).

^{1}H NMR (400 MHz, THF-d_8, δ): 7.57-7.53 (br, Ar*H*, 8H), 7.10-7.06 (m, Ar*H*, 12H), 0.89 (br, P(C*H*$_3$)$_3$, 36H). ^{31}P{^{1}H} NMR (162 MHz, THF-d_8, δ): 138.4 (br, *P*Ph$_2$, 2P), –4.3 (br, *P*Me$_3$, 4P).

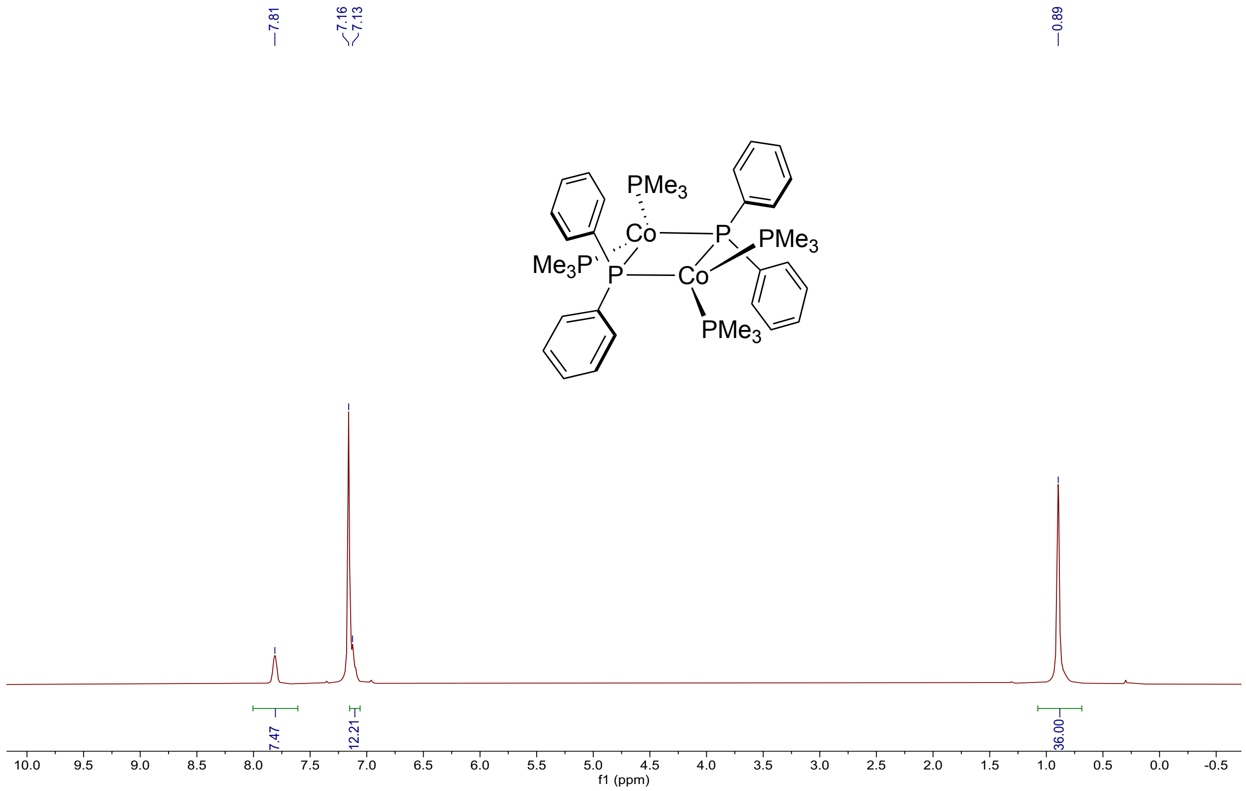

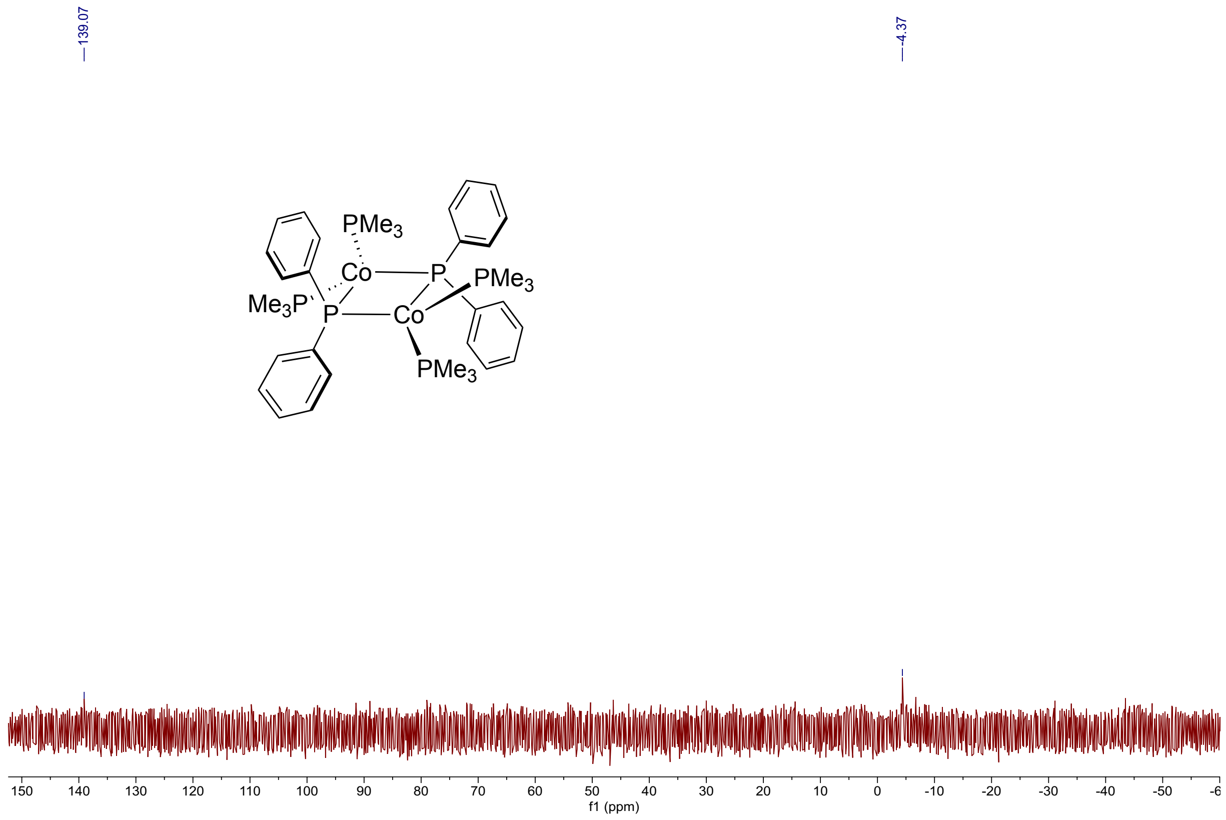

Figure S13. ^{1}H NMR (400 MHz, 23 °C, top) and ^{31}P{^{1}H} NMR (162 MHz, 23 °C, bottom) spectra of $(Me_3P)_2Co(\mu\text{-}PPh_2)_2Co(PMe_3)_2$ dissolved in C_6D_6

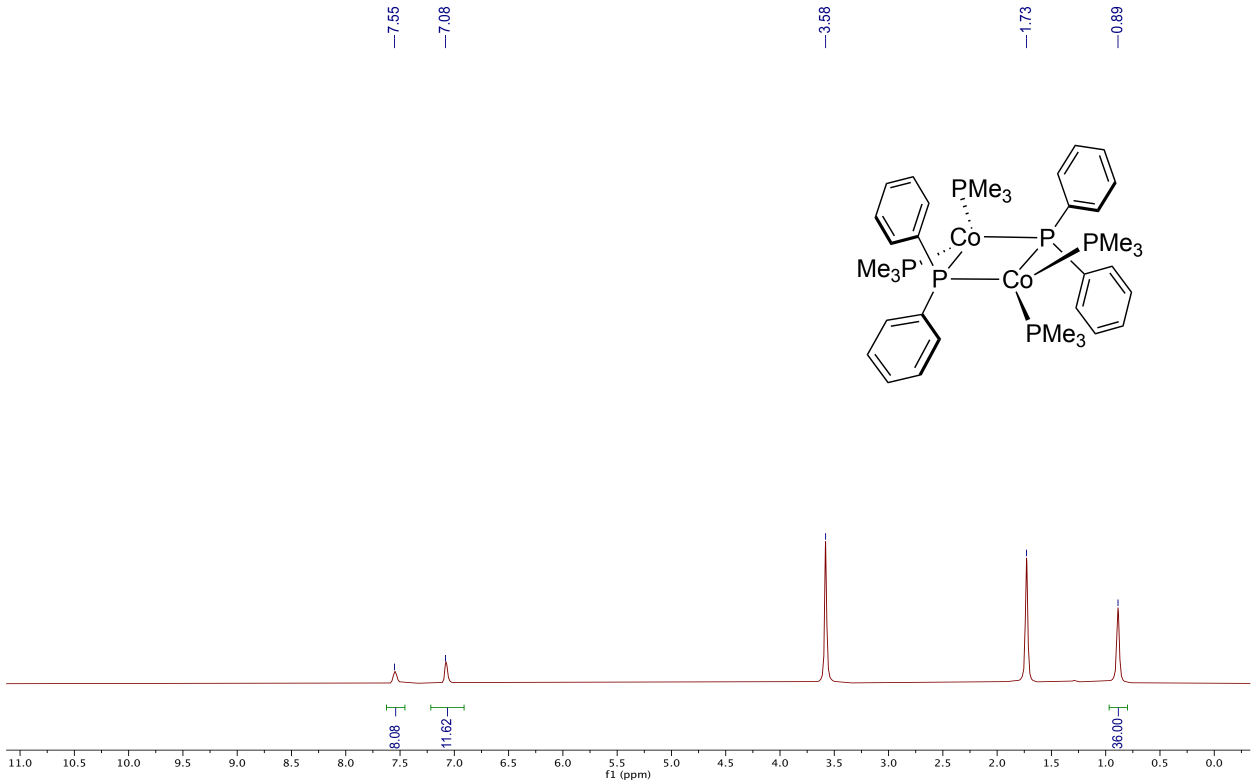

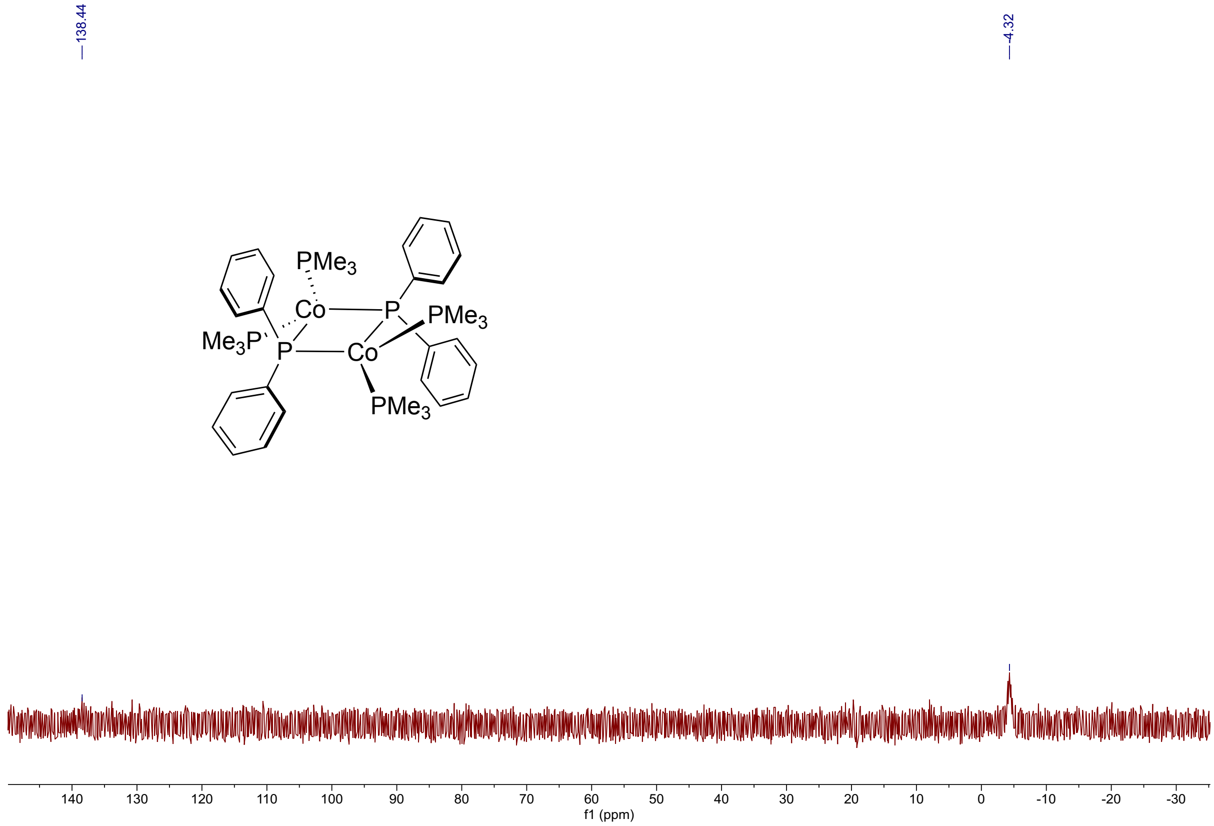

Figure S14. ^{1}H NMR (400 MHz, 23 °C, top) and $^{31}P\{^{1}H\}$ NMR (162 MHz, 23 °C, bottom) spectra of $(Me_3P)_2Co(\mu\text{-}PPh_2)_2Co(PMe_3)_2$ dissolved in THF-d_8

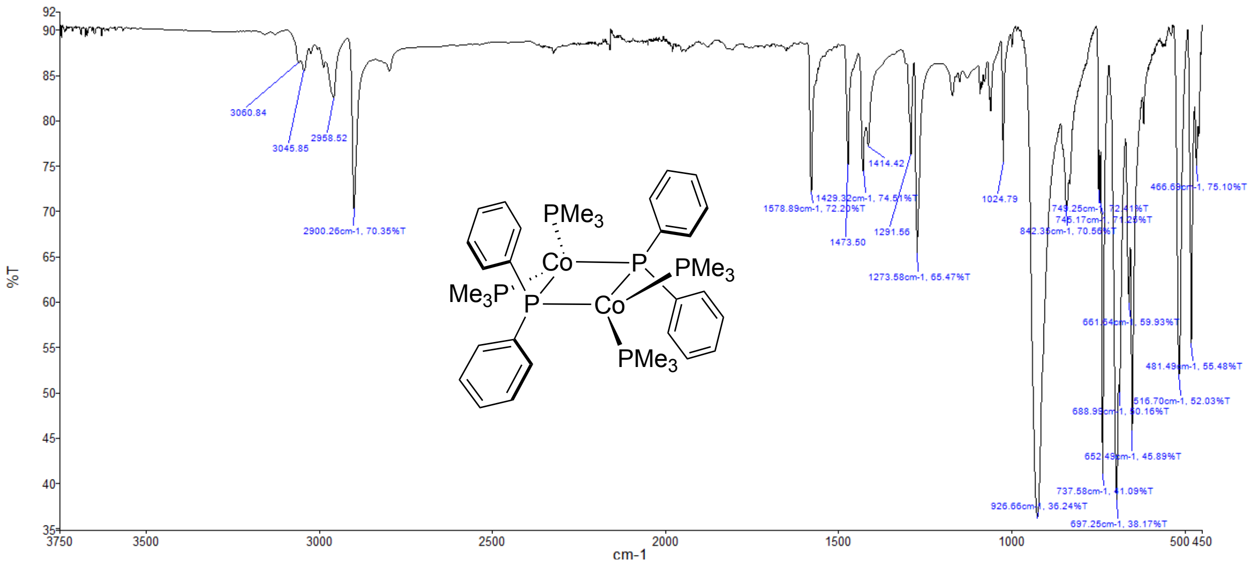

Figure S15. ATR-IR spectrum of $(Me_3P)_2Co(\mu\text{-}PPh_2)_2Co(PMe_3)_2$ (solid sample)

The NMR scale reaction of "Co(PMe3)4" with 1 equiv of HPPh2 was carried out in THF-d_8. The $^{31}P\{^1H\}$ NMR spectra are summarized below.

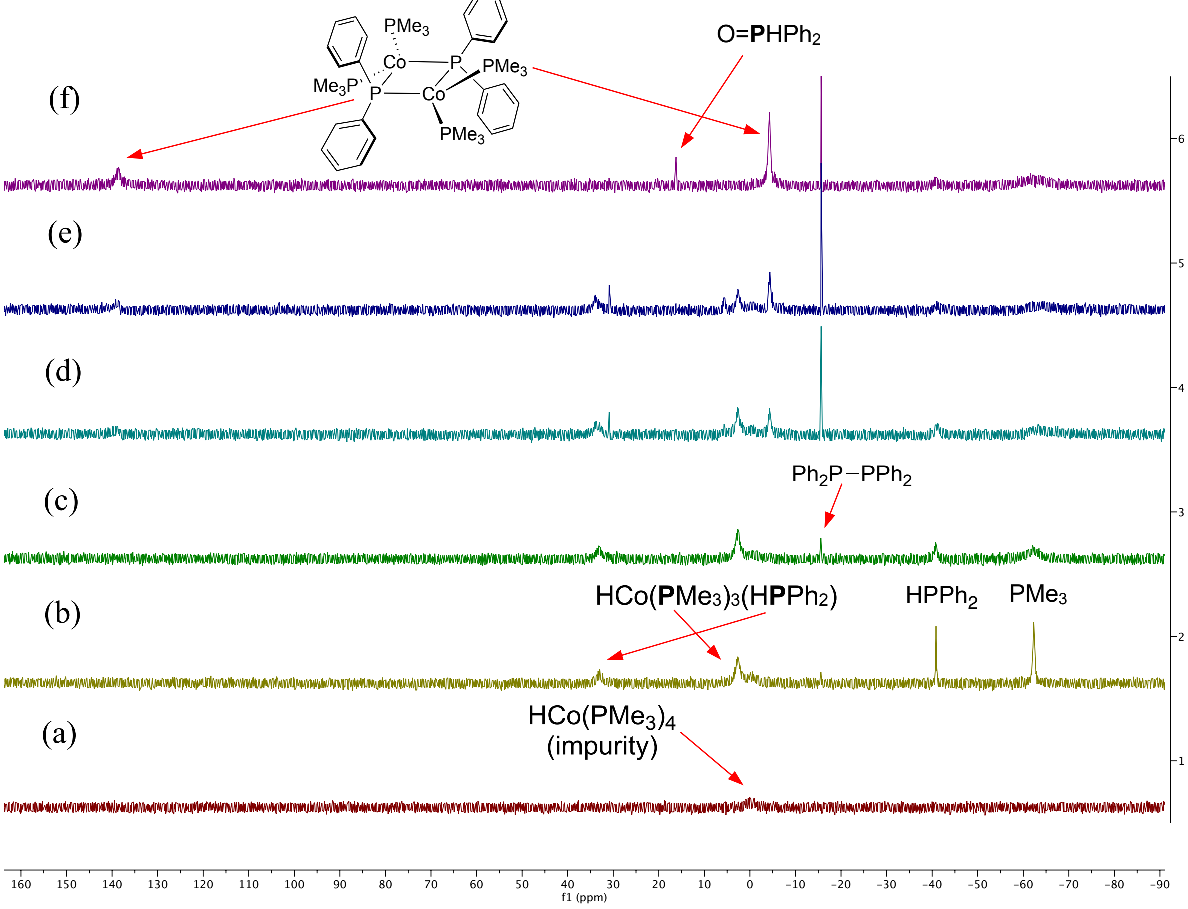

Figure S16. $^{31}P\{^1H\}$ NMR (162 MHz, in THF-d_8) spectra of
(a) "Co(PMe3)4"
(b) "Co(PMe3)4" mixed with 1 equiv of HPPh2 at 23 °C (right after mixing)
(c) "Co(PMe3)4" mixed with 1 equiv of HPPh2 at 23 °C for 2 h
(d) "Co(PMe3)4" mixed with 1 equiv of HPPh2 at 23 °C for 24 h
(e) "Co(PMe3)4" mixed with 1 equiv of HPPh2 at 23 °C for 24 h followed by 80 °C for 2 h
(f) "Co(PMe3)4" mixed with 1 equiv of HPPh2 at 23 °C for 24 h followed by 80 °C for 24 h

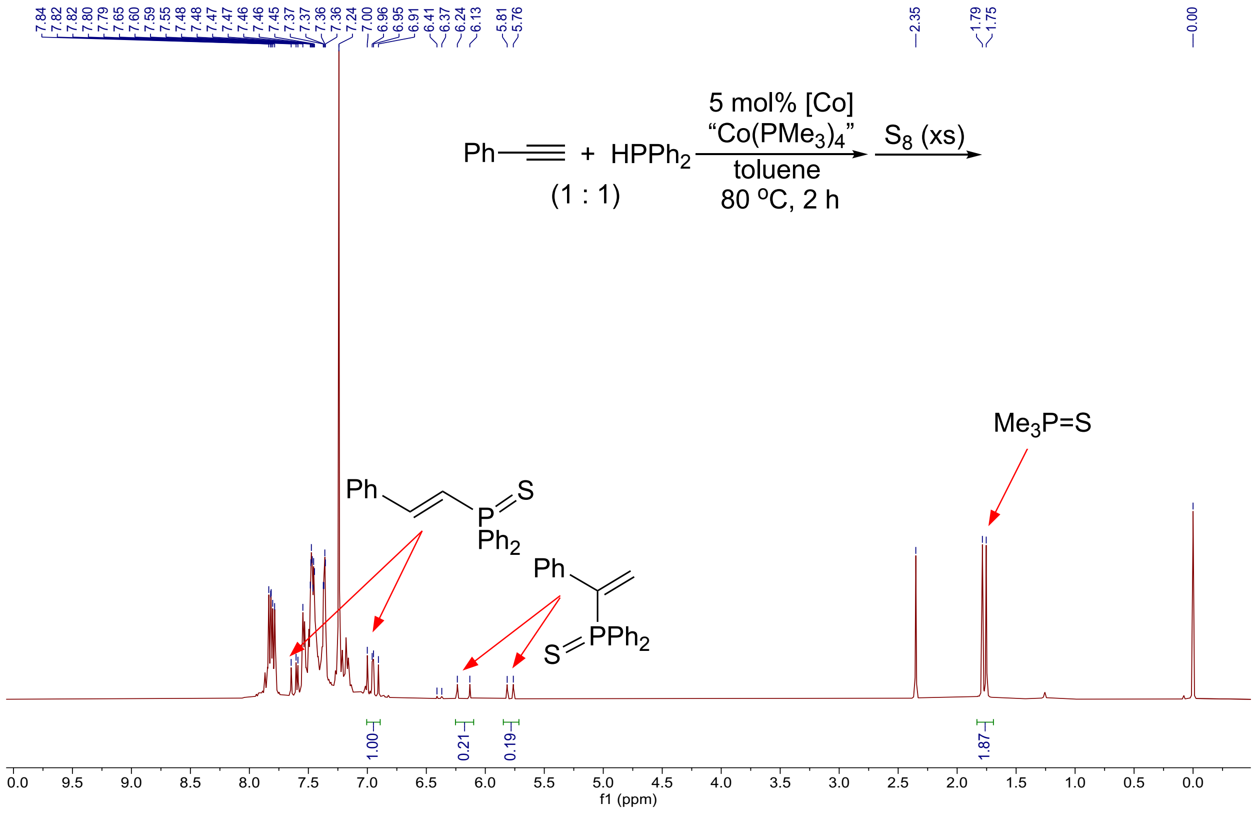

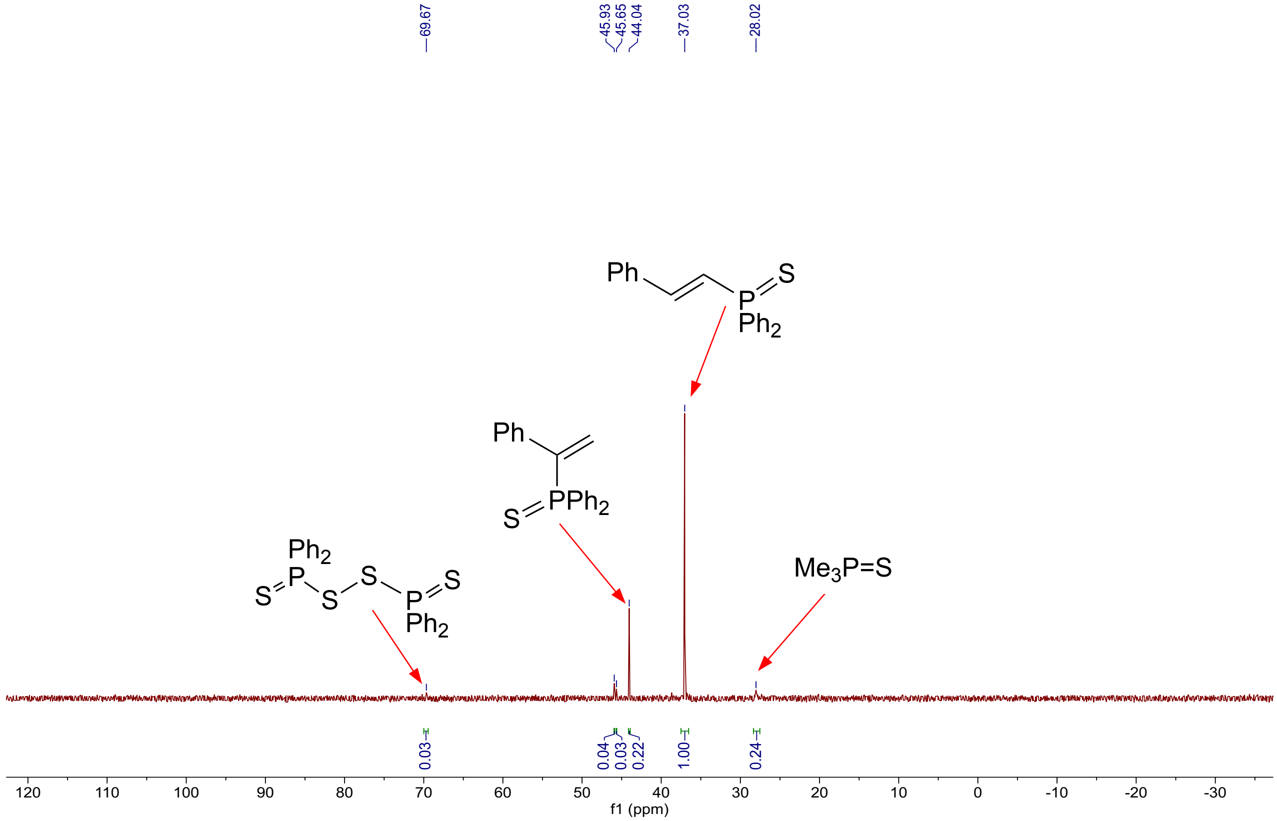

Figure S17. ^{1}H NMR (400 MHz, 23 °C, top) and $^{31}P\{^{1}H\}$ NMR (162 MHz, 23 °C, bottom) spectra of the crude products dissolved in $CDCl_3$ (for the reaction catalyzed by "$Co(PMe_3)_4$")

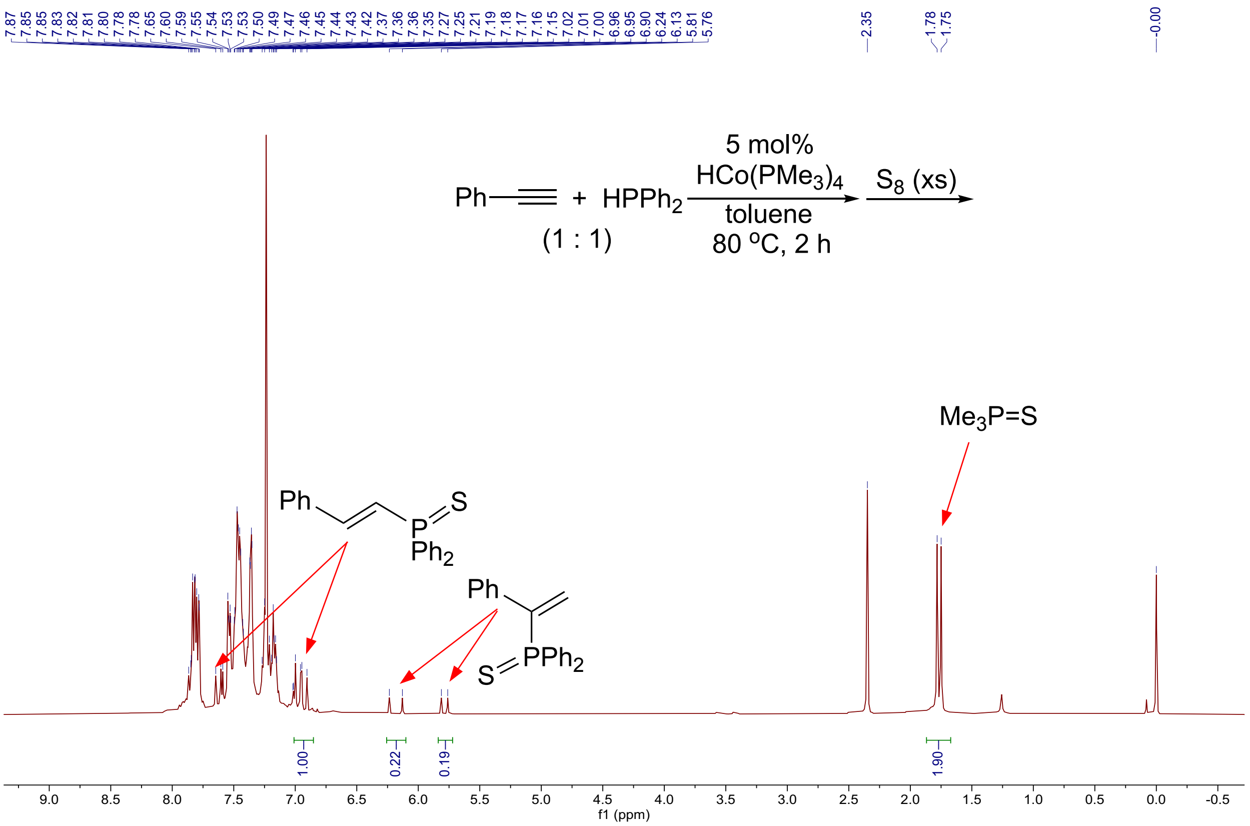

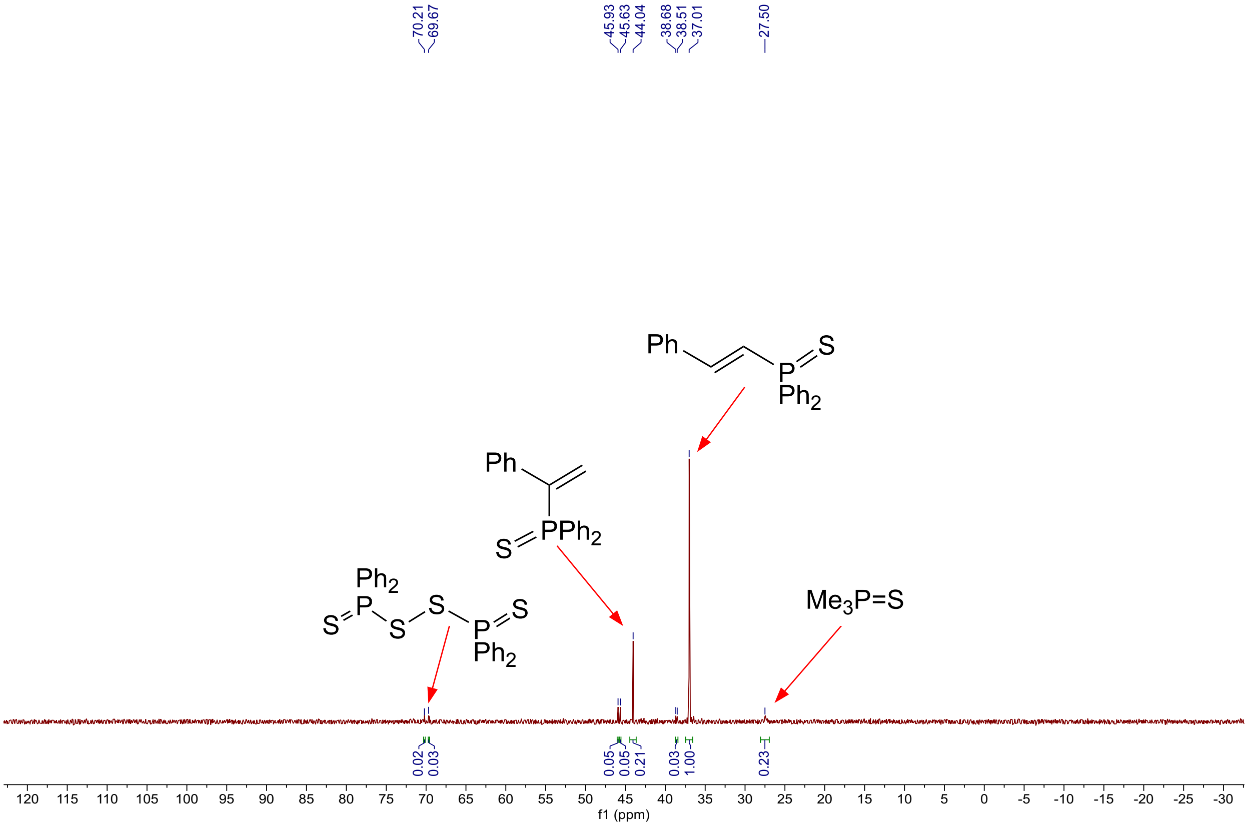

Figure S18. ^{1}H NMR (400 MHz, 23 °C, top) and $^{31}P\{^{1}H\}$ NMR (162 MHz, 23 °C, bottom) spectra of the crude products dissolved in $CDCl_3$ (for the reaction catalyzed by $HCo(PMe_3)_4$)

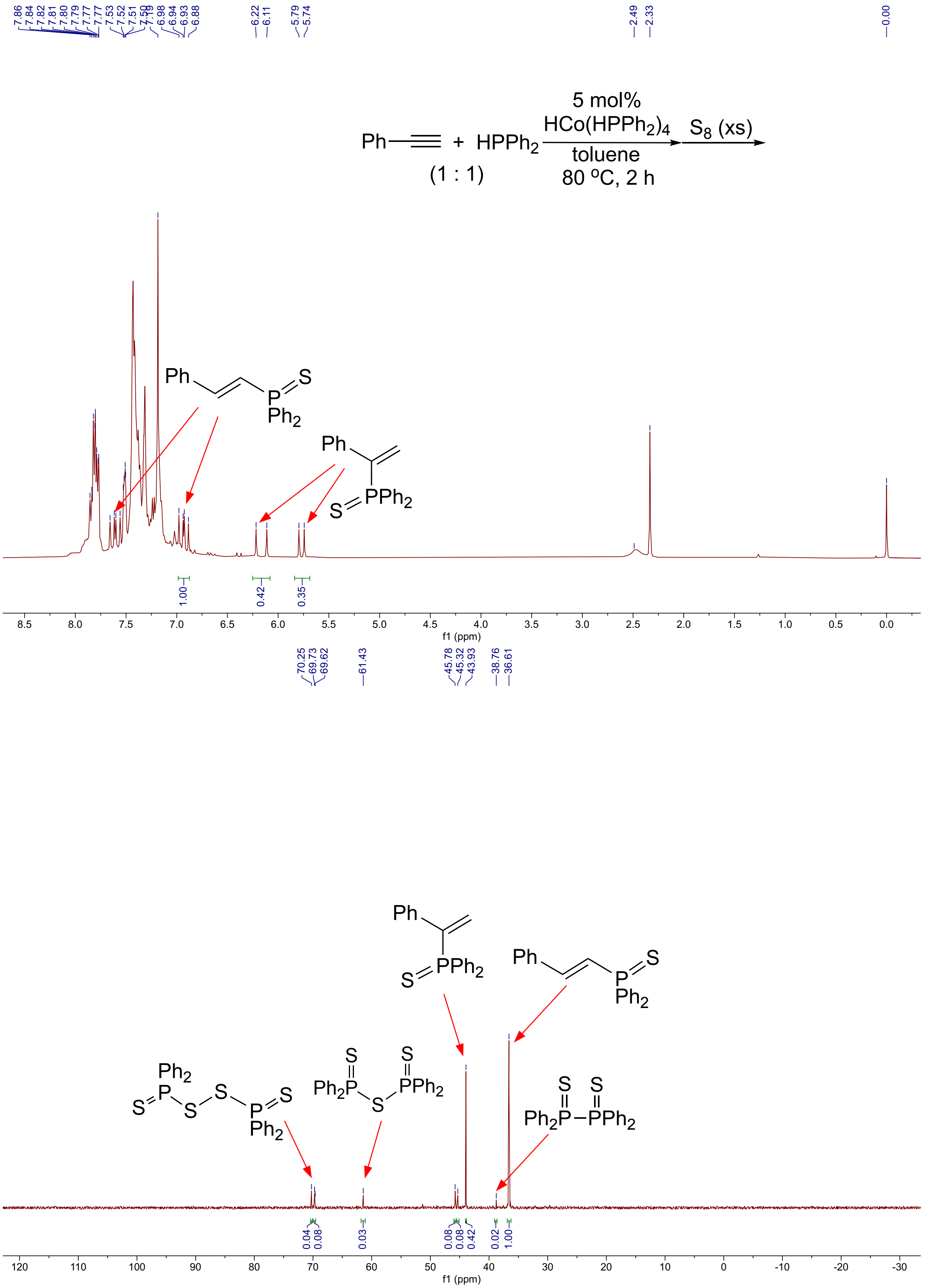

Figure S19. ^{1}H NMR (400 MHz, 23 °C, top) and $^{31}P\{^{1}H\}$ NMR (162 MHz, 23 °C, bottom) spectra of the crude products dissolved in $CDCl_3$ (for the reaction catalyzed by $HCo(HPPh_2)_4$)

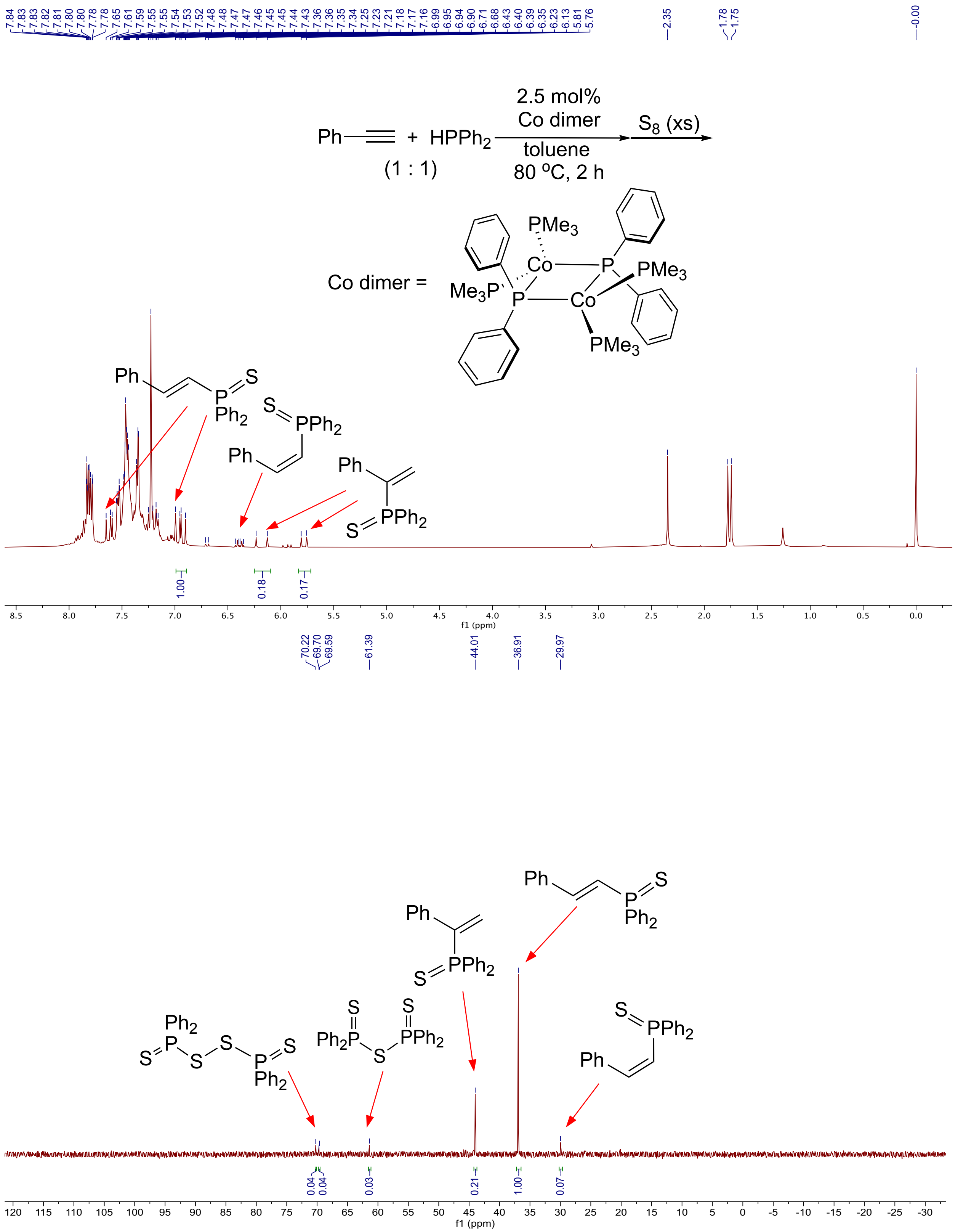

Figure S20. ^{1}H NMR (400 MHz, 23 °C, top) and $^{31}P\{^{1}H\}$ NMR (162 MHz, 23 °C, bottom) spectra of the crude products dissolved in $CDCl_3$ (for the reaction catalyzed by $(Me_3P)_2Co(\mu\text{-}PPh_2)_2Co(PMe_3)_2$)

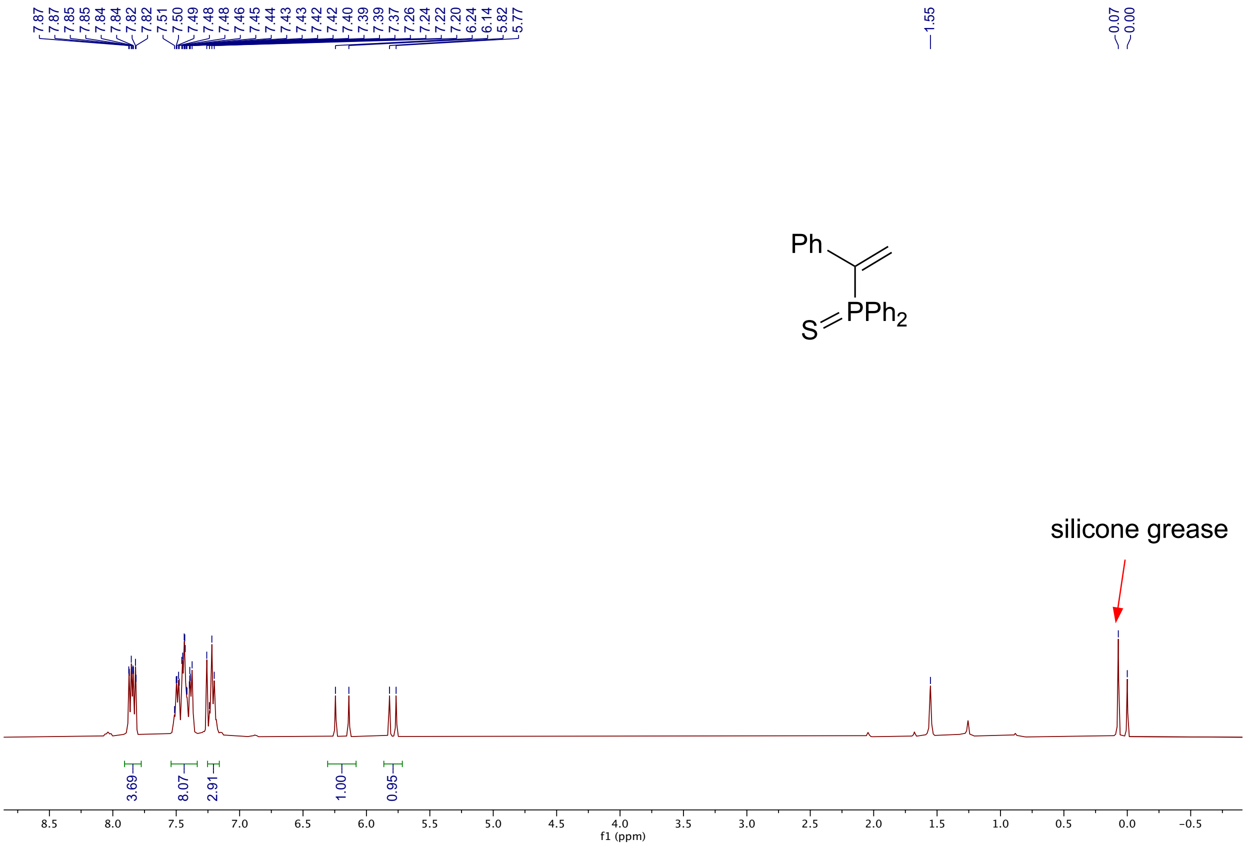

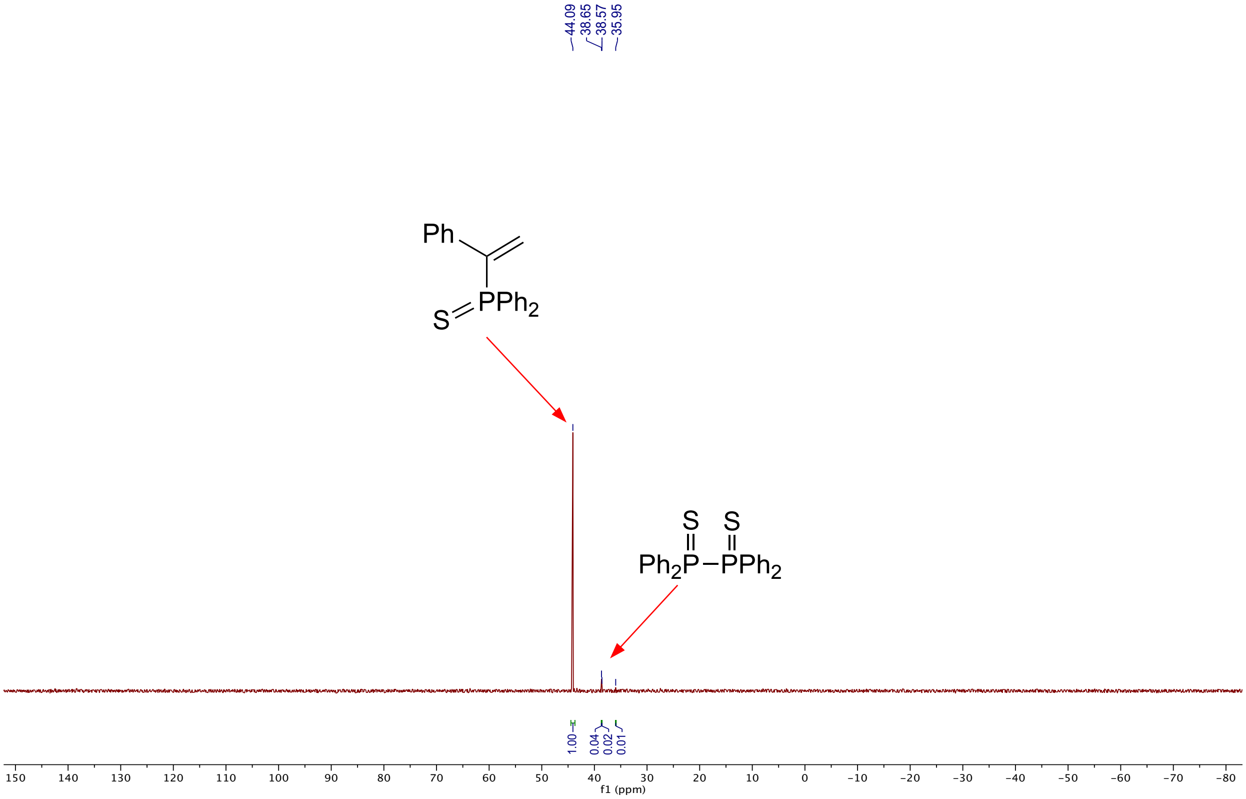

Figure S21. ^{1}H NMR (400 MHz, 23 °C, top) and ^{31}P{^{1}H} NMR (162 MHz, 23 °C, bottom) spectra of $(Ph_2PS)CPh{=}CH_2$ (~93% purity) dissolved in $CDCl_3$

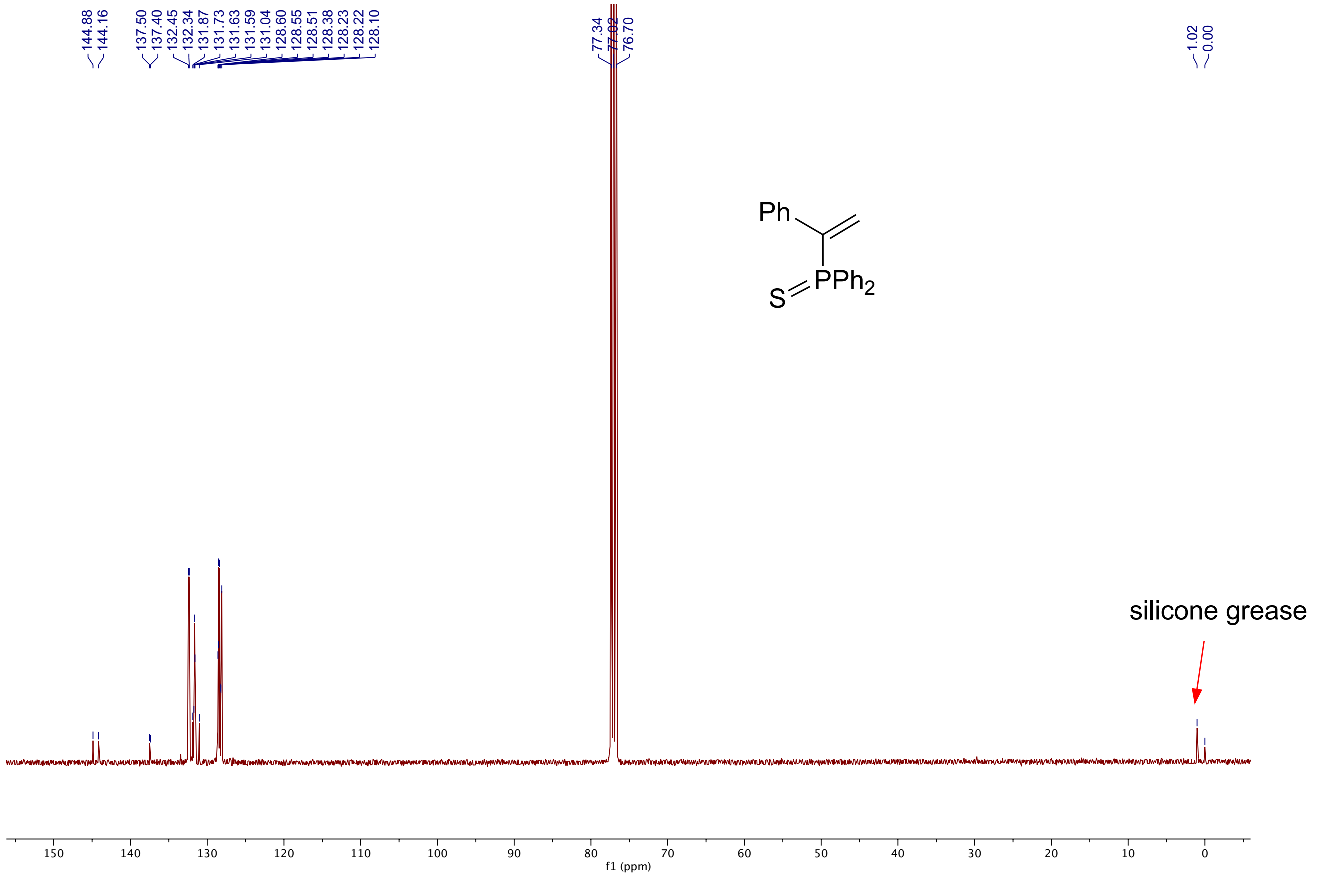

Figure S22. $^{13}C\{^{1}H\}$ NMR (101 MHz, 23 °C, $CDCl_3$) spectrum of $(Ph_2PS)CPh{=}CH_2$

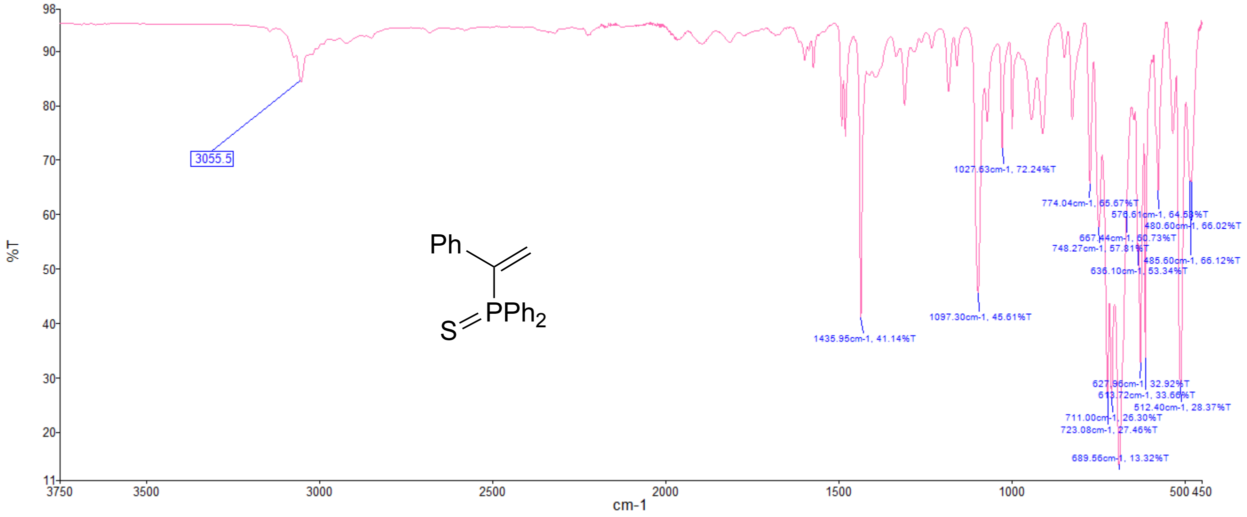

Figure S23. ATR-IR spectrum of $(Ph_2PS)CPh{=}CH_2$ (neat)

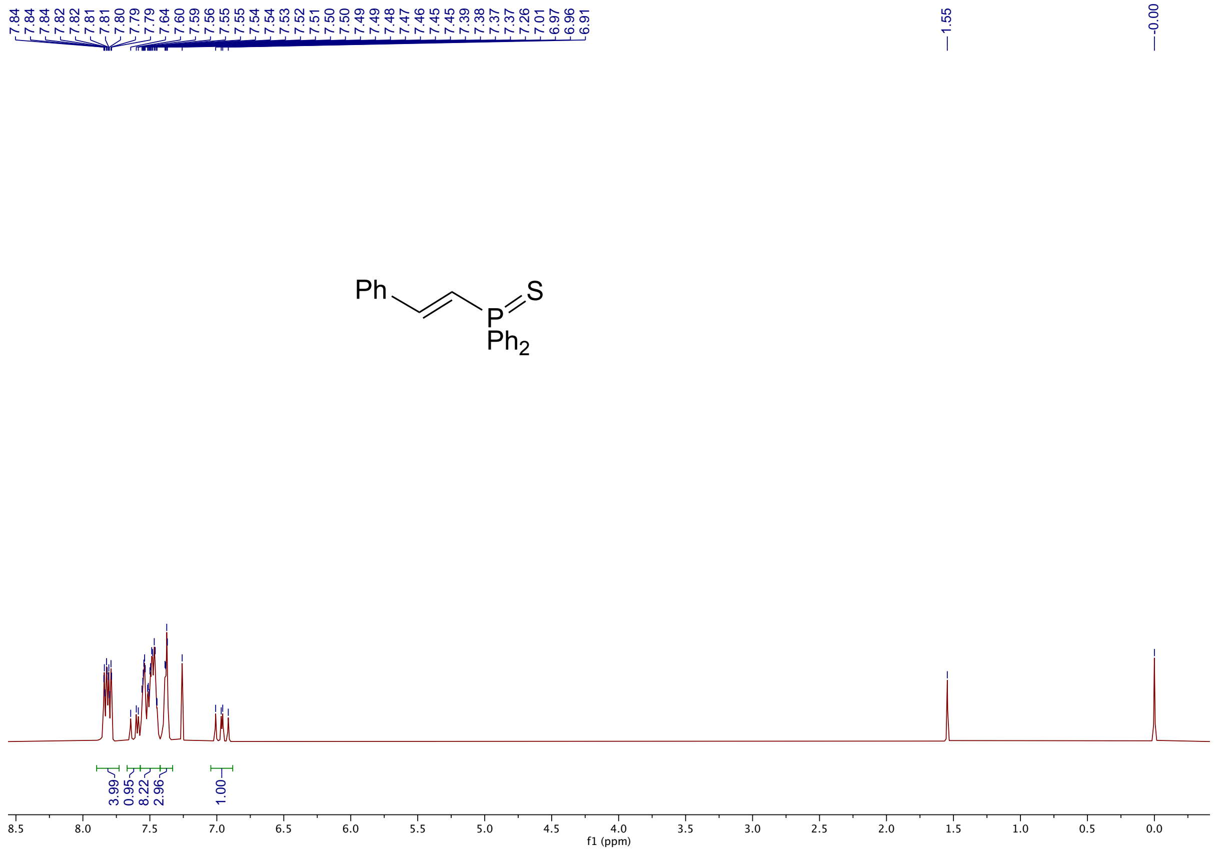

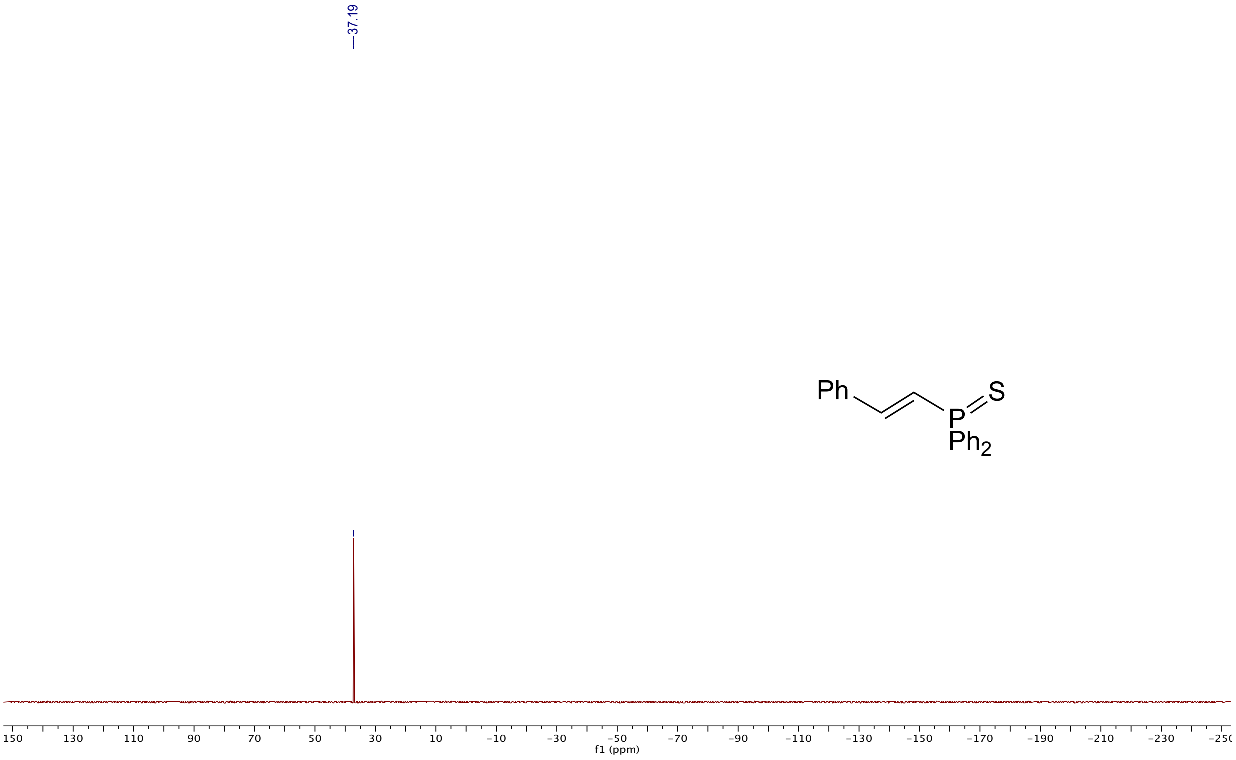

Figure S24. ^{1}H NMR (400 MHz, 23 °C, top) and $^{31}P\{^{1}H\}$ NMR (162 MHz, 23 °C, bottom) spectra of (*E*)-PhCH=CH(PSPh$_2$) dissolved in $CDCl_3$

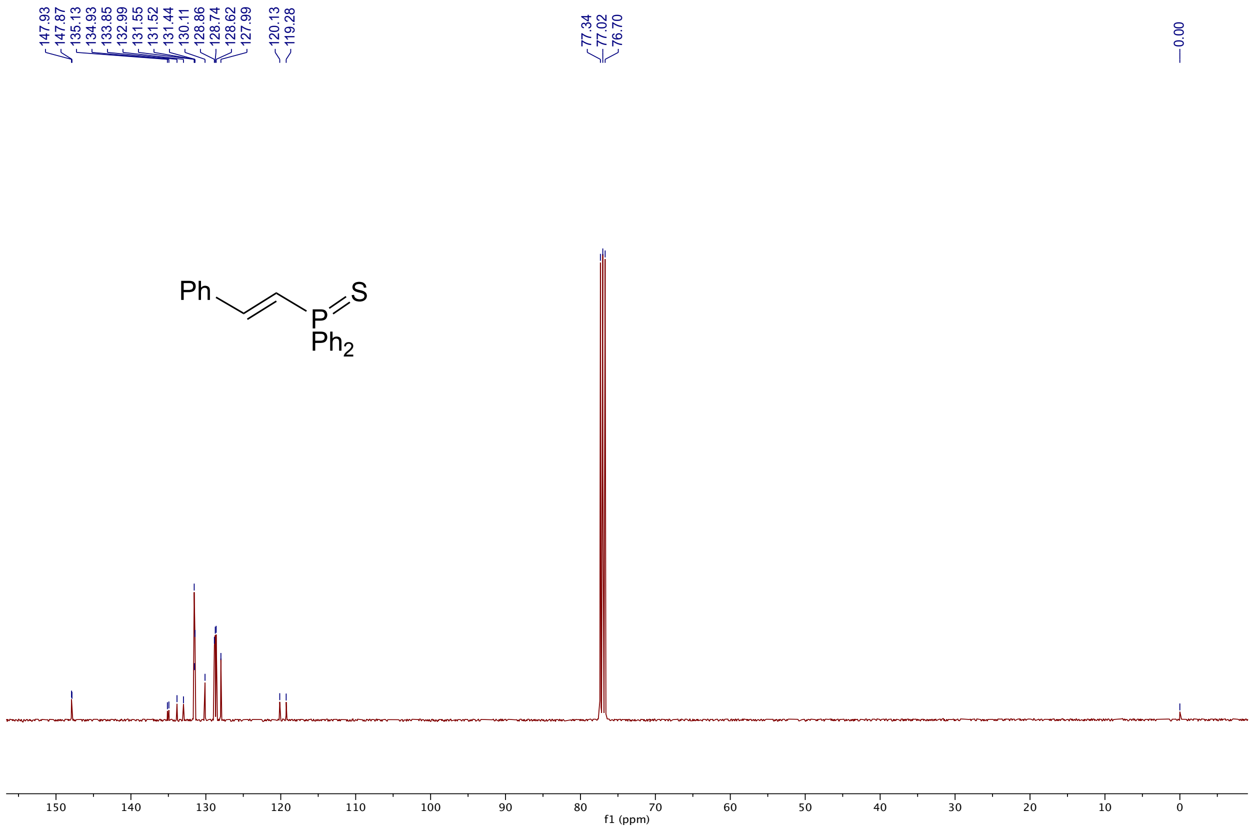

Figure S25. $^{13}C\{^1H\}$ NMR (101 MHz, 23 °C, $CDCl_3$) spectrum of (*E*)-PhCH=CH($PSPh_2$)

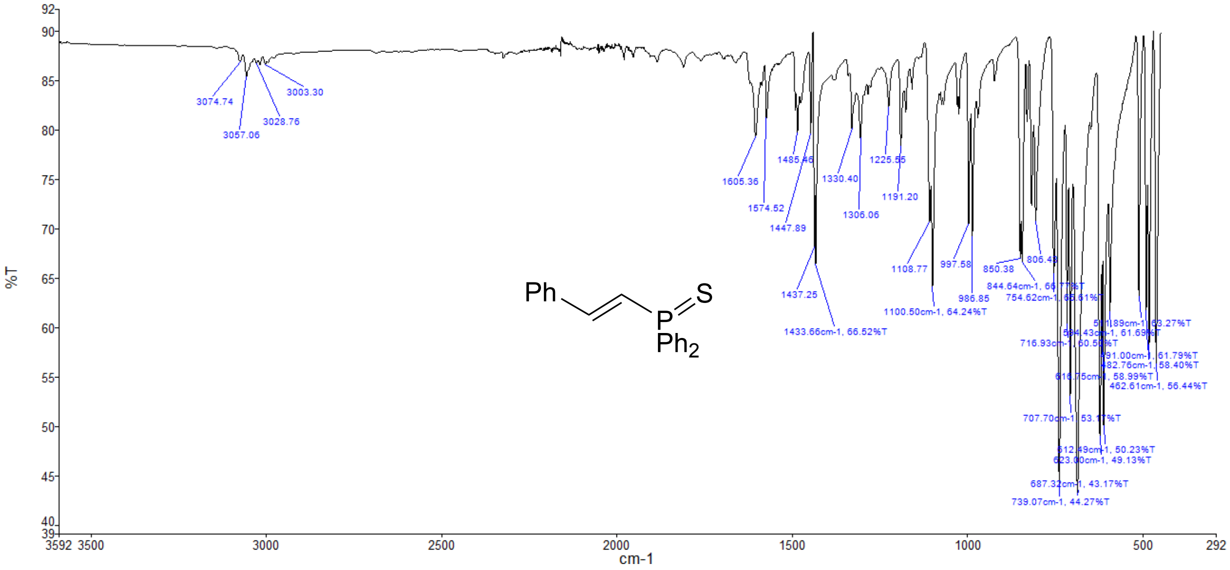

Figure S26. ATR-IR spectrum of (*E*)-PhCH=CH($PSPh_2$) (solid sample)

NMR analysis of the products generated from "Co(PMe$_3$)$_4$"-catalyzed hydrophosphination of phenylacetylene with diphenylphosphine

Method A: 80 °C reaction

The catalytic reaction was performed in toluene-d_8 under the standard conditions but without the treatment of S_8. The regioselectivity for the β-(*E*) isomer is similar (see below) and the β-(*Z*) isomer could be identified this time. The NMR assignment is made based on the data reported in the literature.[5]

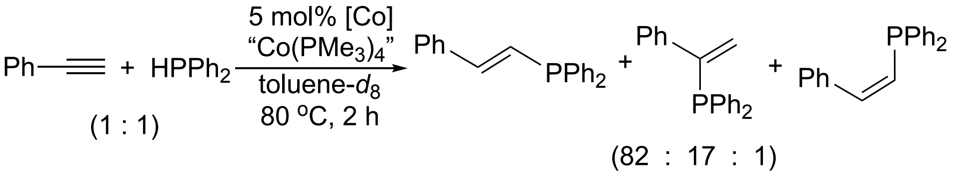

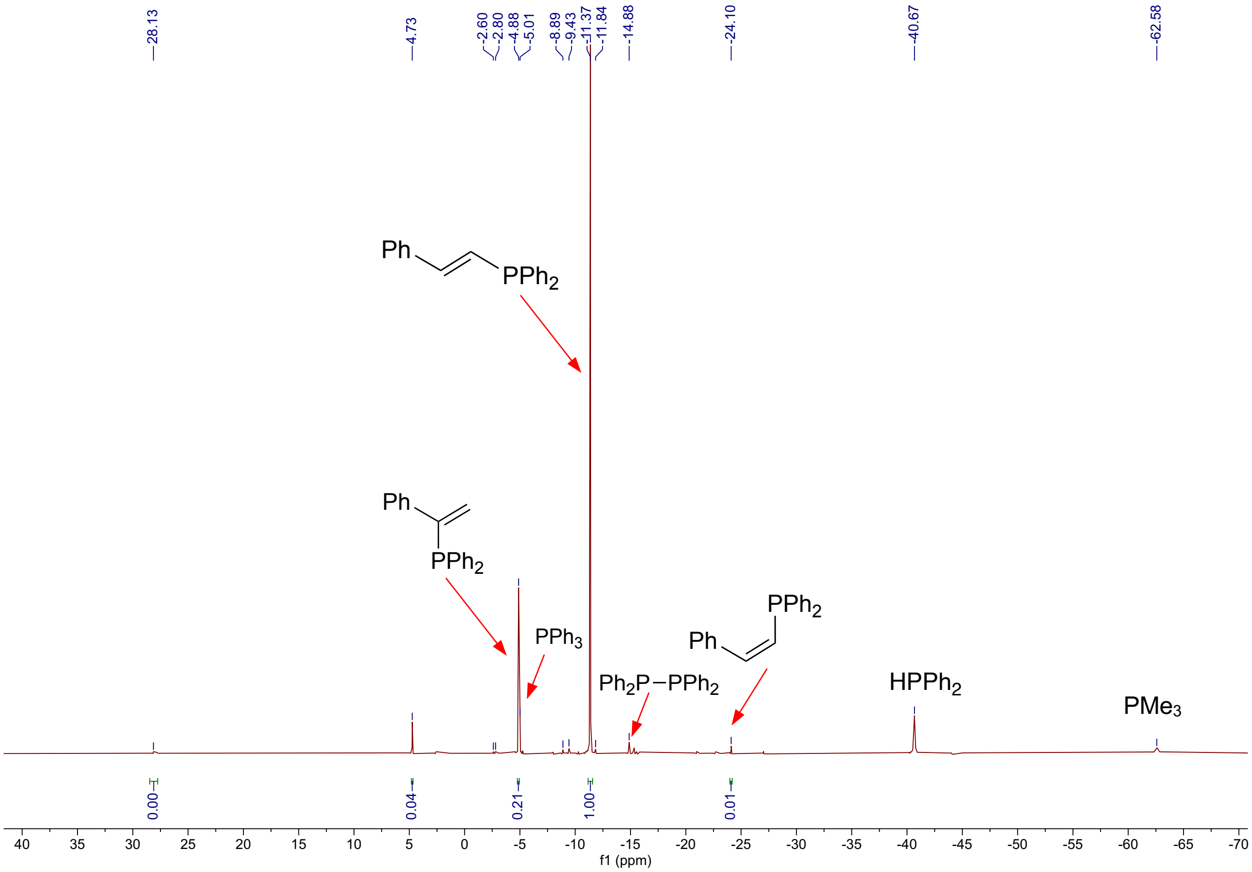

Figure S27. $^{31}P\{^1H\}$ NMR (162 MHz, 23 °C, toluene-d_8) spectrum of "Co(PMe$_3$)$_4$"-catalyzed hydrophosphination of phenylacetylene with HPPh$_2$ (performed at 80 °C)

Method B: room temperature reaction

The catalytic reaction performed at room temperature showed an improved selectivity favoring the β-(*E*) isomer, albeit more slowly.

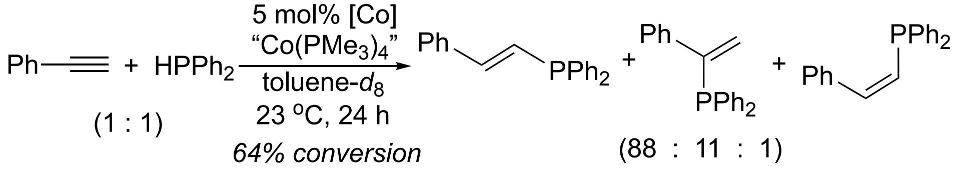

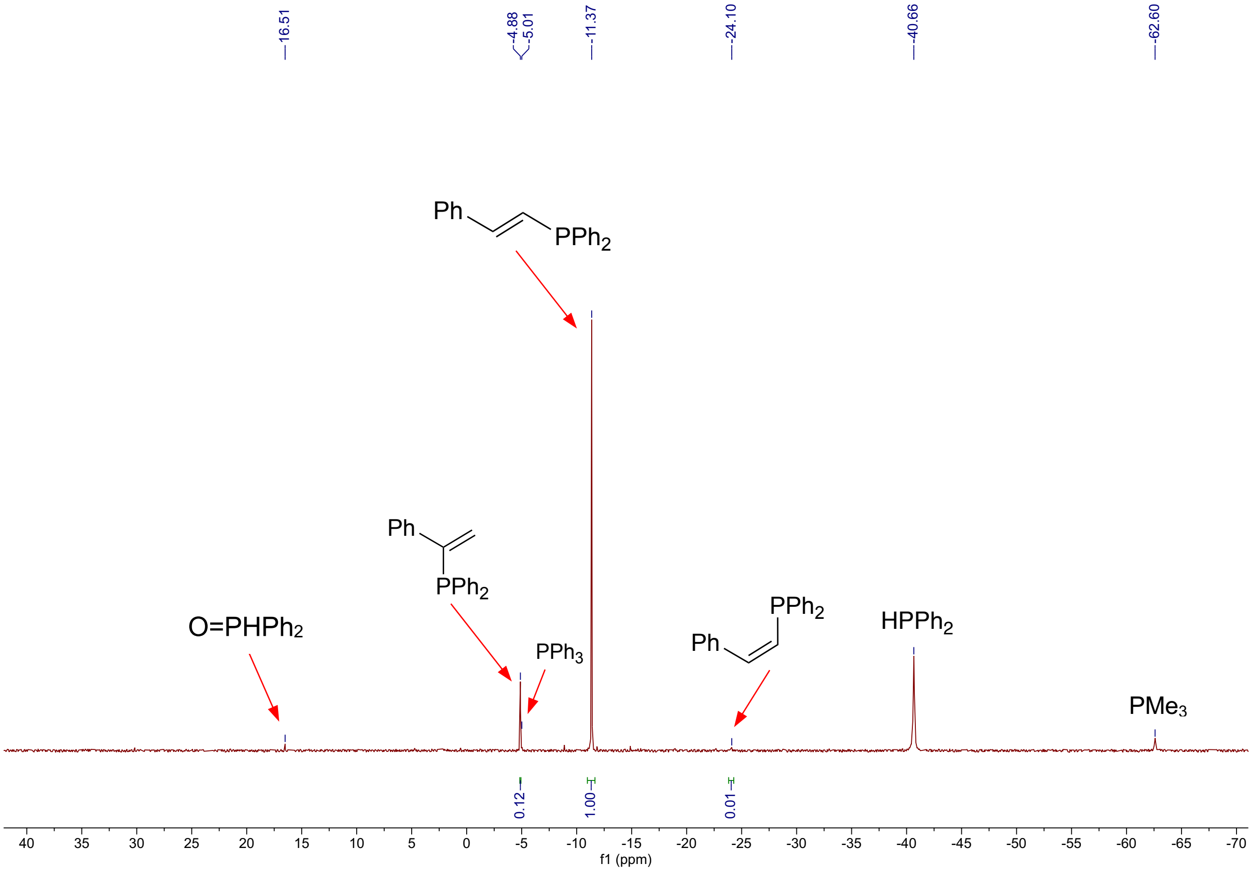

Figure S28. $^{31}P\{^{1}H\}$ NMR (162 MHz, 23 °C, toluene-d_8) spectrum of "Co(PMe_3)$_4$"-catalyzed hydrophosphination of phenylacetylene with $HPPh_2$ (performed at room temperature).

Method C: with an "aged catalyst"

The catalyst was mixed with $HPPh_2$ at 80 °C for 24 h before introducing phenylacetylene. The modified procedure resulted in slightly more β-(*Z*) isomer being formed.

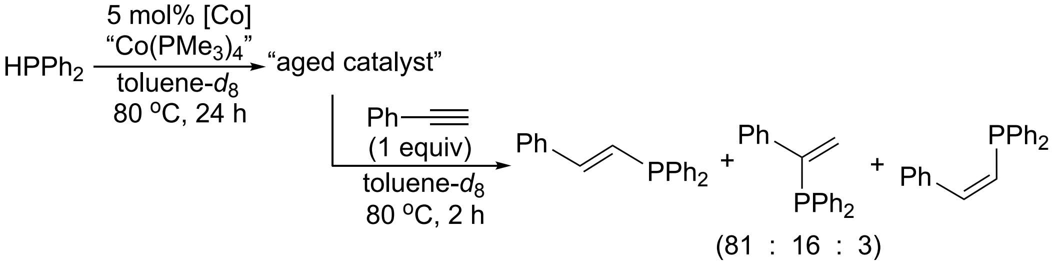

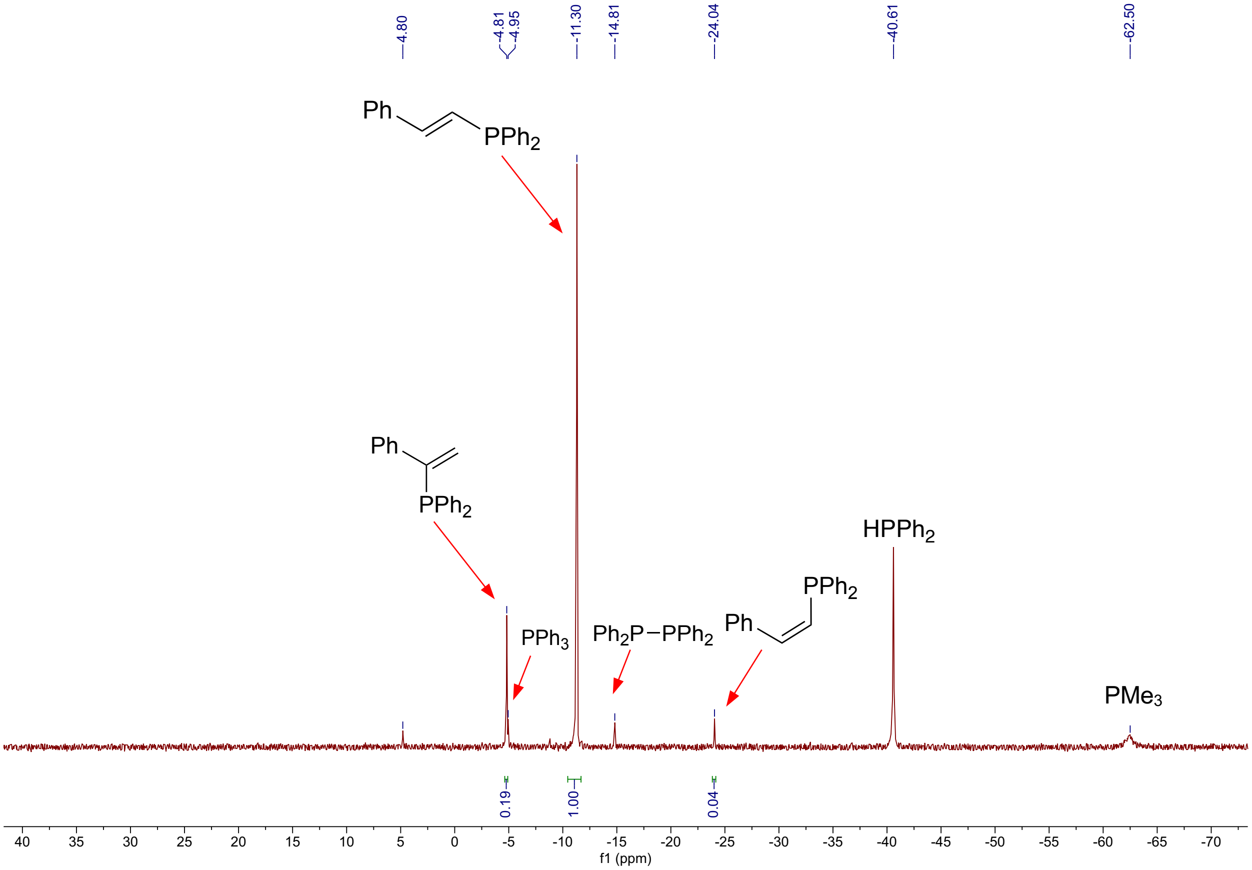

Figure S29. $^{31}P\{^{1}H\}$ NMR (162 MHz, 23 °C, toluene-*d*$_8$) spectrum of "$Co(PMe_3)_4$"-catalyzed hydrophosphination of phenylacetylene with $HPPh_2$ (performed at 80 °C but using an "aged catalyst").

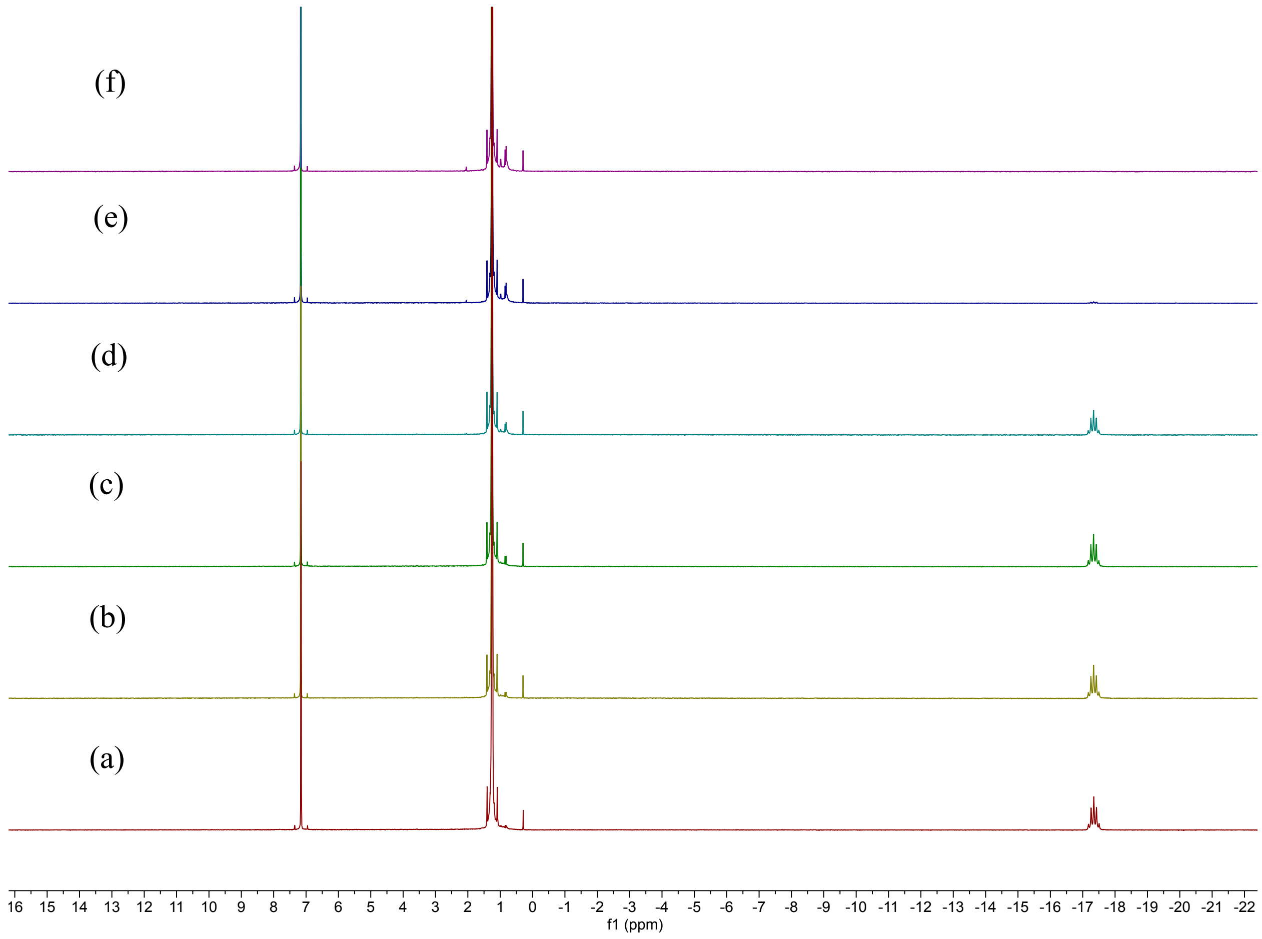

Figure S30. ^{1}H NMR (400 MHz, in C_6D_6) spectra of
(a) $HCo(PMe_3)_4$
(b) $HCo(PMe_3)_4$ kept at 23 °C for 2 h
(c) $HCo(PMe_3)_4$ kept at 23 °C for 24 h
(up to this point, no change to the NMR spectrum)
(d) $HCo(PMe_3)_4$ kept at 23 °C for 24 h and then at 80 °C for 2 h
(the intensity of Co**H** relative to C_6**H**D_5 has diminished by 40%)
(e) $HCo(PMe_3)_4$ kept at 23 °C for 24 h and then at 80 °C for 24 h
(f) $HCo(PMe_3)_4$ kept at 23 °C for 24 h and then at 80 °C for 72 h

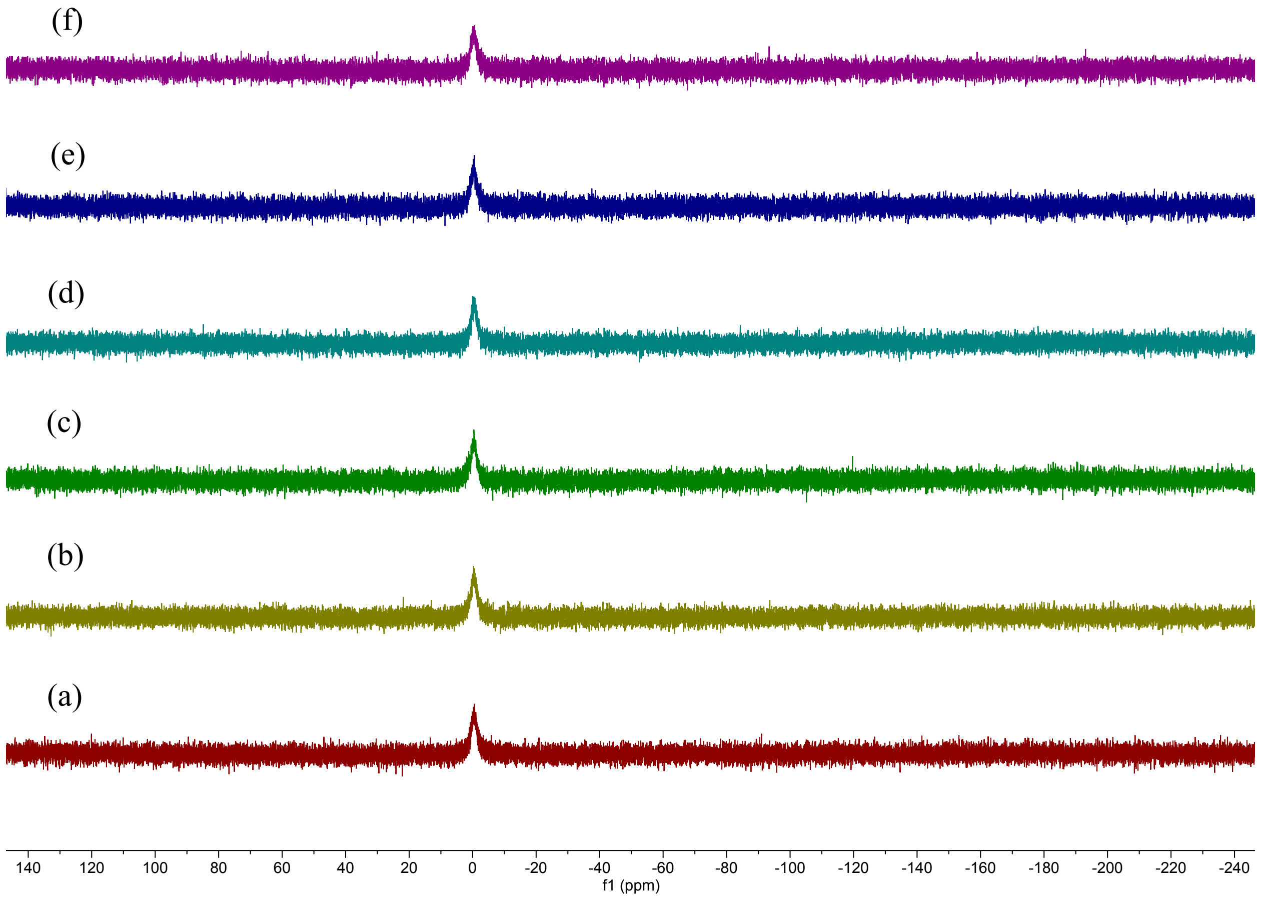

Figure S31. $^{31}P\{^{1}H\}$ NMR (162 MHz, in C_6D_6) spectra of
(a) $HCo(PMe_3)_4$
(b) $HCo(PMe_3)_4$ kept at 23 °C for 2 h
(c) $HCo(PMe_3)_4$ kept at 23 °C for 24 h
(d) $HCo(PMe_3)_4$ kept at 23 °C for 24 h and then at 80 °C for 2 h
(e) $HCo(PMe_3)_4$ kept at 23 °C for 24 h and then at 80 °C for 24 h
(f) $HCo(PMe_3)_4$ kept at 23 °C for 24 h and then at 80 °C for 72 h

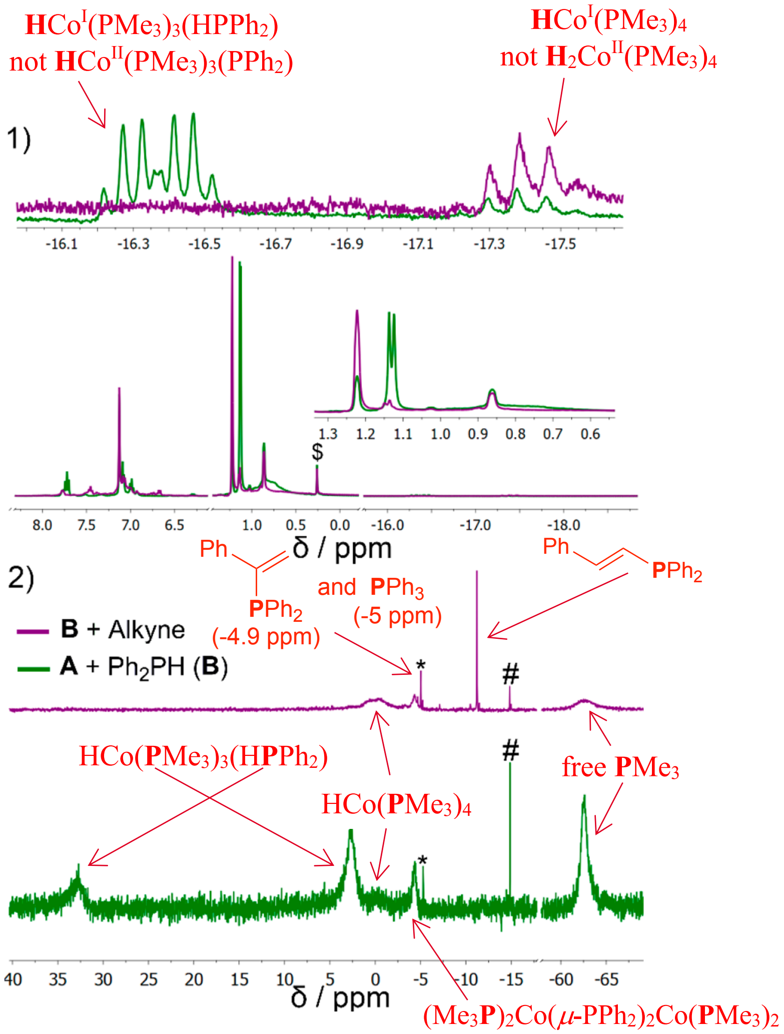

Figure 1. ^{1}H NMR (panel 1) and ^{31}P NMR (panel 2) of $[Co(PMe_3)_4]$ treated with PPh_2H (green trace) in C_6D_6. The magenta trace corresponds to substrate added into the $[Co(PMe_3)_4]$ + $HPPh_2$ reaction mixture. In both cases, the spectra were recorded in a 400 MHz NMR instrument using C_6D_6 as solvent. Panel 1 inset: The magnified region of ^{1}H NMR spectra observed between −16 and −18 ppm ($^{\$}$silicon grease). Panel 2 ($^{*}PPh_3$, $^{\#}PPh_2$-PPh_2).

Figure S32. Our annotation and re-analysis of the NMR spectra provided by Shanmugam, Shanmugam, and co-workers (Copyright 2018 American Chemical Society)[1]

Table S3. Crystal data and structure refinement for $(Me_3P)_2Co(\mu\text{-}PPh_2)_2Co(PMe_3)_2$.

**CCDC deposition number**	**CCDC-2527080**	
Formula	$C_{36}H_{56}P_6Co_2$	
Formula weight	792.48	
Temperature	150(2) K	
Wavelength	0.71073 Å	
Crystal system	Triclinic	
Space group	P-1	
Unit cell dimensions	a = 10.2854(3) Å	α = 97.9783(9)°
	b = 10.7392(3) Å	β = 94.4803(10)°
	c = 18.8916(6) Å	γ = 106.1584(8)°
Volume	1969.87(10) $Å^3$	
Z	2	
Density (calculated)	1.336 Mg/m^3	
Absorption coefficient	1.110 mm^{-1}	
F(000)	832	
Crystal size	0.118 x 0.093 x 0.052 mm^3	
θ range for data collection	2.697 to 28.290°	
Index ranges	$-13 \leq h \leq 13, -14 \leq k \leq 14, -25 \leq l \leq 25$	
Reflections collected	100949	
Independent reflections	9774 [R_{int} = 0.0264]	
Completeness to θ = 25.242°	99.8 %	
Absorption correction	Semi-empirical from equivalents	
Max. and min. transmission	0.862 and 0.839	
Refinement method	Full-matrix least-squares on F^2	
Data / restraints / parameters	9774 / 0 / 409	
Goodness-of-fit on F^2	1.050	
Final R indices [I>2σ(I)]	R1 = 0.0227, wR2 = 0.0583	
R indices (all data)	R1 = 0.0248, wR2 = 0.0598	
Largest diff. peak and hole	1.186 and -0.304 $eÅ^{-3}$	

Table S4. Bond lengths [Å] and angles [°] for $(Me_3P)_2Co(\mu\text{-}PPh_2)_2Co(PMe_3)_2$.

Co(1)-P(3)	2.1689(4)	Co(1)-P(1)	2.1773(3)
Co(1)-P(2)	2.1792(4)	Co(1)-P(1)#1	2.1829(3)
Co(1)-Co(1)#1	2.3739(3)	P(1)-C(1)	1.8364(13)
P(1)-C(7)	1.8380(13)	P(2)-C(14)	1.8310(15)
P(2)-C(13)	1.8315(14)	P(2)-C(15)	1.8372(15)
P(3)-C(17)	1.8332(15)	P(3)-C(16)	1.8423(14)
P(3)-C(18)	1.8461(16)	C(1)-C(6)	1.3905(18)
C(1)-C(2)	1.3956(18)	C(2)-C(3)	1.3851(19)
C(3)-C(4)	1.387(2)	C(4)-C(5)	1.380(2)
C(5)-C(6)	1.3918(19)	C(7)-C(12)	1.3909(18)
C(7)-C(8)	1.4011(18)	C(8)-C(9)	1.3899(19)
C(9)-C(10)	1.377(2)	C(10)-C(11)	1.383(2)
C(11)-C(12)	1.384(2)		
Co(2)-P(6)	2.1667(3)	Co(2)-P(5)	2.1768(4)
Co(2)-P(4)	2.1781(3)	Co(2)-P(4)#2	2.1809(3)
Co(2)-Co(2)#2	2.3699(3)	P(4)-C(19)	1.8424(13)
P(4)-C(25)	1.8430(13)	P(5)-C(33)	1.8308(16)
P(5)-C(32)	1.8396(16)	P(5)-C(31)	1.8480(15)
P(6)-C(35)	1.8259(14)	P(6)-C(36)	1.8318(14)
P(6)-C(34)	1.8335(14)	C(19)-C(24)	1.390(2)
C(19)-C(20)	1.4005(19)	C(20)-C(21)	1.388(2)
C(21)-C(22)	1.379(3)	C(22)-C(23)	1.376(3)
C(23)-C(24)	1.396(2)	C(25)-C(30)	1.3908(19)
C(25)-C(26)	1.3970(19)	C(26)-C(27)	1.387(2)
C(27)-C(28)	1.383(2)	C(28)-C(29)	1.379(2)
C(29)-C(30)	1.395(2)		
P(3)-Co(1)-P(1)	114.470(14)	P(3)-Co(1)-P(2)	97.286(15)
P(1)-Co(1)-P(2)	108.617(14)	P(3)-Co(1)-P(1)#1	111.968(14)
P(1)-Co(1)-P(1)#1	114.028(11)	P(2)-Co(1)-P(1)#1	109.004(14)
P(3)-Co(1)-Co(1)#1	136.378(15)	P(1)-Co(1)-Co(1)#1	57.127(10)
P(2)-Co(1)-Co(1)#1	126.318(14)	P(1)#1-Co(1)-Co(1)#1	56.901(10)
C(1)-P(1)-C(7)	96.75(6)	C(1)-P(1)-Co(1)	127.37(4)
C(7)-P(1)-Co(1)	121.62(4)	C(1)-P(1)-Co(1)#1	122.62(4)
C(7)-P(1)-Co(1)#1	123.86(4)	Co(1)-P(1)-Co(1)#1	65.972(11)
C(14)-P(2)-C(13)	100.84(7)	C(14)-P(2)-C(15)	98.10(7)
C(13)-P(2)-C(15)	98.86(7)	C(14)-P(2)-Co(1)	115.87(5)
C(13)-P(2)-Co(1)	117.57(5)	C(15)-P(2)-Co(1)	121.73(6)
C(17)-P(3)-C(16)	100.33(7)	C(17)-P(3)-C(18)	99.43(8)
C(16)-P(3)-C(18)	97.93(7)	C(17)-P(3)-Co(1)	116.11(5)
C(16)-P(3)-Co(1)	119.10(5)	C(18)-P(3)-Co(1)	120.04(6)

C(6)-C(1)-C(2)	117.77(12)	C(6)-C(1)-P(1)	122.88(10)
C(2)-C(1)-P(1)	119.32(10)	C(3)-C(2)-C(1)	121.23(12)
C(2)-C(3)-C(4)	120.14(13)	C(5)-C(4)-C(3)	119.50(13)
C(4)-C(5)-C(6)	120.14(13)	C(1)-C(6)-C(5)	121.19(13)
C(12)-C(7)-C(8)	117.11(12)	C(12)-C(7)-P(1)	118.26(10)
C(8)-C(7)-P(1)	124.53(10)	C(9)-C(8)-C(7)	120.99(13)
C(10)-C(9)-C(8)	120.63(14)	C(9)-C(10)-C(11)	119.25(13)
C(10)-C(11)-C(12)	120.16(14)	C(11)-C(12)-C(7)	121.85(13)

P(6)-Co(2)-P(5)	97.611(14)	P(6)-Co(2)-P(4)	106.499(14)
P(5)-Co(2)-P(4)	115.632(15)	P(6)-Co(2)-P(4)#2	107.134(14)
P(5)-Co(2)-P(4)#2	113.778(14)	P(4)-Co(2)-P(4)#2	114.132(11)
P(6)-Co(2)-Co(2)#2	122.150(14)	P(5)-Co(2)-Co(2)#2	140.231(14)
P(4)-Co(2)-Co(2)#2	57.123(10)	P(4)#2-Co(2)-Co(2)#2	57.008(10)
C(19)-P(4)-C(25)	95.52(6)	C(19)-P(4)-Co(2)	121.09(4)
C(25)-P(4)-Co(2)	127.99(4)	C(19)-P(4)-Co(2)#2	126.08(5)
C(25)-P(4)-Co(2)#2	122.38(4)	Co(2)-P(4)-Co(2)#2	65.869(11)
C(33)-P(5)-C(32)	100.29(8)	C(33)-P(5)-C(31)	98.55(7)
C(32)-P(5)-C(31)	98.60(8)	C(33)-P(5)-Co(2)	117.31(6)
C(32)-P(5)-Co(2)	117.98(5)	C(31)-P(5)-Co(2)	120.15(5)
C(35)-P(6)-C(36)	100.06(7)	C(35)-P(6)-C(34)	99.35(7)
C(36)-P(6)-C(34)	99.01(7)	C(35)-P(6)-Co(2)	114.97(5)
C(36)-P(6)-Co(2)	118.83(5)	C(34)-P(6)-Co(2)	120.83(5)
C(24)-C(19)-C(20)	117.46(12)	C(24)-C(19)-P(4)	120.20(10)
C(20)-C(19)-P(4)	122.19(11)	C(21)-C(20)-C(19)	120.94(15)
C(22)-C(21)-C(20)	120.55(15)	C(23)-C(22)-C(21)	119.61(14)
C(22)-C(23)-C(24)	119.98(15)	C(19)-C(24)-C(23)	121.44(14)
C(30)-C(25)-C(26)	117.84(12)	C(30)-C(25)-P(4)	123.05(10)
C(26)-C(25)-P(4)	119.09(10)	C(27)-C(26)-C(25)	120.96(14)
C(28)-C(27)-C(26)	120.31(14)	C(29)-C(28)-C(27)	119.67(14)
C(28)-C(29)-C(30)	120.01(15)	C(25)-C(30)-C(29)	121.18(14)

Symmetry transformations used to generate equivalent atoms:

#1 -x+2,-y+2,-z+2   #2 -x+1,-y+1,-z+1

Table S5. Torsion angles [°] for $(Me_3P)_2Co(\mu\text{-}PPh_2)_2Co(PMe_3)_2$.

C(7)-P(1)-C(1)-C(6)	101.23(12)	Co(1)-P(1)-C(1)-C(6)	-37.76(13)
Co(1)#1-P(1)-C(1)-C(6)	-120.89(10)	C(7)-P(1)-C(1)-C(2)	-76.88(11)
Co(1)-P(1)-C(1)-C(2)	144.13(9)	Co(1)#1-P(1)-C(1)-C(2)	61.00(11)
C(6)-C(1)-C(2)-C(3)	1.6(2)	P(1)-C(1)-C(2)-C(3)	179.83(11)
C(1)-C(2)-C(3)-C(4)	-0.5(2)	C(2)-C(3)-C(4)-C(5)	-0.9(2)
C(3)-C(4)-C(5)-C(6)	1.2(2)	C(2)-C(1)-C(6)-C(5)	-1.3(2)
P(1)-C(1)-C(6)-C(5)	-179.45(12)	C(4)-C(5)-C(6)-C(1)	-0.1(2)
C(1)-P(1)-C(7)-C(12)	162.59(10)	Co(1)-P(1)-C(7)-C(12)	-55.17(11)
Co(1)#1-P(1)-C(7)-C(12)	25.46(12)	C(1)-P(1)-C(7)-C(8)	-21.02(12)
Co(1)-P(1)-C(7)-C(8)	121.22(11)	Co(1)#1-P(1)-C(7)-C(8)	-158.15(10)
C(12)-C(7)-C(8)-C(9)	0.6(2)	P(1)-C(7)-C(8)-C(9)	-175.82(11)
C(7)-C(8)-C(9)-C(10)	-1.5(2)	C(8)-C(9)-C(10)-C(11)	1.3(2)
C(9)-C(10)-C(11)-C(12)	-0.4(2)	C(10)-C(11)-C(12)-C(7)	-0.5(2)
C(8)-C(7)-C(12)-C(11)	0.35(19)	P(1)-C(7)-C(12)-C(11)	177.01(10)
C(25)-P(4)-C(19)-C(24)	145.94(11)	Co(2)-P(4)-C(19)-C(24)	-72.76(12)
Co(2)#2-P(4)-C(19)-C(24)	8.54(13)	C(25)-P(4)-C(19)-C(20)	-38.76(12)
Co(2)-P(4)-C(19)-C(20)	102.54(11)	Co(2)#2-P(4)-C(19)-C(20)	-176.16(9)
C(24)-C(19)-C(20)-C(21)	1.1(2)	P(4)-C(19)-C(20)-C(21)	-174.35(11)
C(19)-C(20)-C(21)-C(22)	-1.2(2)	C(20)-C(21)-C(22)-C(23)	0.2(2)
C(21)-C(22)-C(23)-C(24)	0.9(2)	C(20)-C(19)-C(24)-C(23)	0.0(2)
P(4)-C(19)-C(24)-C(23)	175.51(11)	C(22)-C(23)-C(24)-C(19)	-1.0(2)
C(19)-P(4)-C(25)-C(30)	108.36(12)	Co(2)-P(4)-C(25)-C(30)	-28.84(14)
Co(2)#2-P(4)-C(25)-C(30)	-112.01(11)	C(19)-P(4)-C(25)-C(26)	-70.51(11)
Co(2)-P(4)-C(25)-C(26)	152.28(9)	Co(2)#2-P(4)-C(25)-C(26)	69.12(11)
C(30)-C(25)-C(26)-C(27)	0.8(2)	P(4)-C(25)-C(26)-C(27)	179.77(11)
C(25)-C(26)-C(27)-C(28)	0.7(2)	C(26)-C(27)-C(28)-C(29)	-1.2(2)
C(27)-C(28)-C(29)-C(30)	0.2(2)	C(26)-C(25)-C(30)-C(29)	-1.8(2)
P(4)-C(25)-C(30)-C(29)	179.27(12)	C(28)-C(29)-C(30)-C(25)	1.4(2)

Symmetry transformations used to generate equivalent atoms:

#1 -x+2,-y+2,-z+2 #2 -x+1,-y+1,-z+1

Table S6. Crystal data and structure refinement for $(Ph_2PS_2)_4Co_2$.

**CCDC deposition number**	**CCDC-2527081**	
Empirical formula	$C_{48}H_{40}P_4S_8Co_2$	
Formula weight	1115.02	
Temperature	150(2) K	
Wavelength	0.71073 Å	
Crystal system	Triclinic	
Space group	P-1	
Unit cell dimensions	a = 9.2719(4) Å	α = 94.0945(14)°
	b = 10.1792(4) Å	β = 104.2867(15)°
	c = 13.5379(7) Å	γ = 104.0279(13)°
Volume	1189.51(9) Å^3	
Z	1	
Density (calculated)	1.557 Mg/m^3	
Absorption coefficient	1.218 mm^{-1}	
F(000)	570	
Crystal size	0.116 x 0.095 x 0.093 mm^3	
θ range for data collection	2.932 to 28.319°	
Index ranges	$-12 \le h \le 12$, $-13 \le k \le 13$, $-18 \le l \le 18$	
Reflections collected	51711	
Independent reflections	5904 [R_{int} = 0.0253]	
Completeness to θ = 25.242°	99.7 %	
Absorption correction	Semi-empirical from equivalents	
Max. and min. transmission	0.862 and 0.836	
Refinement method	Full-matrix least-squares on F^2	
Data / restraints / parameters	5904 / 0 / 280	
Goodness-of-fit on F^2	1.026	
Final R indices [$I>2\sigma(I)$]	R1 = 0.0223, wR2 = 0.0568	
R indices (all data)	R1 = 0.0239, wR2 = 0.0579	
Largest diff. peak and hole	0.382 and -0.410 eÅ^{-3}	

Table S7. Bond lengths [Å] and angles [°] for $(Ph_2PS_2)_4Co_2$.

Co-S(2)	2.3296(4)	Co-S(3)	2.3331(4)
Co-S(4)#1	2.3630(4)	Co-S(1)	2.4334(4)
P(1)-C(1)	1.8087(13)	P(1)-C(7)	1.8161(14)
P(1)-S(1)	2.0059(5)	P(1)-S(2)	2.0256(5)
P(2)-C(19)	1.7972(13)	P(2)-C(13)	1.8074(14)
P(2)-S(3)	2.0110(5)	P(2)-S(4)	2.0215(5)
C(1)-C(6)	1.3892(19)	C(1)-C(2)	1.3925(19)
C(2)-C(3)	1.392(2)	C(3)-C(4)	1.380(2)
C(4)-C(5)	1.385(2)	C(5)-C(6)	1.389(2)
C(7)-C(8)	1.372(2)	C(7)-C(12)	1.375(2)
C(8)-C(9)	1.395(2)	C(9)-C(10)	1.361(3)
C(10)-C(11)	1.365(3)	C(11)-C(12)	1.389(2)
C(13)-C(18)	1.3903(19)	C(13)-C(14)	1.391(2)
C(14)-C(15)	1.386(2)	C(15)-C(16)	1.382(3)
C(16)-C(17)	1.372(2)	C(17)-C(18)	1.387(2)
C(19)-C(20)	1.3933(19)	C(19)-C(24)	1.3946(19)
C(20)-C(21)	1.3870(19)	C(21)-C(22)	1.385(2)
C(22)-C(23)	1.381(2)	C(23)-C(24)	1.391(2)

S(2)-Co-S(3)	121.771(15)	S(2)-Co-S(4)#1	132.563(14)
S(3)-Co-S(4)#1	102.348(14)	S(2)-Co-S(1)	85.862(13)
S(3)-Co-S(1)	98.804(14)	S(4)#1-Co-S(1)	105.031(14)
C(1)-P(1)-C(7)	104.96(6)	C(1)-P(1)-S(1)	112.61(5)
C(7)-P(1)-S(1)	110.78(5)	C(1)-P(1)-S(2)	111.49(5)
C(7)-P(1)-S(2)	109.83(5)	S(1)-P(1)-S(2)	107.21(2)
C(19)-P(2)-C(13)	106.99(6)	C(19)-P(2)-S(3)	111.43(5)
C(13)-P(2)-S(3)	108.55(5)	C(19)-P(2)-S(4)	111.39(5)
C(13)-P(2)-S(4)	106.88(5)	S(3)-P(2)-S(4)	111.36(2)
P(1)-S(1)-Co	81.663(15)	P(1)-S(2)-Co	83.918(16)
P(2)-S(3)-Co	93.083(16)	P(2)-S(4)-Co#1	108.689(17)
C(6)-C(1)-C(2)	119.93(12)	C(6)-C(1)-P(1)	119.66(10)
C(2)-C(1)-P(1)	120.40(11)	C(3)-C(2)-C(1)	119.66(14)
C(4)-C(3)-C(2)	120.08(14)	C(3)-C(4)-C(5)	120.47(14)
C(4)-C(5)-C(6)	119.76(14)	C(5)-C(6)-C(1)	120.10(14)
C(8)-C(7)-C(12)	118.49(14)	C(8)-C(7)-P(1)	121.38(12)
C(12)-C(7)-P(1)	120.13(11)	C(7)-C(8)-C(9)	120.89(17)
C(10)-C(9)-C(8)	119.95(18)	C(9)-C(10)-C(11)	119.66(16)
C(10)-C(11)-C(12)	120.53(16)	C(7)-C(12)-C(11)	120.47(16)
C(18)-C(13)-C(14)	119.49(13)	C(18)-C(13)-P(2)	121.69(10)
C(14)-C(13)-P(2)	118.82(11)	C(15)-C(14)-C(13)	119.69(15)
C(16)-C(15)-C(14)	120.40(16)	C(17)-C(16)-C(15)	120.13(15)
C(16)-C(17)-C(18)	120.15(15)	C(17)-C(18)-C(13)	120.13(14)
C(20)-C(19)-C(24)	120.40(12)	C(20)-C(19)-P(2)	119.24(10)
C(24)-C(19)-P(2)	120.34(10)	C(21)-C(20)-C(19)	119.53(14)

C(22)-C(21)-C(20)	120.09(14)	C(23)-C(22)-C(21)	120.45(13)
C(22)-C(23)-C(24)	120.21(14)	C(23)-C(24)-C(19)	119.30(14)

Symmetry transformations used to generate equivalent atoms: #1 -x+1,-y+1,-z+2

Table S8. Torsion angles [°] for $(Ph_2PS_2)_4Co_2$.

C(7)-P(1)-C(1)-C(6)	-92.98(12)	S(1)-P(1)-C(1)-C(6)	27.61(12)
S(2)-P(1)-C(1)-C(6)	148.16(10)	C(7)-P(1)-C(1)-C(2)	85.72(12)
S(1)-P(1)-C(1)-C(2)	-153.68(10)	S(2)-P(1)-C(1)-C(2)	-33.13(12)
C(6)-C(1)-C(2)-C(3)	0.7(2)	P(1)-C(1)-C(2)-C(3)	-177.96(11)
C(1)-C(2)-C(3)-C(4)	-0.9(2)	C(2)-C(3)-C(4)-C(5)	0.4(2)
C(3)-C(4)-C(5)-C(6)	0.1(2)	C(4)-C(5)-C(6)-C(1)	-0.2(2)
C(2)-C(1)-C(6)-C(5)	-0.2(2)	P(1)-C(1)-C(6)-C(5)	178.53(11)
C(1)-P(1)-C(7)-C(8)	-14.38(18)	S(1)-P(1)-C(7)-C(8)	-136.18(17)
S(2)-P(1)-C(7)-C(8)	105.59(17)	C(1)-P(1)-C(7)-C(12)	165.79(14)
S(1)-P(1)-C(7)-C(12)	43.99(15)	S(2)-P(1)-C(7)-C(12)	-74.24(15)
C(12)-C(7)-C(8)-C(9)	-0.5(4)	P(1)-C(7)-C(8)-C(9)	179.7(2)
C(7)-C(8)-C(9)-C(10)	1.2(4)	C(8)-C(9)-C(10)-C(11)	-1.6(4)
C(9)-C(10)-C(11)-C(12)	1.2(3)	C(8)-C(7)-C(12)-C(11)	0.1(3)
P(1)-C(7)-C(12)-C(11)	179.93(17)	C(10)-C(11)-C(12)-C(7)	-0.5(3)
C(19)-P(2)-C(13)-C(18)	7.07(13)	S(3)-P(2)-C(13)-C(18)	127.42(11)
S(4)-P(2)-C(13)-C(18)	-112.36(11)	C(19)-P(2)-C(13)-C(14)	-174.23(13)
S(3)-P(2)-C(13)-C(14)	-53.88(13)	S(4)-P(2)-C(13)-C(14)	66.34(13)
C(18)-C(13)-C(14)-C(15)	-0.2(3)	P(2)-C(13)-C(14)-C(15)	-178.98(16)
C(13)-C(14)-C(15)-C(16)	1.1(3)	C(14)-C(15)-C(16)-C(17)	-0.7(3)
C(15)-C(16)-C(17)-C(18)	-0.6(3)	C(16)-C(17)-C(18)-C(13)	1.5(2)
C(14)-C(13)-C(18)-C(17)	-1.0(2)	P(2)-C(13)-C(18)-C(17)	177.64(11)
C(13)-P(2)-C(19)-C(20)	-74.91(12)	S(3)-P(2)-C(19)-C(20)	166.59(9)
S(4)-P(2)-C(19)-C(20)	41.56(12)	C(13)-P(2)-C(19)-C(24)	103.75(12)
S(3)-P(2)-C(19)-C(24)	-14.75(12)	S(4)-P(2)-C(19)-C(24)	-139.78(10)
C(24)-C(19)-C(20)-C(21)	-0.8(2)	P(2)-C(19)-C(20)-C(21)	177.84(11)
C(19)-C(20)-C(21)-C(22)	1.3(2)	C(20)-C(21)-C(22)-C(23)	-0.9(2)
C(21)-C(22)-C(23)-C(24)	0.1(2)	C(22)-C(23)-C(24)-C(19)	0.3(2)
C(20)-C(19)-C(24)-C(23)	0.0(2)	P(2)-C(19)-C(24)-C(23)	-178.61(11)

Symmetry transformations used to generate equivalent atoms: #1 -x+1,-y+1,-z+2

References

(1) Rajpurohit, J.; Kumar, P.; Shukla, P.; Shanmugam, M.; Shanmugam, M. Mechanistic Investigation of Well-Defined Cobalt Catalyzed Formal *E*-Selective Hydrophosphination of Alkynes. *Organometallics* **2018**, *37*, 2297-2304.

(2) Wang, M.-K.; Luo, Y.-C.; Zhao, H.-Y.; Zhang, Y.; Zhang, D.; Zhang, X. Modular Diastereoselective Construction of Polysubstituted Cyclopentanes Enabled by Cobalt-Catalyzed Arylfluoroalkylation of Cyclopentenes. *ACS Catal.* **2023**, *13*, 14090-14102.

(3) Mills, L. R.; Kim, J.; Simmons, E. M.; Wisniewski, S. R.; Chirik, P. J. C(sp^3)−C(sp^3) Reductive Elimination from (Phenoxyimine)Cobalt(III)$(CH_3)_2(PMe_3)_2$ Complexes. *Organometallics* **2024**, *43*, 1021-1029.

(4) Beck, R.; Klein, H.-F. Bis(μ-diphenylphosphanyl)bis[(trimethylphosphane)cobalt(I)](Co–Co). *Acta Cryst. E.* **2013**, *69*, m604.

(5) (a) Di Giuseppe, A.; De Luca, R.; Castarlenas, R.; Pérez-Torrente, J. J.; Crucianelli, M.; Oro, L. A. Double Hydrophosphination of Alkynes Promoted by Rhodium: the Key Role of an *N*-Heterocyclic Carbene Ligand. *Chem. Commun.* **2016**, *52*, 5554-5557. (b) Tolentino, D. R.; Neale, S. E.; Isaac, C. J.; Macgregor, S. A.; Whittlesey, M. K.; Jazzar, R.; Bertrand, G. Reductive Elimination at Carbon under Steric Control. *J. Am. Chem. Soc.* **2019**, *141*, 9823-9826.
